# The Comparative Osteology of the Petrotympanic Complex (Ear Region) of Extant Baleen Whales (Cetacea: Mysticeti)

**DOI:** 10.1371/journal.pone.0021311

**Published:** 2011-06-22

**Authors:** Eric G. Ekdale, Annalisa Berta, Thomas A. Deméré

**Affiliations:** 1 Department of Paleontology, San Diego Natural History Museum, San Diego, California, United States of America; 2 Department of Biology, San Diego State University, San Diego, California, United States of America; University of Lethbridge, Canada

## Abstract

**Background:**

Anatomical comparisons of the ear region of baleen whales (Mysticeti) are provided through detailed osteological descriptions and high-resolution photographs of the petrotympanic complex (tympanic bulla and petrosal bone) of all extant species of mysticete cetaceans. Salient morphological features are illustrated and identified, including overall shape of the bulla, size of the conical process of the bulla, morphology of the promontorium, and the size and shape of the anterior process of the petrosal. We place our comparative osteological observations into a phylogenetic context in order to initiate an exploration into petrotympanic evolution within Mysticeti.

**Principal Findings:**

The morphology of the petrotympanic complex is diagnostic for individual species of baleen whale (e.g., sigmoid and conical processes positioned at midline of bulla in *Balaenoptera musculus*; confluence of fenestra cochleae and perilymphatic foramen in *Eschrichtius robustus*), and several mysticete clades are united by derived characteristics. Balaenids and neobalaenids share derived features of the bulla, such as a rhomboid shape and a reduced anterior lobe (swelling) in ventral aspect, and eschrichtiids share derived morphologies of the petrosal with balaenopterids, including loss of a medial promontory groove and dorsomedial elongation of the promontorium. Monophyly of Balaenoidea (Balaenidae and Neobalaenidae) and Balaenopteroidea (Balaenopteridae and Eschrichtiidae) was recovered in phylogenetic analyses utilizing data exclusively from the petrotympanic complex.

**Significance:**

This study fills a major gap in our knowledge of the complex structures of the mysticete petrotympanic complex, which is an important anatomical region for the interpretation of the evolutionary history of mammals. In addition, we introduce a novel body of phylogenetically informative characters from the ear region of mysticetes. Our detailed anatomical descriptions, illustrations, and comparisons provide valuable data for current and future studies on the phylogenetic relationships, evolution, and auditory physiology of mysticetes and other cetaceans throughout Earth's history.

## Introduction

The mammalian petrotympanic complex houses the organs of hearing and balance and in cetaceans is partially or completely detached from the rest of the basicranium [1], [2]. The petrotympanic complex of mysticetes (baleen whales) shares a number of derived features with that of odontocetes (toothed whales), but differs in several significant respects [3]. Like odontocetes, the mysticete petrotympanic complex consists of two main bony elements, the tympanic and petrosal bones, that surround and articulate with the three middle ear ossicles: malleus, incus, and stapes. The petrosal portion of the petrotympanic complex houses the inner ear (hearing receptors) as well as the vestibular organ and semicircular canals, while the tympanic portion of the complex contributes to the resonant tympanic cavity and forms the ventral portion of the middle ear region.

**Figure 1:**
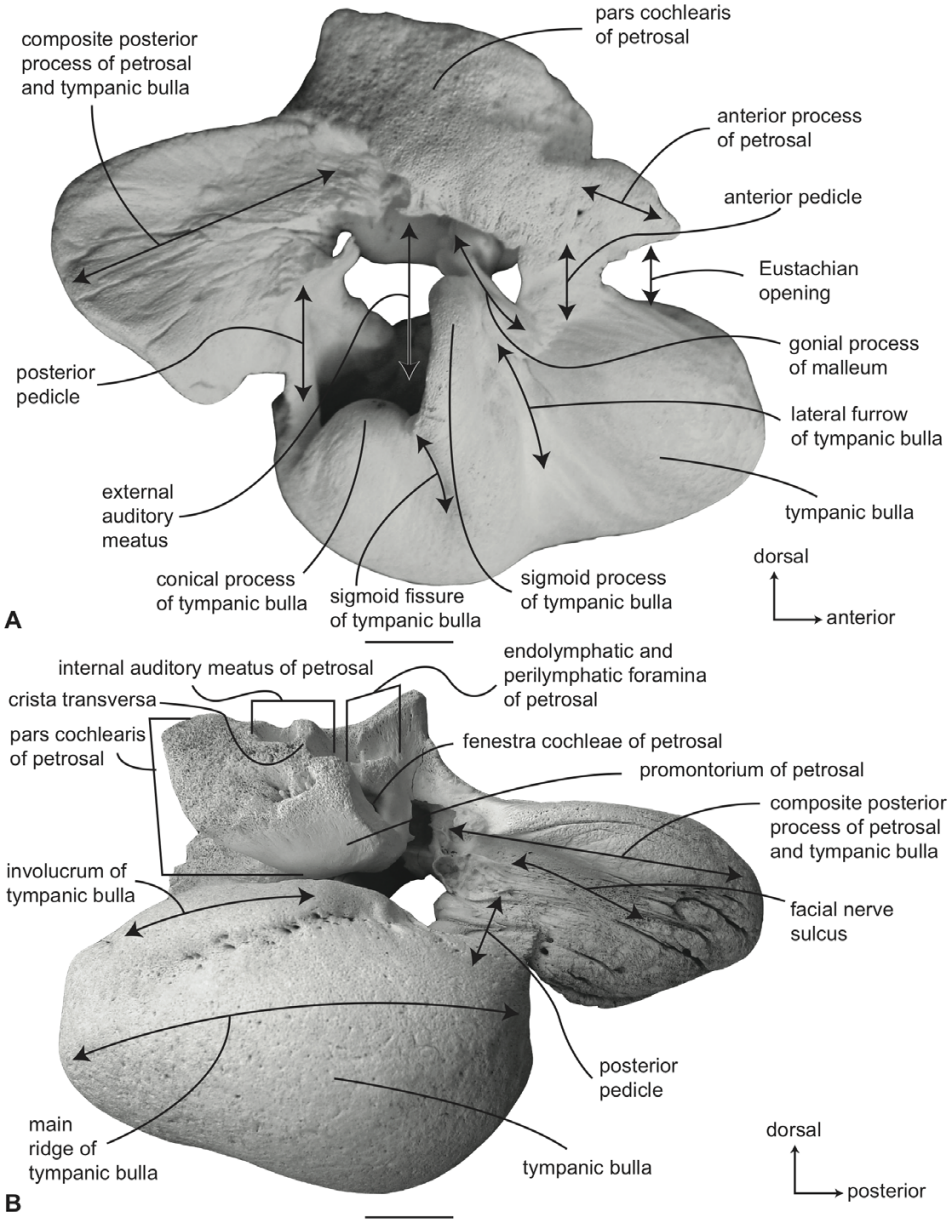
Gross anatomy of petrotympanic complex of a mysticete (juvenile *Eschrichtius robustus*, SDSNH 23762). A. lateral, and B. medial views. Scale bar  = 2 cm.

Unlike the odontocete petrotympanic complex, the petrotympanic bones in mysticetes are firmly attached to one another via two bony connections: the anterior and posterior pedicles (Figure 1). Development of an anterior pedicle is derived for mysticetes, and it is absent in odontocetes which instead possess an accessory ossicle between the bulla and petrosal. Further, the mysticete petrotympanic complex is tightly wedged between the squamosal and exoccipital bones by way of the composite posterior process (fusion of the petrosal and tympanic bulla), which contrasts with the condition in most odontocetes where the petrotympanic complex is completely detached from the basicranium and suspended by a ligamental sling within the cranial hiatus [4]–[6]. The odontocete tympanic bulla is also open anteriorly via the Eustachian opening, while in mysticetes and archaeocetes the anterior margin of the bulla forms a complete wall with the Eustachian opening on the ventral aspect of the bulla. In addition to anatomical differences in the petrotympanic complex between mysticetes and odontocetes there are also differences in their hearing frequencies [7], [8], with odontocetes utilizing ultransonic/high frequency hearing, while mysticetes are considered infrasonic/low frequency hearers [6]–[8].

Not surprisingly, the mysticete petrotympanic bones comprise a morphologically and functionally complex structure that preserves a wealth of anatomical information useful in examining patterns of adaptation and macroevolution. As recently discussed by O'Leary [9], any attempt to decipher the historical sequence of anatomical changes in the cetacean ear region requires a solid understanding of the petrotympanic anatomy of cetaceans and their extant and extinct relatives. To this end O'Leary presented a comprehensive osteological atlas of the petrosals of a taxonomically broad group of extant and extinct artiodactylans (traditional artiodactyls plus cetaceans) and compiled an annotated listing of salient characters to be used in current and future phylogenetic analyses [9]. Although focused only on the petrosal, the study of O'Leary builds on the important earlier work of Luo and Gingerich [2], which examined the homologies of the petrotympanic complex in mesonychians and stem-cetaceans to outline the transformation of the cetacean basicranium and the evolution of aquatic hearing in archaeocetes and crown cetaceans. However, neither of these studies incorporated many mysticetes in their descriptions and discussions (Luo and Gingerich included two extant and three extinct mysticetes, and O'Leary only examined one).

Other important works utilizing a comparative anatomical approach to investigate cetacean hearing adaptations and evolution include research on the tympanic bulla of stem cetaceans [10], the petrosal of a herpetocetine mysticete [11], the petrosal of a fossil kogiine odontocete [12], the petrosal of a squalodontid odontocete [13], the petrosal and tympanic bulla of odontocetes [14],the functional anatomy of cetacean petrotympanics over geologic time [15], the tympanic bullae of fossil mysticetes [16], and the petrosal and tympanic bullae of extant odontocetes [17]. To this can be added a large number of additional taxonomic studies focused on alpha level descriptions of new species of fossil cetaceans that include comparative descriptions of the morphology of preserved petrosals and/or tympanic bullae [18]–[24].

A cursory review of the literature reveals a somewhat confusing array of anatomical terms for the various salient morphological parts and regions of the mysticete petrotympanic complex. Although several recent reports [9], [25] have gone a long way to remedy this situation, these studies focused largely on odontocetes and/or related groups other than mysticetes. The same can be said for the classic comparative study of Kasuya [5], which focused on the petrotympanic complex in all extant odontocetes. That study presented a comprehensive review of odontocete petrotympanic morphology, provided a photographic atlas with images the petrosals and tympanic bullae of all extant species, summarized odontocete phylogenetic relationships based on overall similarity of petrotympanic characters, and presented a dichotomous key. Subsequent workers have used Kasuya's results in a variety of evolutionary and functional studies and his work continues to be cited by scientists investigating the physiology and evolutionary history of cetaceans.

With over 35 years elapsing since the seminal study by Kasuya [5] and no similar work having been done on mysticetes we felt it time to develop a comprehensive morphological atlas for the mysticete petrotympanic complex that provides comparative osteological descriptions of all extant mysticetes, high-resolution digital images of their petrosals and tympanic bullae in four standard views, and a summary of phylogenetically relevant morphological characters. In providing these detailed anatomical descriptions, we recognize the importance of the mysticete petrotympanic complex in functional and phylogenetic analyses. As a demonstration of the strong phylogenetic signal preserved in the petrotympanic complex, we identified 48 morphological characters from this anatomical region, scored them for all extant mysticete species (i.e. Balaenidae, Neobalaenidae, and Balaenopteroidea: Balaenopteridae + Eschrichtiidae; Table S1), and conducted a phylogenetic analysis. We then used the resulting phylogeny to test three currently proposed and competing hypotheses of higher-level mysticete relationships. Finally, we provide a dichotomous key for extant species of baleen whales (Text S1) in order to demonstrate the diagnostic significance of the mysticete petrotympanic complex.

All of the specimens that were examined are housed in curated museum collections (Table S1). The specific collections that were utilized were those of the American Museum of Natural History, New York, New York (AMNH), Historical Museum of Tesho River, Japan (HMT), Humboldt State University, Arcata, California (HSU VM), Royal Institute of Natural Sciences, Belgium (IRSNB), Natural History Museum of Los Angeles County, Los Angeles, California (LACM), National Science Museum, Tokyo, Japan (NSMT), South Australia Museum, Adelaide, Australia (SAM), San Diego Natural History Museum, San Diego, California (SDSNH), San Diego State University, Mammal Collections, San Diego, California (SDSU), University of California, Museum of Paleontology, Berkeley, California (UCMP), United States National Museum of Natural History, Smithsonian Institution, Washington, D.C. (USNM), and Zoological Museum, University of Copenhagen, Copenhagen, Denmark (ZMUC).

### Introduction to Mysticete Petrotympanic Anatomy

As with terrestrial mammals, the mysticete ear is divided into three major anatomical divisions: the outer ear from the external environment to the tympanic membrane, the middle ear from the tympanic membrane to the ventrolateral (tympanic) surface of the petrosal, and the inner ear including the organs of balance and hearing within the petrosal bone itself. Although the present study is focused on the morphology of the middle ear and its structures in mysticetes, we also provide an overview of the outer and inner ears as an introduction to the entire system.

**Figure 2:**
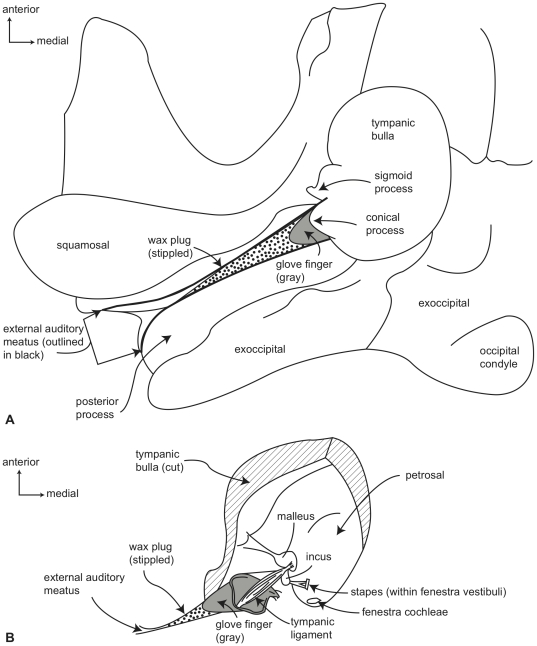
Gross anatomy of right ear region of a generalized mysticete in ventral view. A. basicranium with soft tissues of the outer ear (wax plug, glove finger) reconstructed (following personal observation supplemented by published descriptions [26], [28]), B. detail with bulla partially removed to reveal tympanic ligament and ossicles (redrawn and modified from Purves [28], fig. 14]).

**Figure 3:**
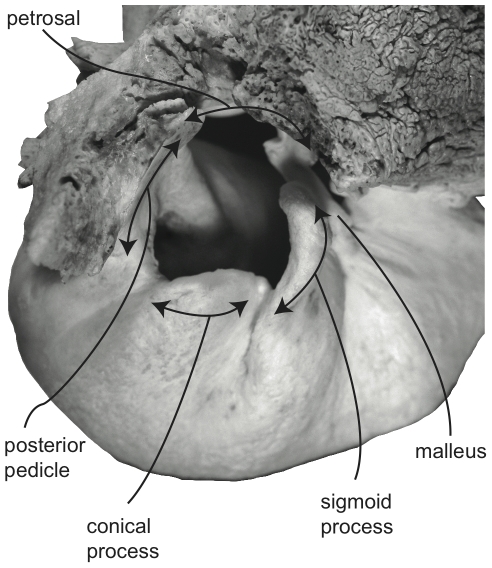
Detail of right tympanic annulus (external view) of *Megaptera novaeangliae* (VM 1842).

**Figure 4:**
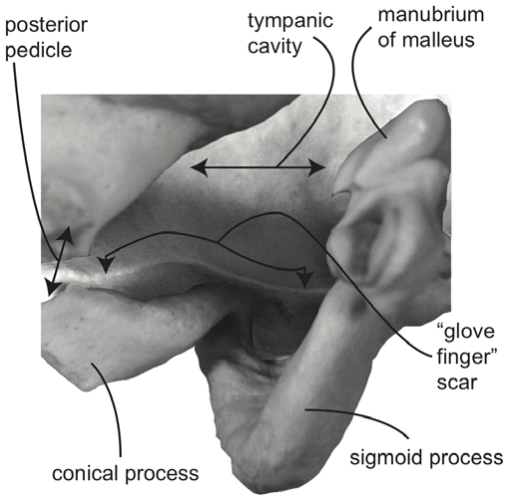
Detail of tympanic annulus (internal view) of *Megaptera novaeangliae* (VM 2776; image reversed).

Unlike the outer ear of terrestrial mammals, all extant cetaceans lack an external pinna surrounding the external auditory meatus, which connects the tympanic membrane to the surrounding environment. At the proximal end of the mysticete external auditory meatus is a dense waxy plug that nearly fills the entire lumen of the meatus (Figure 2A). The proximal end of this unique structure is concave and invaded by the distal end of the highly modified tympanic membrane, which in mysticetes is an elongate, sack-like structure (the so-called “glove finger”; Figure 2B) composed of white fibrous tissue and yellow elastic tissue [26]. This conical homologue of the traditional flattened mammalian tympanic membrane [4], [27] (although may only be homologous to part of the membrane [10]) is attached to the tympanic annulus, which is primarily formed by the sigmoid process and the adjacent conical process and posterior pedicle of the tympanic bulla (Figure 3). A stout tympanic ligament extends from the internal surface of the glove finger to attach to the manubrium of the malleus (Figure 2B), which in turn is firmly attached to the sigmoid process via the delicate processus gracilis (Figure 1). Within the tympanic cavity of the bulla the area of attachment is marked by a low, thin, curvilinear ridge that extends from near the apex of the posterior face of the sigmoid process, passes around and beneath the conical process, and ascends the anterior face of the posterior pedicle (Figure 4).

Within the middle ear, the bullar portion of the tympanic cavity is formed by the deeply excavated region between the involucrum and lateral wall of the tympanic bulla (Figure 1). Numerous, deep transverse creases are developed on the involucrum, and are especially prominent anteriorly. *In vivo* this region of the tympanic cavity is occupied by a vascular structure called the corpus cavernosum tympanicum that may expand to fill the bullar cavity during diving [28]. The roof of the tympanic cavity is formed by the ventral surface of the petrosal (Figure 1), including the epitympanic recess (the region of the petrosal lateral to the fenestra vestibuli and occupied by the ear ossicles). Important anatomical structures of the petrosal contribution to the middle ear include the promontorium, fenestra cochleae, fenestra vestibuli, secondary facial foramen, facial sulcus, stapedial fossa, malleolar fossa, stapes, incus, and malleus (Figures 1, 2, 3, 4).

In mysticetes the promontorium of the petrosal is typically domed and not flattened as in odontocetes; the fenestra cochleae and fenestra vestibuli are well separated from each other as in odontocetes and unlike the condition in terrestrial artiodactyls where the two openings are more closely positioned; the secondary facial foramen and facial sulcus is generalized in its form and position (with the exception of *Caperea*); the stapedial fossa is often large and hemispherical; the malleolar fossa is indistinct in contrast to well formed fossa in odontocetes (the odontocete malleus directly articulates with the petrosal in this fossa); the ossicular chain of stapes, incus, and malleus is constructed as in odontocetes with the malleus fused to the tympanic bulla via the processus gracilis.

The anterior process is well formed in mysticetes and distinctly larger than the anterior process of odontocete petrosals. Likewise, the posterior process is greatly enlarged in mysticetes as compared to the much-reduced posterior process in odontocetes. However, in both groups the posterior process is a composite structure with contributions from both the petrosal and tympanic [25]. In mysticetes the two portions of the composite posterior process fuse early in ontogeny, while in odontocetes they typically remain unfused throughout life [25].

The anterior and posterior pedicles that form the bony connections between the petrosal and tympanic bulla serve to define the anterolateral and posterior borders of the middle ear, respectively (Figure 1). The orientation of the posterior pedicle is roughly transverse and perpendicular to the parasagittally oriented anterior pedicle. The latter is formed from the embryonic accessory ossicle [27] and is firmly ossified with the ventral surface of the pars cochlearis and the anterolateral edge of the lateral wall of the tympanic bulla. In having two well-ossified pedicles joining the tympanic bulla to the petrosal the mysticete petrotympanic stands apart from the odontocete condition of only a single posterior pedicle. In odontocetes the anterior connection between the petrosal and tympanic bulla is via an intermediate accessory ossicle.

The anatomy of the mysticete inner ear as currently understood is summarized in several reports [6], [11], [15], [29]–[31]. In cetaceans generally, the vestibule and semicircular canals are reduced in volume compared to the cochlea [32]–[35], and mysticetes are no exception [36]. The size of the semicircular canals is related to rotational sensitivity [37]–[39], and it has been hypothesized that small canals in cetaceans is an adaptation to agile locomotion in a three-dimensional aquatic environment [35]. However, the bottlenose dolphin (*Tursiops truncatus*) rotates its head at slower velocities than the terrestrial artiodactyl *Bos taurus* [40], thereby suggesting that agility alone may not explain semicircular canal reduction in cetaceans. The driving force behind a reduced vestibular system in cetaceans remains unresolved. Although differences between the semicircular canals and vestibules of baleen and toothed whales have not been examined in any detail, mysticetes can be distinguished from odontocetes by a greater number of turns completed by the cochlea [31], as well as anatomy of internal cochlear structures [6], [15], [29]–[31].

**Figure 5:**
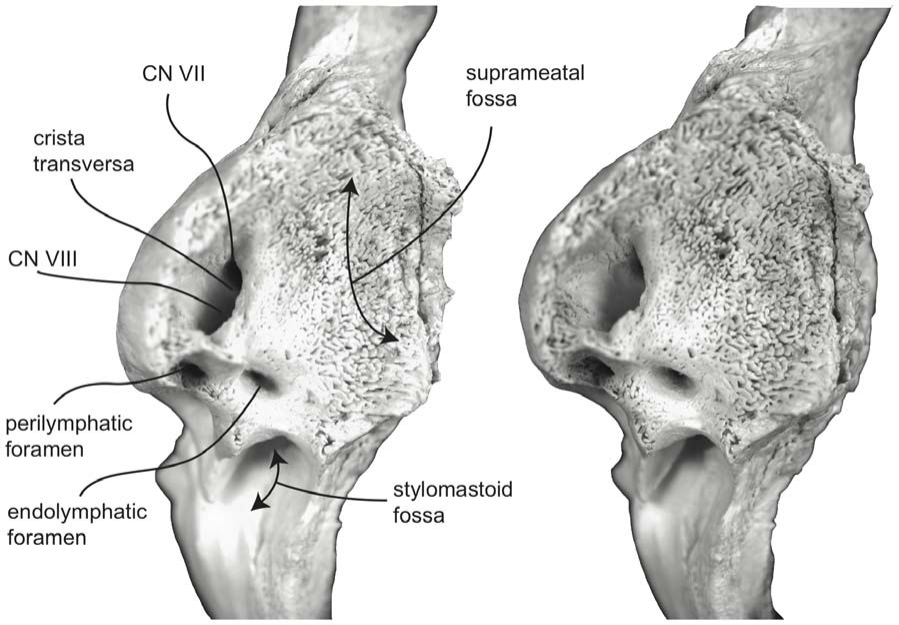
Stereophotograph of right petrosal of *Balaenoptera acutorostrata* (SDSNH 23642) in dorsomedial view.

The endocranial surface of the mysticete petrotympanic complex is characterized by a number of important features including the rough and jagged surface texture of this region, the closely appressed endolymphatic and perilymphatic foramina, and variable development of the internal auditory meatus (Figure 1, 5). The internal auditory meatus varies from a distinct common opening for both the auditory and facial nerve canal openings separated by a low, saddled crista transversa, to a condition in which both openings are distinct and separated by a prominent crista transversa that rises to the endocranial surface of the petrosal [9], [20]. The variation observed for the internal auditory meatus is discussed more fully below for individual species.

Ontogeny provides another source of petrotympanic morphologic variation that can only briefly be described here. The tympanic bullae of neonate and yearling individuals typically possess smooth and rounded external surfaces in contrast to the roughened and sharply angled surfaces of adult individuals. This textural difference is especially prominent on the involucral (dorsal) and medial surfaces of the bulla. In the former the involucral surface is smooth and lacks the strong transverse creases of adults, and in the latter the main and involucral ridges are low and poorly defined in comparison to the much more prominent ridges of adults. The petrosals of neonate and yearling individuals typically possess short anterior and posterior processes relative to the adult condition of distinctly longer anterior and posterior processes. In addition, the endocranial surface of the pars cochlearis in neonates and juvenile individuals typically is low and extremely porous rather than extended dorsally into the cranial hiatus. Further, the internal auditory meatus of neonates and juvenile individuals is broadly subdivided into distinct foramina for CN VII and CN VIII by a prominent crista transversa. In adult individuals the internal auditory meatus generally consists of a single common opening, at the bottom of which lies the two foramina separated by a deeply recessed crista transversa [20; personal observation].

### Anatomical Terminology

The anatomy of the mysticete ear has been described to various degrees in several studies [1], [4], [9], [11], [17], [26], [28], [41]. Unfortunately, the anatomical terminology used in these previous studies is inconsistent. The most recent attempt to synonymize cetacean anatomical terminology across the skulls of therian mammals was performed by Mead and Fordyce [25], but their study focused on odontocetes (specifically *Tursiops truncatus*). Although we largely accept their suggestions (additional sources of terminology were used [16], [20]), there are a few anatomical terms used in the present study that differ from those of Mead and Fordyce [25]. Most fundamental is use of the term ‘petrosal’. ‘Petrosal’ and ‘periotic’ often are used interchangeably [11], [25], [42]–[45], but using two terms to refer to the same bone can be confusing at best and misleading at worst.

‘Periotic’ literally refers to an area around the structures of the inner ear, and ‘periotic’ is used to describe such a region (often in terms of development), and not a specific bone in many studies focused on mammals [46]–[49] and other non-mammalian vertebrates [50]–[55]. Conversely, ‘petrosal’ is a descriptor, derived from the Greek word *petros* meaning stone [56], referring to the dense and stone-like nature of this bone in mammals. Mead and Fordyce recognized that the term ‘petrosal’ is synonymous with ‘periotic’ [25], but those authors chose to use ‘periotic’ in their lexicon given the term's “historically widespread [use] in cetacean literature” ([25], p. 124). Truly, cetacean literature is dominated by the use of ‘periotic’ over ‘petrosal’. However, ‘petrosal’ is used to a far greater extent in the anatomical literature on non-cetacean mammals. In order to emphasize the homology of the bones surrounding the inner ear cavities among cetaceans and all other mammals, we follow previous authors [2], [9], [11], [13] and prefer to use the term ‘petrosal’. Likewise, we refer to the complex formed by the petrosal and tympanic bulla as the ‘petrotympanic’ rather than the ‘tympano-periotic’ used in many previous cetacean studies.

A structure on the lateral side of the petrosal referred to as the superior process in many cetacean studies is homologous to the tegmen tympani of terrestrial mammals [11], which separates the lateral portion of the middle ear cavity from the cranial cavity. Indeed the structure of the ear region of cetaceans is distinctive, and Mead and Fordyce suggest that the tegmen tympani of cetaceans is so highly derived that use of the typical mammalian term can lead to confusion [25]. However, the ability to ascertain morphological transformations within the auditory region of cetaceans relies on accurate comparisons of homology among cetaceans and their terrestrial relatives. Only those features that are neomorphic within Cetacea should be given new names (as suggested by Luo and Gingerich [2]) and the number of unnecessary synonyms should be reduced. Consequently, we prefer to use the term ‘tegmen tympani’ for this structure.

A pair of openings that penetrate the promontorium, the fenestra cochleae and fenestra vestibuli, often are referred to as the fenestra rotunda (round window) and fenestra ovalis (oval window) respectively based on the shapes of the structures (not only in cetacean literature, but also in the more general mammalian literature). However, two openings that transmit branches of the trigeminal nerve, namely the foramen rotundum and the foramen ovale, are described in very similar terms. The similarity in these names can lead to confusion [57], and the shapes of the openings through the promontorium are not always distinctly ‘round’ or ‘oval’ [58]. The terms ‘fenestra rotunda’ and ‘fenestra ovalis’ are used commonly in mammalian literature, although use of ‘fenestra cochleae’ and ‘fenestra vestibuli’ is just as widespread, and less prone to confusion than the alternatives. Further, the latter terms reflect the adjacent important internal neurological structures of the ear region, namely the cochlea (organ of hearing) and vestibule, semicircular canals and utriculus and sacculus (organ of balance). Therefore, we prefer to use the terms ‘fenestra cochleae’ and ‘fenestra vestibuli.’

Lastly, many descriptions of the ear regions of whales describe a structure on the promontorium of the pars cochlearis that is identified in numerous studies as the ‘caudal tympanic process’ [11], [49], [59], [60]. However, an anteroventral projection of the pars canalicularis posterior to the promontorium and stapedial muscle fossa is referred to as the ‘caudal tympanic process’ for many non-cetacean mammals [61]–[65]. This confusion was recognized by Mead and Fordyce [25], and although they discuss alternative terminology, such as ‘posterior cochlear crest’ (following previous work by Fordyce [66]), they continue to use ‘caudal tympanic process’ for the structure on the pars cochlearis (as does O'Leary [9]). Given the widespread use of ‘caudal tympanic process’ to refer to a structure on the pars canalicularis of most non-cetacean mammals, we propose to follow the terminology of Fordyce [66] and refer to the process on the promontorium as the ‘posterior cochlear crest’.

## Results And Discussion

### Morphologic Descriptions of Extant Mysticete Petrotympanics

The following morphologic descriptions include combinations of apomorphic and pleisomorphic character states that together provide a useful characterization of the petrotympanic anatomy of individual taxa. For each taxon we describe the tympanic bulla first followed by a description of the petrosal. In addition the descriptions are constructed in a parallel format to facilitate anatomical comparisons. We have also included digitial images of tympanic bullae and petrosals for each taxon, incorporating four standard views for each element with salient features labeled. A taxonomic order overlies the description section and is based on the generally recognized clades within crown Mysticeti (i.e., Balaenidae, Neobalaenidae, Balaenopteridae, and Eschrichtiidae).

#### Balaenidae, *Balaena mysticetus*: Tympanic bulla

**Figure 6:**
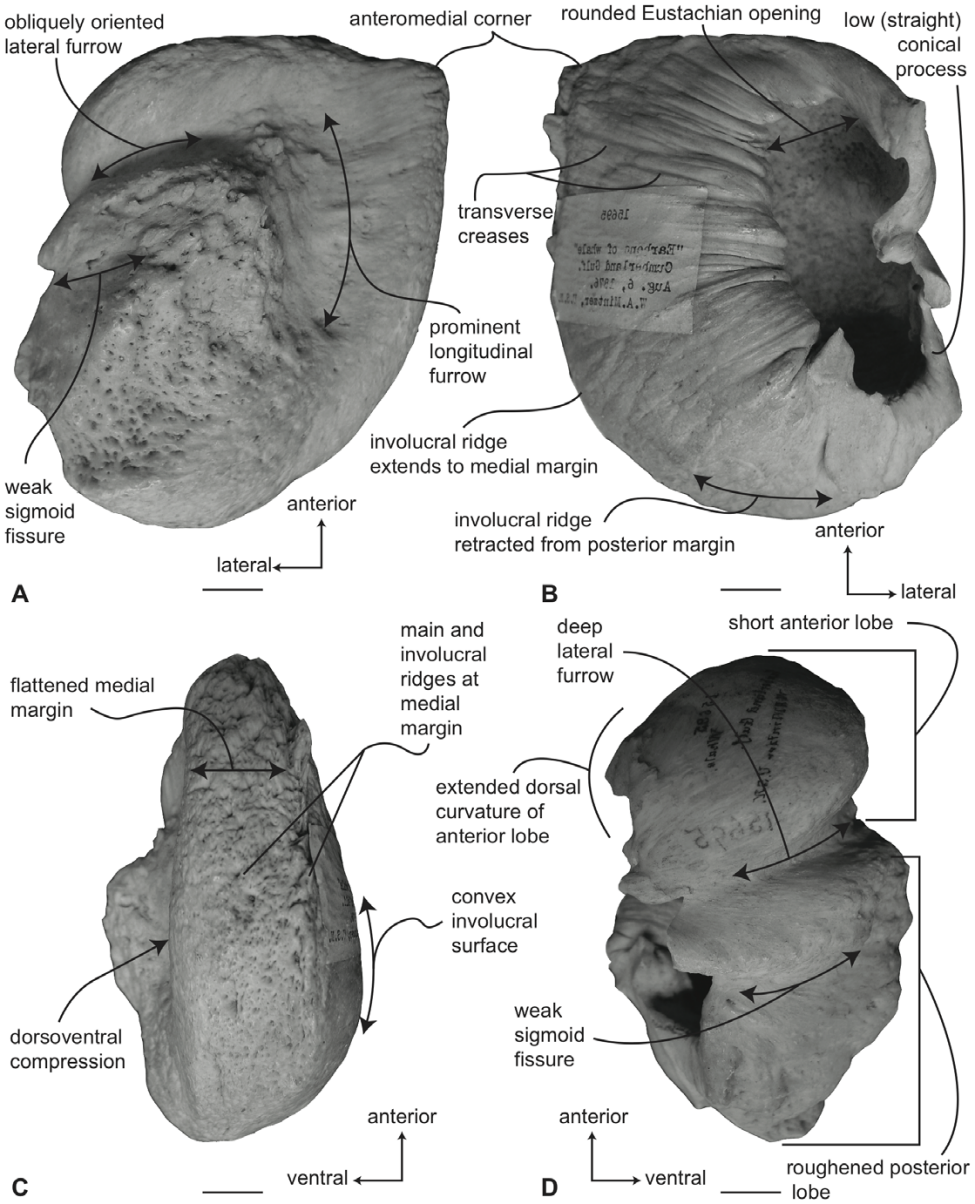
Right (reversed) tympanic bulla of *Balaena mysticetus* (USNM 15695), sigmoid process missing. A. ventral, B. dorsal, C. medial, and D. lateral views. Scale bar = 2 cm.

In ventral view (Figure 6A), the tympanic bulla of *Balaena mysticetus* is rhomboid shaped with a distinct anteromedial corner. The medial half of the ventral surface is dorsoventrally compressed to form a longitudinal furrow, a feature also seen in species of *Eubalaena*. The anterior lobe (new term) of the bulla is distinctly smaller than the posterior lobe, the two regions being separated by a deep, obliquely directed lateral furrow. Again, species of *Eubalaena* share this feature. The main ridge (sensu Oishi and Hasegawa [16]) is extended anteriorly to form a prominent, angular anteromedial corner, which is responsible for the characteristic rhomboid shape of the bulla. The sigmoid fissure is short and weakly developed and the conical process is short (see below).

In dorsal view (Figure 6B) the involucral ridge extends to the medial margin of the bulla, which is primarily formed by the prominent main ridge (i.e., USNM 15695 and ZMUC 27). Posteriorly, the involucral ridge is retracted from the posterior end of the bulla, which also is formed by the main ridge. The conical process is not extended laterally and instead is nearly straight and uniformly thin. This sharply contrasts with the balaenopteroid condition in which the conical process is elevated, arched and generally thick. The Eustachian opening is relatively small and the involucral surface is uniformly broad, convex, and heavily sculpted by transverse creases, especially adjacent to the Eustachian opening. In balaenopterids the involucral surface is generally narrower, more planar to concave in profile, and more weakly sculpted.

In medial view (Figure 6C) the main and involucral ridges parallel one another and form a flattened and anteroposteriorly elongated medial margin. In adults this elongated medial margin is extended anteriorly to form the distinct anteromedial corner of the bulla mentioned above. Also in this view the dorsoventral compression of the bulla is clearly evident on the ventral surface. Likewise, the broadly convex longitudinal profile of the involucral surface is distinct.

In lateral view (Figure 6D) the relative size difference between the smaller anterior lobe and the much larger posterior lobe is clear. Also apparent is the relative development of the deep and broad lateral furrow and the shallow and short sigmoid fissure. The posterior lobe of the bulla is characterized by a rugose surface texture. In addition, the dorsolateral margin of the anterior lobe is dorsally curled to the extent that it extends above the level of the involucral surface.

#### Balaenidae, *Balaena mysticetus*: Petrosal

**Figure 7:**
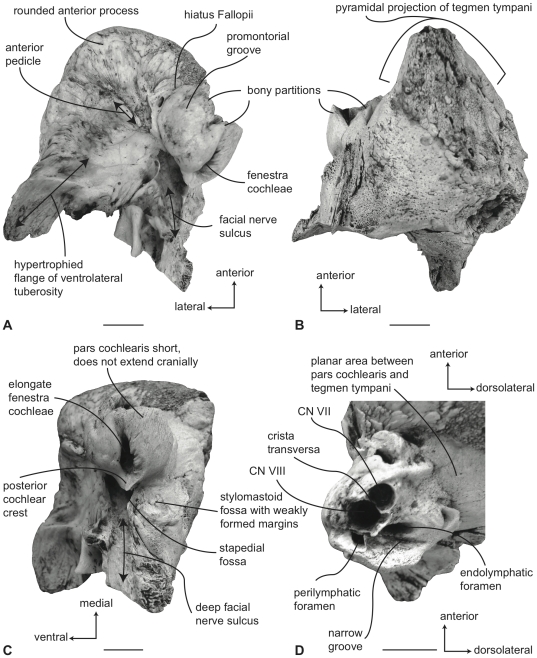
Right petrosal of *Balaena mysticetus* (LACM 97312), posterior process missing. A. ventral, B. dorsal, C. posterior, and D. dorsomedial views. Scale bar = 2 cm.

In ventral view (Figure 7A), the flange of the ventrolateral tuberosity (following Geisler and Sanders [49]) of the anterior process of the petrosal is hypertrophied and extended posterolaterally to form an acute angle with the adjacent posterior process. The body of the anterior process is anteroposteriorly short and broadly rounded at its anterior apex. The promontorium is relatively small anteroposteriorly and transversely and its ventral surface is rounded and convex. There is a distinct, but irregular promontorial groove immediately adjacent to the dorsomedial rim of the promontorium; a balaenid feature shared with many extinct mysticetes (eg. *Eomysticetus* and *Parietobalaena*, personal observation). The hiatus Fallopii opens through the ventral surface of the promontorium medial to the juncture between the promontorium and the anterior process. The groove for the tensor tympani muscle is deeply recessed along this same juncture. The epitympanic recess is broad and smooth and lacks a clearly defined malleolar fossa. The posterior cochlear crest is short and thin with a pointed tip that only slightly extends over the ovoid, relatively shallow stapedial fossa. The stapedial fossa is separated from the facial nerve sulcus by a thin longitudinal ridge. The composite posterior process is relatively broad and short in comparison to the narrower and more elongated processes of balaenopterids. The facial nerve sulcus in *Balaena* is distinct and broadly open.

In dorsal view (Figure 7B) the junction between the tegmen tympani and pars cochlearis is extended dorsally as a robust pyramidal process. The suprameatal region is not concave or convex but forms a planar saddle between the dorsal edge of the promontorium and the dorsal edge of the tegmen tympani. In species of *Eubalaena* the suprameatal region is elevated and continuous between the promontorium and tegmen tympani. The dorsolateral surface of the tegmen tympani is broadly rounded.

In posterior view the fenestra cochleae is large and recessed into the promontorium with a narrow dorsomedially directed embayment extending towards the dorsomedial rim of the promontorium (Figure 7C). The fenestra cochleae is separate from the perilymphatic foramen and is larger than the fenestra vestibuli. The facial nerve sulcus continues as a deep groove onto the posterior side of the posterior process. The posteroventral and posterodorsal margins of the stylomastoid fossa are weakly developed on the petrosal. The dorsomedial rim of the promontorium is marked by flattened to columnar bony extensions that give the rim an irregular profile.

In dorsomedial view (Figure 7D) all of the endocranial foramina are positioned within a common depression defined by the elevated dorsomedial rim of the promontorium. The large and ovoid internal auditory meatus is centrally placed and divided into a large, circular opening for CN VIII and a slightly smaller, circular internal opening for CN VII. These openings are separated by a thin crista transversa, which does not reach the endocranial surface of the petrosal. The endolymphatic foramen is elliptical and positioned posterolateral to the opening for CN VII. The smaller circular perilymphatic foramen is separated by a broad bony septum from the endolymphatic foramen (Figure 2D). The shape of these openings differs in USNM 291101 in which the endolymphatic foramen is compressed and lies further laterally than in USNM 63300. In the former specimen the perilymphatic foramen is circular and separated from the internal auditory meatus by a high, thin bony septum.

#### Balaenidae, *Eubalaena* spp.: Tympanic bulla

We provide below a composite description of the three *Eubalaena* species given our limited samples for each taxon and mixed semaphorants. We have described morphologic variation that may be species specific when more specimens are studied.

**Figure 8:**
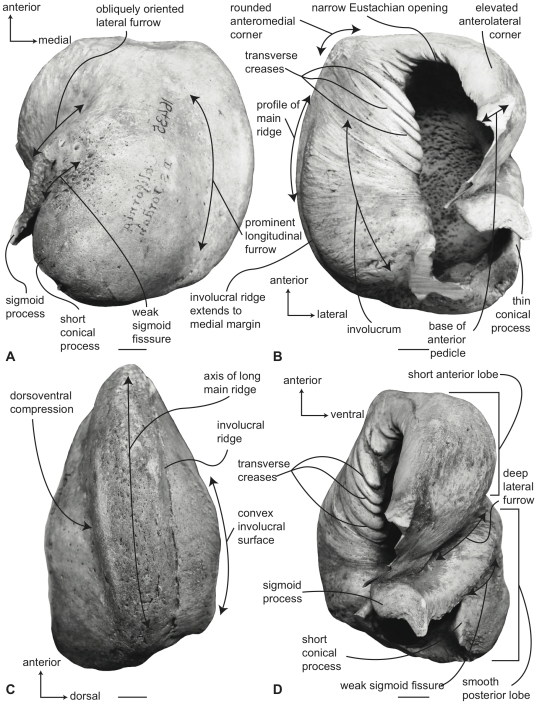
Right (reversed) tympanic bulla of *Eubalaena japonica* (USNM 16435), sigmoid process missing. A. ventral, B. dorsal, C. medial, and D. lateral views. Scale bar = 2 cm.

Specimens of tympanic bullae referred to *E. japonica* are generally larger than other species (Table S2). In ventral view (Figure 8A) the medial half of the ventral surface of the bulla is dorsoventrally compressed to form a longitudinal furrow, as in *Balaena*. In addition, the anterior lobe is smaller in the anteroposterior direction than the posterior lobe, and the lateral furrow is deeply inset and obliquely oriented. Although the main ridge is extended anteriorly, the anteromedial corner is not produced as a sharply angled corner, but instead is more broadly rounded. The effect, however, still imparts an overall rhomboid shape to the bulla. There is some variation in the degree of roundness/angularity of the anteromedial corner preserved in the specimens examined, which may in part be an ontogenetic artifact. The sigmoid fissure is weakly developed.

In dorsal view (Figure 8B) the involucral ridge varies from a raised edge to a poorly defined margin marked by a change in surface texture (i.e., smooth to roughened). The medial extent of the involucral ridge is polymorphic and in some specimens extends to the medial margin coincident with the main ridge, while in other specimens the ridge is retracted laterally from the medial margin of the bulla. Posteriorly, the involucral ridge is retracted from the posterior end of the bulla, which is thus formed by the main ridge. As in *Balaena*, the conical process is not extended laterally and instead is short, nearly straight and uniformly thin. The Eustachian opening is relatively larger than in *Balaena* and extends further medially. Consequently, the involucral surface narrows anteriorly. Transverse creases on this surface are variably developed.

In medial view (Figure 8C) the prominent main ridge is broad and dorsoventrally rounded, and tapers anteriorly. The involucral ridge roughly parallels the main ridge. The dorsoventral compression of the ventral surface also is evident in this view, as is the broadly convex profile of the involucral surface.

In lateral view (Figure 8D) the relative size differences between the anterior and posterior lobes is evident in the anteroposterior direction, as is the relative development of the deep lateral furrow and the weak sigmoid fissure. As in *Balaena*, the dorsolateral margin of the anterior lobe is dorsally curved beyond the level of the involucral surface. However, the posterior lobe lacks the rugose external surface texture observed in *Balaena*.

#### Balaenidae, *Eubalaena* spp.: Petrosal

**Figure 9:**
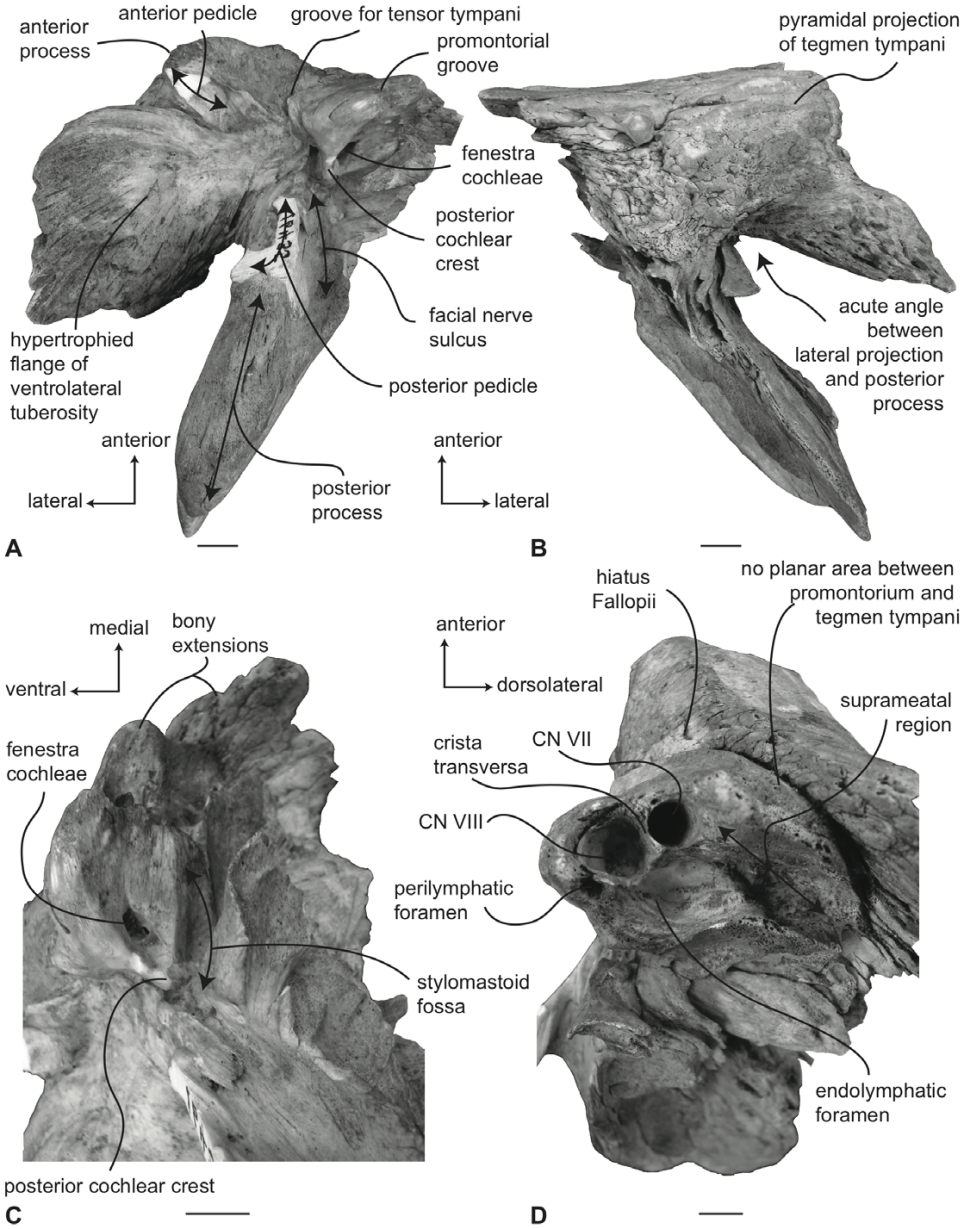
Right (reversed) petrosal of *Eubalaena japonica* (USNM 16435). A. ventral, B. dorsal, C. posterior, and D. dorsomedial views. Scale bar = 2 cm.

In ventral view (Figure 9A), the flange of the ventrolateral tuberosity of the anterior process of the petrosal is hypertrophied and extended posterolaterally to form an acute angle with the adjacent posterior process as in *Balaena*. Likewise, the body of the anterior process is anteroposteriorly short and broadly rounded at its anterior apex. The promontorium is relatively small anteroposteriorly, but is relatively longer transversely than in *Balaena mysticetus*. The ventral surface of the promontorium is rounded and convex. There is a distinct, but irregular promontorial groove more removed from the dorsomedial rim of the promontorium than in *B. mysticetus*. The hiatus Fallopii opens through the ventral surface of the promontorium medial to the juncture between the promontorium and the anterior process. The groove for the tensor tympani muscle is deeply recessed along this same juncture. The epitympanic recess is broad and smooth and lacks a clearly defined malleolar fossa. The posterior cochlear crest is thin but broader than in *B. mysticetus* and forms the floor of the large and broadly hemispherical stapedial fossa. The stapedial fossa is confluent with the facial nerve sulcus. The composite posterior process is short and thick, relative to the balaenopterid condition, and typically tapers to a pointed distal termination. The facial nerve sulcus is marked by prominent dorsal and lateral margins and extends as a relatively wide channel onto the posterior process. It is partially floored at its anterior extremity in some specimens (e.g. SAM 18071; Fig 9A).

In dorsal view (Figure 9B) the junction between the tegmen tympani and pars cochlearis is extended dorsally as a robust pyramidal process, similar to the condition in *B. mysticetus*. The suprameatal region of the pars cochlearis is narrow and elevated between the dorsal edge of the promontorium and the dorsal edge of the tegmen tympani. In *B. mysticetus* the suprameatal region is depressed between the promontorium and tegmen tympani and is developed as a narrow planar surface. The dorsolateral surface of the tegmen tympani where it contacts the squamosal is broadly swollen and hemispherical.

In posterior view (Figure 9C) the fenestra cochleae is large and roughly elliptical and is recessed into the promontorium. In some specimens the dorsal rim of the fenestra cochleae extends as a fissure towards the dorsomedial rim of the promontorium and varies from an open sulcus to a closed seam marked by a distinct suture. This probably represents an ontogenetic morphocline and although it mimics the condition in *Eschrichtius robustus*, a bony septum remains between the fenestra cochleae and the perilymphatic foramen and there is no direct connection between the two openings. The fenestra cochlea is distinctly larger than the fenestra vestibuli. The facial nerve sulcus continues as a deep groove onto the posterior side of the posterior process. The posteroventral and posterodorsal margins of the stylomastoid fossa are weakly developed on the petrosal. As in *Balaena mysticetus*, the dorsomedial rim of the promontorium is marked by flattened to columnar bony extensions that give the rim an irregular profile.

In dorsomedial view (Figure 9D) all of the endocranial foramina are positioned within a common depression defined by the elevated dorsomedial rim of the promontorium. The large and ovoid internal auditory meatus is centrally placed and divided into a large, circular opening for CN VIII and a slightly smaller, circular opening from the CN VII. These openings are separated by a moderately thick crista transversa, which typically reaches the endocranial surface of the petrosal. The endolymphatic foramen is large and varies from elliptical to circular in outline. It is closely positioned to the posterolateral corner of the opening for CN VII. The smaller circular perilymphatic foramen is separated by a broad bony septum from the endolymphatic foramen (Figure 9D).

#### Neobalaenidae, *Caperea marginata*: Tympanic bulla

**Figure 10:**
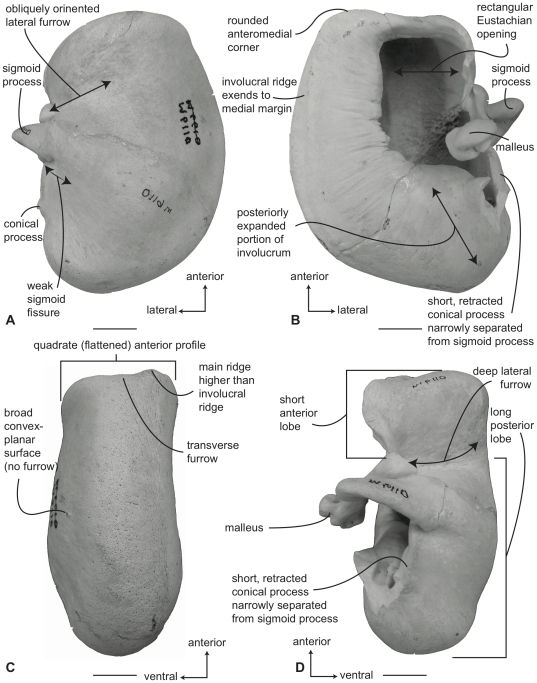
Right (reversed) tympanic bulla with malleus of *Caperea marginata* (SAM 6110). A. ventral, B. dorsal, C. medial and D. Lateral views. Scale bar  = 2 cm.

In ventral view (Figure 10A), the medial half of the tympanic bulla of *Caperea marginata* lacks the distinct dorsoventral compression seen in balaenids. However, some specimens appear to possess a slight concave flexure in this region. The anterior lobe of the bulla is relatively smaller than the posterior lobe, the two regions being separated by an obliquely directed lateral furrow. The main ridge extends anteriorly as a rounded anteromedial corner to form a generally rhomboid shaped bulla. As in balaenids, the conical process is short in height and nearly straight. The sigmoid fissure is present, but does not extend very far onto the ventral surface.

In dorsal view (Figure 10B) the involucral ridge is a poorly defined margin marked by a change in surface texture (i.e., smooth to roughened). This feature probably varies ontogenetically (i.e., adults likely possess well-defined involucral ridges). As thus defined, the ridge extends to the medial margin of the bulla, which is formed by the main ridge. Posteriorly, the involucral ridge is not retracted and extends to the posterior end of the bulla. The conical process is short and uniformly thin. The Eustachian opening is relatively large and distinctly rectangular, with a linear medial rim that meets the linear posterior rim at nearly 90°. Because of the large size of the Eustachian opening, the adjacent involucral surface is anteriorly narrow relative to the condition in *Balaena*. Delicate transverse creases cross the involucral surface, which is expanded posteriorly. Transverse creases are especially well developed along the anterior and middle portions of the involucrum.

In medial view (Figure 10C) the anterior end of the bulla has a distinctive profile produced by an anterior swelling on the ventral side and an anterior extension of the involucral and main ridge contact on the dorsal side. Thus configured, the anterior end is broadly flattened and crossed by a subtle transverse furrow, which although reminiscent of the posterior median furrow of odontocete bullae, is entirely occupied by the main ridge and not situated between the main and involucral ridges. The main ridge is broad and dorsoventrally rounded and does not taper anteriorly as in balaenids. The involucral ridge roughly parallels the main ridge with the involucral ridge terminating just short of the posterior end of the bulla. The profile of the involucral surface is planar to slightly concave.

In lateral view (Figure 10D) the bluntly terminated anterior profile is clearly evident, as is the well-developed lateral furrow. The posterior lobe lacks the rugose external surface texture observed in *Balaena*. The weak development of the conical process is also clearly seen in this view.

#### Neobalaenidae, *Caperea marginata*: Tympanic bulla

**Figure 11:**
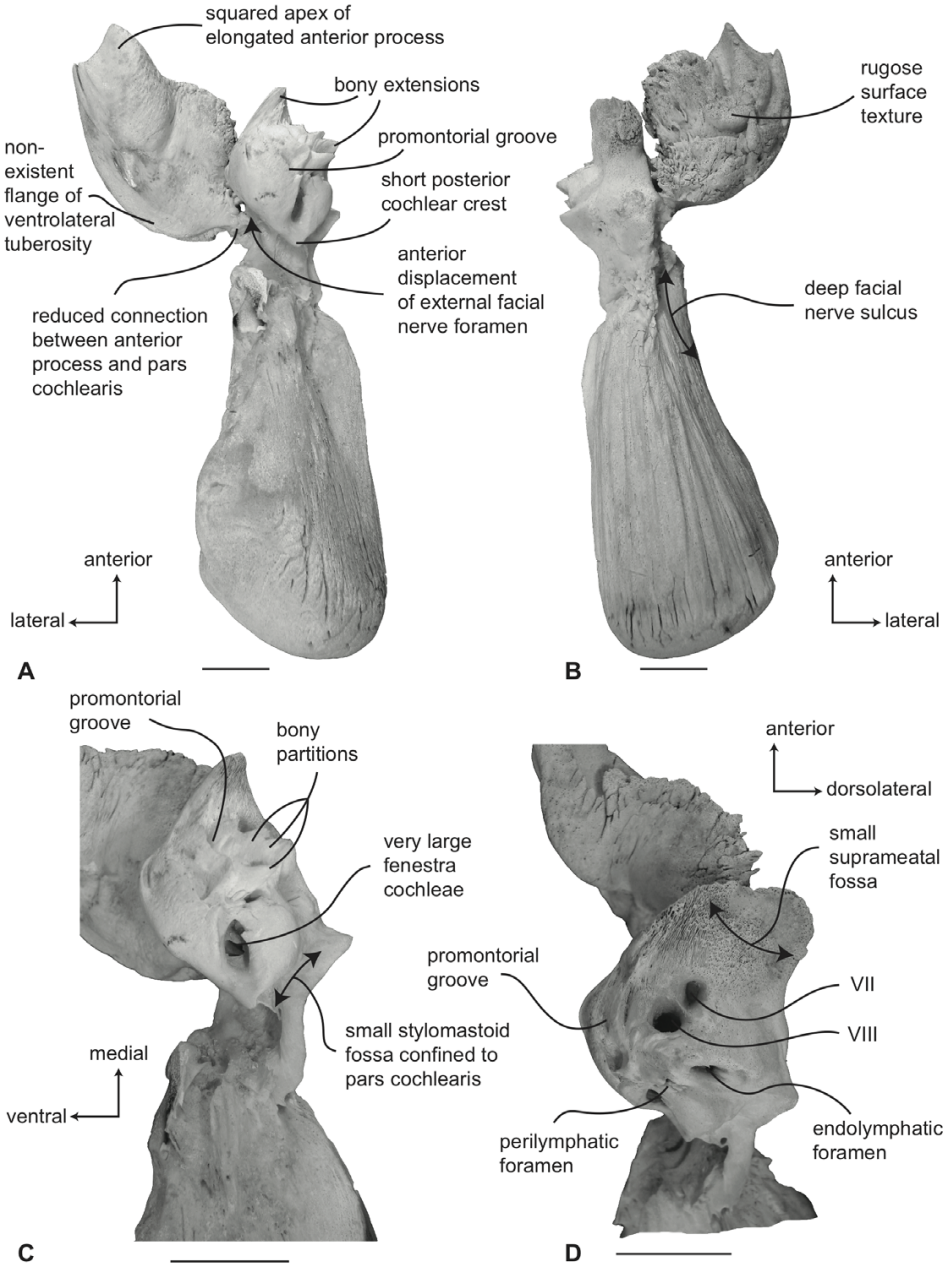
Right petrosal of *Caperea marginata* (NMV 28531). A. ventral, B. dorsal, C. posterior, and D. dorsomedial views. Scale bar = 2 cm.

In ventral view (Figure 11A), the flange of the ventrolateral tuberosity of the anterior process of the petrosal is absent and instead the area lateral to the anterior pedicle is extremely short and merely forms the rounded lateral margin of the anterior process. The body of the anterior process is anteroposteriorly long and dorsoventrally compressed relative to the condition in balaenids. Overall, the anterior process is wing-shaped with a broadly convex lateral margin, a truncated anterior margin, and a sinuous medial margin. The anterior process is only minimally attached to the pars cochlearis by a narrow bony isthmus that connects to the latter just anterior to the epitympanic recess. In dried specimens the two bony elements easily become separated. The posterior process is long and broad relative to the condition in balaenids and has a distinctly expanded distal termination developed as a “mastoid process,” reminiscent of the condition in *Eschrichtius robustus* and certain fossil mysticetes (e.g., *Herpetocetus sendaicus*, *Piscobalaena nana*, *Metopocetus durinasus*, and *Cephalotropis coronatus*). A relatively deep sulcus for CN VII extends laterally on the posteroventral side of the posterior process. The globular promontorium is relatively longer anteroposteriorly as in *Eubalaena* and unlike the shorter promontorium in *Balaena*. However, the transverse width of the promontorium more closely resembles *Balaena* than *Eubalaena*. The ventral surface of the promontorium is rounded and convex. As in balaenids there is a distinct, but irregular promontorial groove below the dorsomedial rim of the promontorium. In turn, the dorsomedial rim of the promontorium is marked by distinct columnar bony extensions. The tympanic opening for the facial nerve is not in its common and typical location adjacent to the fenestra vestibuli and at the head of the facial nerve sulcus, but instead is positioned well anteriorly near the anterior margin of the pars cochlearis and medial to the juncture between the promontorium and anterior process. The opening varies from an elongate slit with subtle sulci extending both anteriorly and posteriorly (e.g., USNM 550146) to a circular opening with more distinct anterior and posterior sulci (e.g., SAM M6110). The posterior sulci likely transmitted the hyomandibular branch of CN VII, while the greater petrosal nerve traversed the anterior sulcus. Whether the tympanic opening is homologous with the hiatus Fallopii, or if the hiatus Fallopii is absent all together in *Caperea*, remains unclear at this time. The origin of the tensor tympani muscle has no bony landmarks (i.e., there is no groove). The epitympanic region is occupied by an elliptical fossa that may accommodate the malleus. The posterior cochlear crest is short but thick and forms only the extreme anterior portion of the floor of the large and broadly hemispherical stapedial fossa. The stapedial fossa is separated from the facial nerve sulcus by a low longitudinal ridge.

In dorsal view (Figure 11B) the pars cochlearis is not extended dorsomedially as a pyramidal process as it is in balaenids, but instead is extended dorsally as a low, rounded convexity. This convexity results in a change in elevation between the surface of the tegmen tympani and the dorsolateral rim of the suprameatal region of the pars cochlearis. The suprameatal region itself is broad and roughly arcuate and its surface is depressed to form a small suprameatal fossa. The dorsal face of the anterior process has a rugose surface texture.

In posterior view (Figure 11C) the fenestra cochleae is large, hemi-circular and recessed into the surface of the promontorium. The fenestra cochleae is only slightly larger than the fenestra vestibuli. The facial nerve sulcus continues as a shallow groove onto the posterior side of the posterior process. The posteroventral and posterodorsal margins of the relatively small stylomastoid fossa are weakly developed on the petrosal. The fossa is confined to the pars cochlearis and does not extend onto the combined posterior process. As in balaenids the dorsomedial rim of the promontorium is marked by flattened to columnar bony projections that give the rim an irregular profile.

In dorsomedial view (Figure 11D) the four endocranial foramina are not clustered within a common depression as they are in balaenids. Instead the foramina are more widely spaced with a centrally placed circular opening for CN VIII that is not joined with the opening for CN VII into a common internal auditory meatus. The intervening crista transversa varies from very broad in young specimens (e.g., SAM M6110) to more narrow in older individuals (e.g., NMV C28531). The endolymphatic foramen is generally oval and well separated from the smaller, circular perilymphatic foramen.

#### Balaenopteridae, *Balaenoptera acutorostrata*: Tympanic bulla

**Figure 12:**
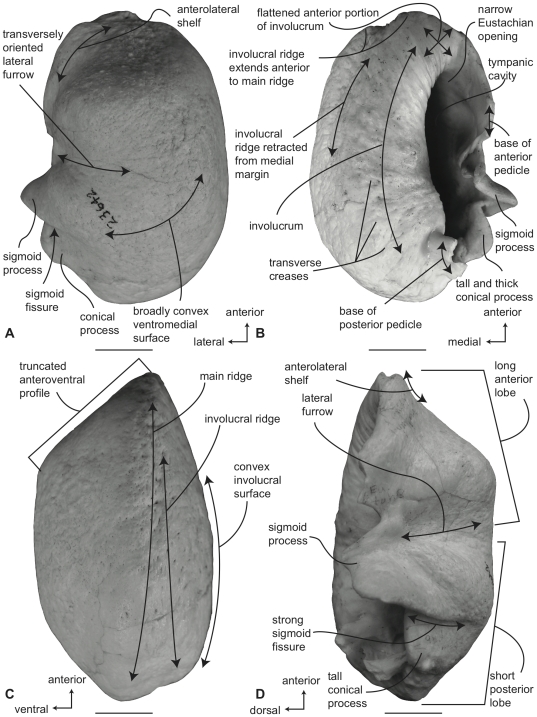
Right tympanic bulla of *Balaenoptera acutorostrata* (SDSNH 23642). A. ventral, B. dorsal, C. medial and D. lateral views. Scale bar  = 2 cm.

Compared to all other balaenopterids except *B. bonaerensis*, the tympanic bulla of *B. acutorostrata* is small (Table S3). In ventral view (Figure 12A) the medial half of the bulla is broadly convex. The anterolateral margin of the ventral surface is developed as an obliquely oriented shallow shelf or keel that begins at the juncture of the main and involucral ridges and terminates at the anterior pedicle. The anterior lobe is slightly longer than the posterior lobe (Table S4) and the lateral furrow is distinct and transversely oriented. Anteriorly, the main ridge is broadly rounded and there is no anteromedial corner. The sigmoid fissure is well developed and the sigmoid process is delicate with a thin tympanic lip. The conical process is moderately tall in height (Table S5).

In dorsal view (Figure 12B) the involucral ridge is distinct and retracted laterally from the medial margin of the bulla, which is formed by the main ridge. Posteriorly, the involucral ridge is retracted from the posterior end, while anteriorly it extends slightly beyond the termination of the main ridge. The surface of the involucrum adjacent to the Eustachian opening varies from being distinctly planar to slightly convex. Transverse creases on this surface are variably developed. The Eustachian opening is narrow and transversely compressed. The conical process is moderately tall in height, distinctly arched, and posteriorly thickened, (Table S5) with a posterior portion that is broad and shelf-like. The sigmoid process is positioned posterior to the transverse midline.

In medial view (Figure 12C) the main ridge is uniformly narrow. The main and involucral ridges roughly parallel one another for most of their length, although converging at the anterior end. The involucral ridge terminates just short of the posterior end of the bulla, but the ridge extends cranially to form the anterior border of the bone. In most specimens the anteriormost end of the bulla is pointed or slightly rounded in this view. The involucral surface is broadly convex posteriorly and lacks a distinct dorsal posterior prominence. Anteriorly, the involucral surface tends to be flattened to form a planar region medial to the Eustachian opening.

In lateral view (Figure 12D) the relative development of the broad lateral furrow and sharp sigmoid fissure is visible, as is the distinct anteroventral shelf. This shelf gives the anterior lobe of the bulla an obliquely truncated, concave profile. The posterior lobe of the bulla is relatively smooth and not rugose. The anterior lobe is not strongly curved dorsally as in balaenids. As mentioned, the dorsal surface of the involucrum adjacent to the Eustachian opening is planar.

#### Balaenopteridae, *Balaenoptera acutorostrata*: Petrosal

**Figure 13:**
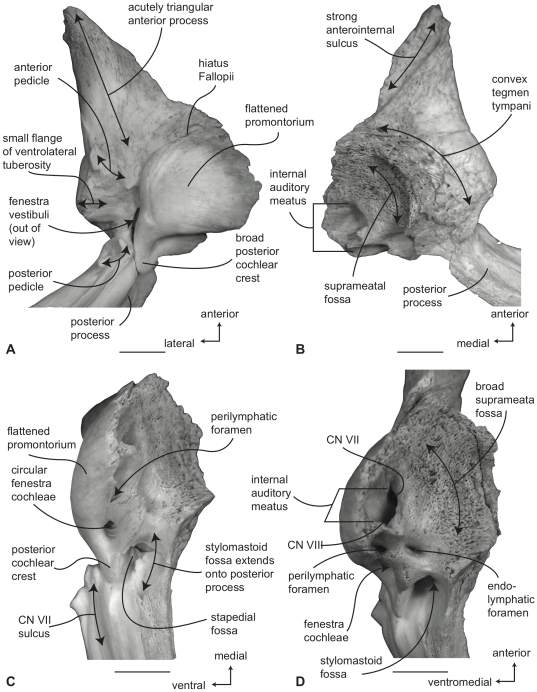
Right petrosal of *Balaenoptera acutorostrata* (SDSNH 23642). Distal end of combined posterior process not shown. A. ventral, B. dorsal, C. posterior and D. dorsomedial. Scale bar  = 2 cm.

In ventral view (Figure 13A), the flange of the ventrolateral tuberosity of the anterior process of the petrosal is small, dorsoventrally thickened, and constructed as a broad-based obtuse triangle. The body of the anterior process is anteroposteriorly long and dorsoventrally compressed. Overall, the anterior process is narrowly triangular, with an attenuated apex. The promontorium is broadly attached to the anterior process with no embayment of the anteromedial edge connecting the two as seen in *B. physalus* and *B. musculus*. However, the attachment is not as broad as in *B. bonaerensis* and *B. borealis* in which the medial margin of the anterior process is broadly convex as it rises towards the anteromedial margin of promontorium. The anteroposterior length of the anterior process is greater than that of the pars cochlearis (Table S6). The ventrally flattened promontorium is transversely elongated and relatively shorter anteroposteriorly as in all species of *Balaenoptera* except *B. musculus* and *B. physalus*. Unlike the condition in balaenids there is no promontorial groove below the dorsomedial rim of the promontorium. The tympanic opening for the facial nerve is in its typical location adjacent to the fenestra vestibuli and at the head of the distinct facial nerve sulcus. The hiatus Fallopii opens through the ventral surface of the promontorium medial to the juncture between the promontorium and the anterior process. The groove for the tensor tympani muscle is deeply recessed along this same juncture. The epitympanic recess is narrow and smooth and lacks a clearly defined malleolar fossa. The posterior cochlear crest of the petrosal is a posterodorsally oriented elongate process that contacts the tympanic portion of the posterior process to completely enclose the facial nerve sulcus. The posterior extension of the posterior cochlear crest is continuous medially with the posterior edge of the promontorium and is extended as a broad flange posterior to the fenestra cochleae. The dorsolateral surface of the posterior cochlear crest is thick and convex and protrudes into the small stapedial fossa. There is minimal separation of the stapedial fossa from the facial nerve sulcus. The composite posterior process is very long, especially in adults, with a pointed (not swollen) external mastoid apex. The body of the posterior process is compressed anteroposteriorly and expanded dorsoventrally. A relatively shallow sulcus for CN VII extends laterally on the ventral side of the posterior process.

In dorsal view (Figure 13B) the pars cochlearis is not extended dorsomedially as a pyramidal process, but instead is extended dorsally as a low, rounded convexity. This convexity results in a change in elevation between the surface of the tegmen tympani and the dorsolateral rim of the suprameatal region of the pars cochlearis. The suprameatal region itself is broad and its surface is depressed to form a distinct suprameatal fossa. The dorsal face of the anterior process has a rugose surface texture and in some specimens is crossed by a sinuous anteroexternal sulcus, similar to the groove observed in other cetaceans [24], [49], [67]. The occupant of the sulcus may be the capsuloparietal emissary vein [68] or the middle meningeal artery [24]. However, detailed dissections and descriptions of the nerves and blood vessels through the ear region of mysticetes are lacking, and until such are completed, the nerve or vessel within the sulcus is unknown.

A much broader anterointernal sulcus is present on the anteromedial edge of the anterior process and extends to the apex of the process. When the petrosal is in place within the cranial hiatus, this sulcus forms a portion of the canal for transmission of the mandibular branch of CN V as it passes over the pterygoid fossa and descends towards the dentary.

In posterior view (Figure 13C) the fenestra cochleae is teardrop shaped and recessed into the posterior face of the promontorium. Dorsally, the opening is well separated from the perilymphatic foramen, although in some specimens a closed seam marked by a distinct suture extends to the posterior rim of the perilymphatic foramen. The fenestra cochleae is distinctly larger than the fenestra vestibuli. The facial nerve sulcus continues as a shallow groove onto the posterior side of the posterior process. The stylomastoid fossa is deep and demarcated by prominent dorsal and posterior bony margins. The fossa extends only slightly onto the base of the posterior process of the petrosal. The dorsomedial rim of the promontorium lacks the flattened to columnar bony extensions seen in balaenoids.

In dorsomedial view (Figure 13D) the internal auditory meatus is a large elliptical opening immediately adjacent to the perilymphatic foramen. The openings for CN VII and VIII are deeply recessed within the internal auditory meatus. The crista transversa is variably developed as a septum and typically terminates well below the outer rim of the internal auditory meatus. Posterior to the internal auditory meatus are the elliptically- shaped perilymphatic and endolymphatic foramina. The foramina are separated from one another by a bony septum that is continuous anteriorly with the small pyramidal process. The relative size of the endolymphatic and perilymphatic foramina appears to vary from the condition with both openings subequal to the condition with the endolymphatic foramen being larger than the perilymphatic foramen.

#### Balaenopteridae, *Balaenoptera bonaerensis*: Tympanic bulla

**Figure 14:**
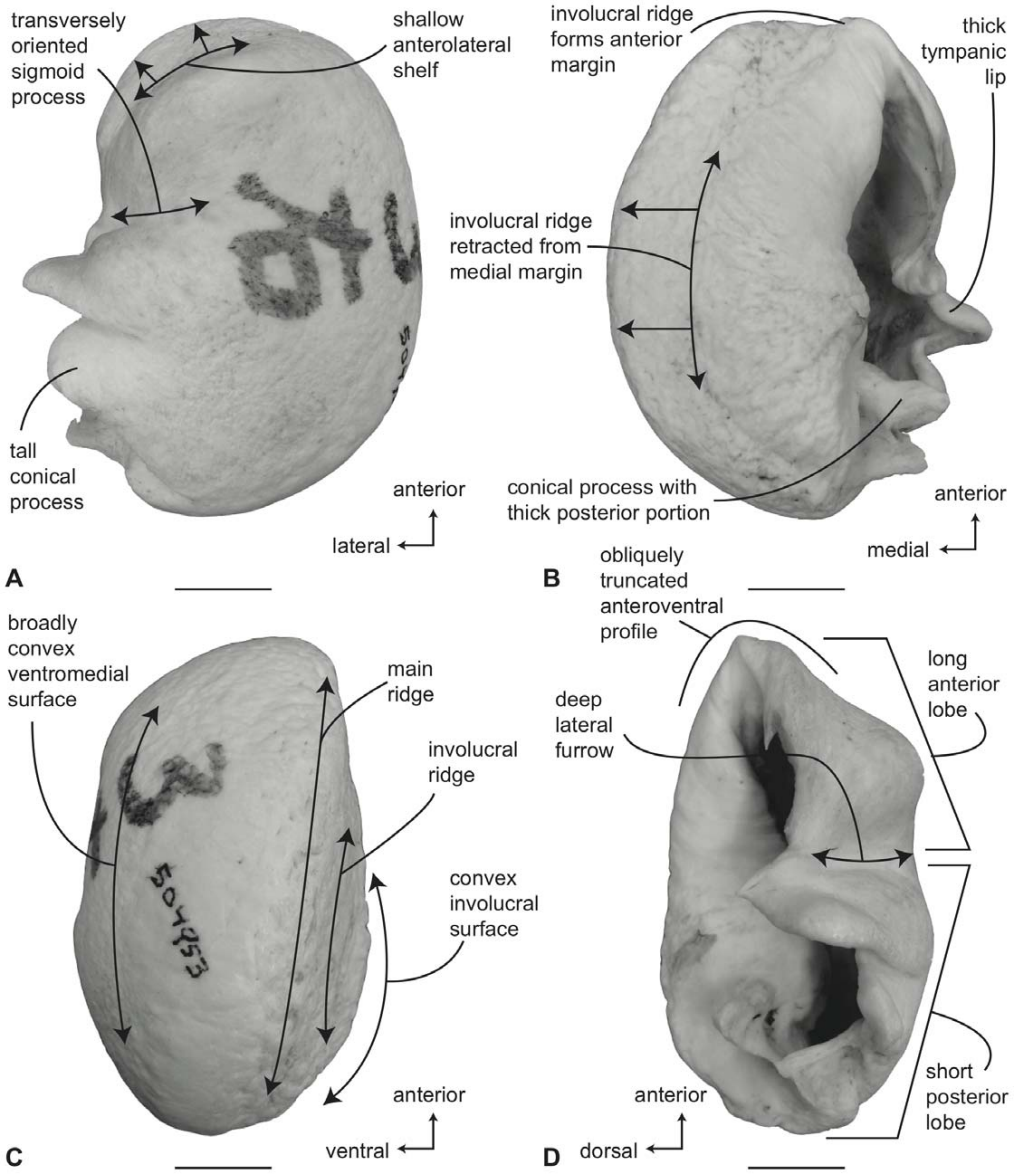
Right tympanic bulla of *Balaenoptera bonaerensis* (USNM 504953). A. ventral, B. dorsal, C. medial and D. lateral views. Scale bar  = 2 cm.

The tympanic bulla of *B. bonaerensis* is morphologically very similar to that of *B. acutorostrata*, although slightly larger (Table S3). In ventral view (Figure 14A) the medial half of the bulla is broadly convex. The anterior margin of the ventral surface is developed as an obliquely oriented shallow shelf as in *B. acutorostrata*. The anterior lobe of the bulla is slightly longer than the posterior lobe (Table S4) and the lateral furrow is distinct and transversely oriented. Anteriorly, the main ridge is broadly rounded and there is no anteromedial corner. Consequently, the overall bullar form is reniform rather than rhomboid. The sigmoid fissure is well developed and the sigmoid process is more robust than in *B. acutorostrata* with a thicker tympanic lip. The lateral furrow is relatively deep and the anteroventral shelf is well developed. The conical process is relatively tall (Table S5).

In dorsal view (Figure 14B) the involucral ridge is distinct and retracted laterally from the medial margin of the bulla, which is formed by the main ridge. Posteriorly, the involucral ridge is retracted from the posterior end, while anteriorly it extends slightly beyond the termination of the main ridge. The surface of the involucrum adjacent to the Eustachian opening is planar to slightly convex. Transverse creases on this surface are variably developed. The Eustachian opening is narrow and transversely compressed, more so than in *B. acutorostrata*. The conical process is tall in height, distinctly arched, and posteriorly thickened, (Table S5) with a posterior portion that is broad and shelf-like. The sigmoid process is positioned posterior to the transverse midline.

In medial view (Figure 14C) the main and involucral ridges are distinct and parallel one another for their entire length. The involucral ridge terminates just short of the posterior end, which is formed by the main ridge. The involucral surface is broadly convex and lacks a distinct dorsal posterior prominence.

In lateral view (Figure 14D) the relative development of the broad lateral furrow and sharp sigmoid fissure is visible, as is the distinct anteroventral shelf. This shelf gives the anterior lobe of the bulla an obliquely truncated, concave profile as in *B. acutorostrata*. The posterior lobe of the bulla is relatively smooth and not rugose. The dorsal surface of the involucrum adjacent to the Eustachian opening is planar.

#### Balaenopteridae, *Balaenoptera bonaerensis*: Petrosal

**Figure 15:**
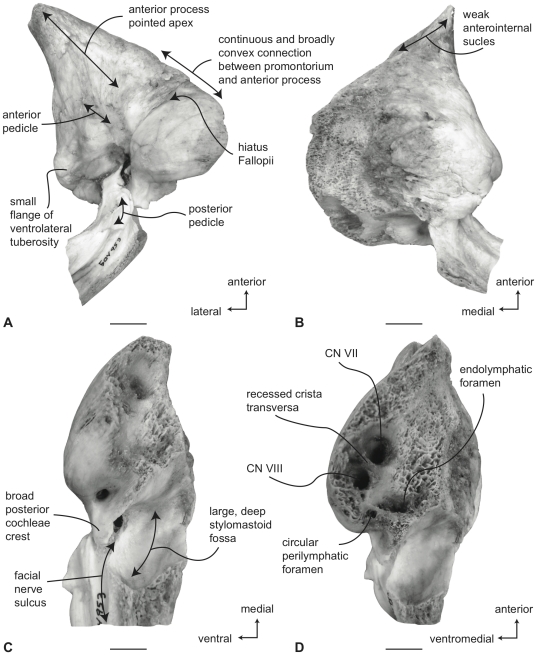
Right petrosal of *Balaenoptera bonaerensis* (USNM 504953). Distal portion of posterior process not shown. A. ventral, B. dorsal, C. posterior and D. dorsomedial views. Scale bar  = 2 cm.

In ventral view (Figure 15A), the flange of the ventrolateral tuberosity of the anterior process of the petrosa is small and dorsoventrally thick as in *B. acutorostrata* and constructed as a broad-based obtuse triangle. The body of the anterior process is anteroposteriorly long and dorsoventrally compressed. Overall, the anterior process is more broadly triangular than in *B. acutorostrata* and has an attenuated apex. The promontorium is very broadly attached to the anterior process with no embayment or flexure of the anteromedial edge. The anteroposterior length of the anterior process is greater than that of the pars cochlearis (Table S6). The ventrally flattened promontorium is transversely elongated and relatively shorter anteroposteriorly as in all species of *Balaenoptera* except *B. musculus* and *B. physalus*. There is no promontorial groove below the dorsomedial rim of the promontorium. The tympanic opening for the facial nerve is in its traditional location adjacent to the fenestra vestibuli and at the head of the distinct facial nerve sulcus. The hiatus Fallopii opens through the ventral surface of the promontorium medial to the juncture between the promontorium and the anterior process. The groove for the tensor tympani muscle is deeply recessed along this same juncture. The epitympanic recess is narrow and smooth and lacks a clearly defined malleolar fossa. The posterior cochlear crest of the petrosal is a posterodorsally oriented elongate shelf and in some specimens contacts the tympanic portion of the posterior process. The posterior extension of the posterior cochlear crest is continuous medially with the posterior edge of the promontorium and is extended as a broad flange posterior to the fenestra cochleae. The dorsolateral surface of the posterior cochlear crest is thick and convex and protrudes into the small stapedial fossa. The stapedial fossa is separated from the facial nerve sulcus by a low longitudinal ridge. The composite posterior process is very long, especially in adults. The body of the posterior process is compressed anteroposteriorly and expanded dorsoventrally. A relatively shallow sulcus for CN VII extends laterally on the ventral side of the posterior process.

In dorsal view (Figure 15B) the pars cochlearis is not extended dorsomedially as a pyramidal process, but instead forms a continuous, flattened surface with the tegmen tympani. Unlike *B. acutorostrata*, there is no suprameatal fossa and the suprameatal region itself is merely the medial portion of the flattened dorsal surface. The dorsal face of the anterior process has the typical rugose surface texture seen in other balaenopterids. The sinuous anteroexternal sulcus seen on the dorsal surface of the anterior process in some specimens of *B. acutorostrata* is not obvious on specimens of *B. bonaerensis*. However, the broad anterointernal sulcus that is present on the anteromedial edge of the anterior process in *B. acutorostrata* also occurs in most specimens of *B. bonaerensis*.

In posterior view (Figure 15C) the fenestra cochleae is roughly circular and recessed into the posterior face of the promontorium. Dorsally, the opening is well separated from the perilymphatic foramen, and there is no suture extending between the two openings. The fenestra cochleae is relatively small and equal in size to the fenestra vestibuli. The facial nerve sulcus continues as a deep groove onto the posterior side of the posterior process. The stylomastoid fossa is deep and demarcated by prominent dorsal and posterior bony margins. The fossa extends only a short distance onto the base of the posterior process of the petrosal.

In dorsomedial view (Figure 15D) the internal auditory meatus is a large elliptical opening immediately adjacent to the perilymphatic foramen. The openings for CN VII and VIII are deeply recessed within the internal auditory meatus. The crista transversa is variably developed as a septum, which never extends beyond the outer rim of the internal auditory meatus. Posterior to the internal auditory meatus are the circular perilymphatic and elliptical endolymphatic foramina. The foramina are separated from one another by a bony septum that is continuous anteriorly with the small pyramidal process. The relative size of the endolymphatic and perilymphatic foramina varies from the condition with both openings subequal to the condition with the endolymphatic foramen larger than the perilymphatic foramen.

#### Balaenopteridae, *Balaenoptera borealis*: Tympanic bulla

**Figure 16:**
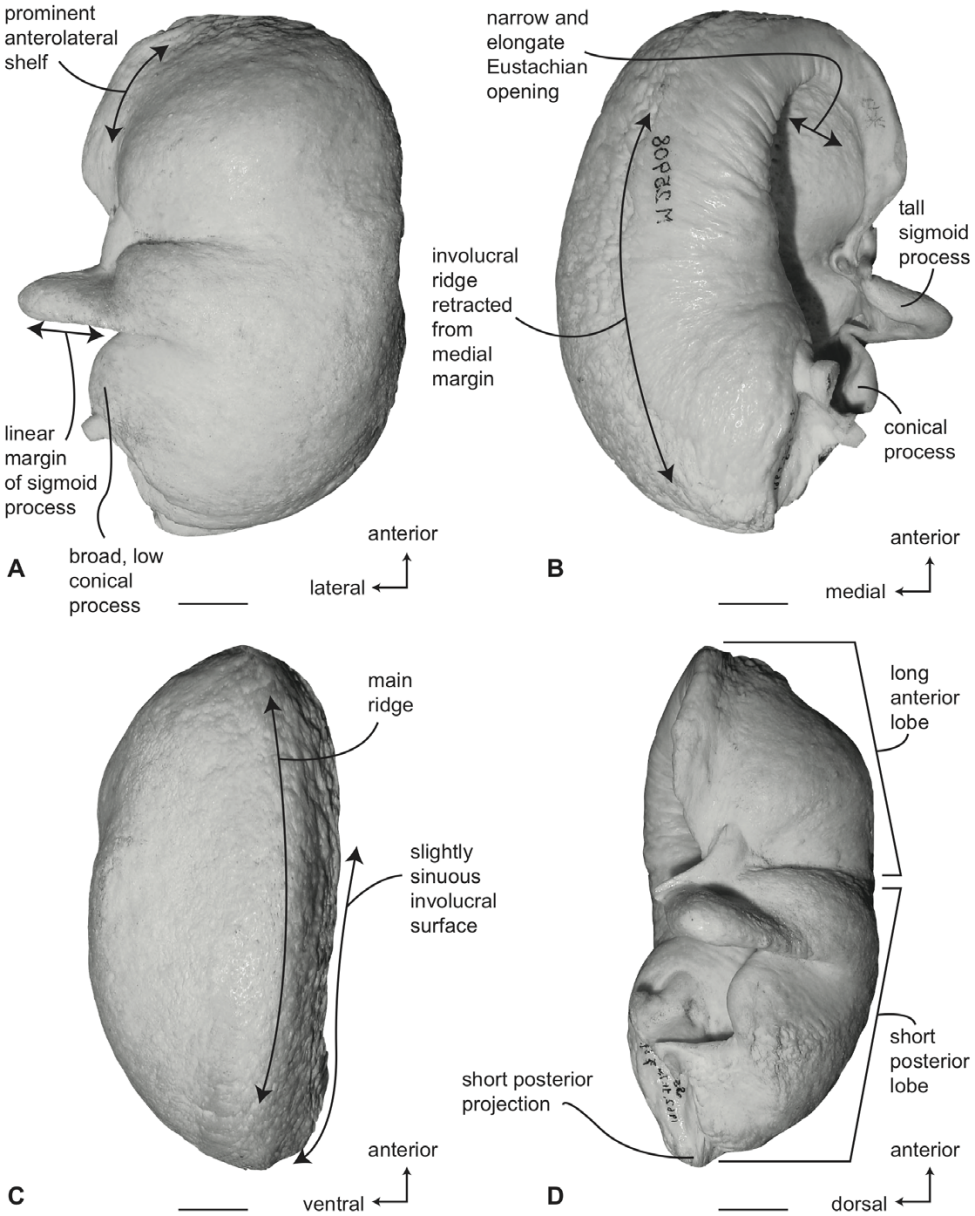
Left (reversed) tympanic bulla of *Balaenoptera borealis* (NSMT 25908). A. ventral, B. dorsal, C. medial and D. lateral views. Scale bar  = 2 cm.

The tympanic bulla of *B. borealis* is intermediate is size between the smaller bulla of *B. acutorostrata* and the larger bulla of *B. musculus* (Table S3). In ventral view (Figure 16A) the medial half of the bulla is broadly convex, with no flattening or depression of the region immediately adjacent to the main ridge. The anterolateral margin of the ventral surface is developed as an obliquely oriented prominent shelf that begins at the juncture of the main and involucral ridges and terminates at the anterior pedicle. In most specimens this shelf exhibits a noticeable keel adjacent to the anterior pedicle. The anterior lobe is slightly longer than the posterior lobe (Table S4) and the lateral furrow is distinct and transversely oriented. The sigmoid process has a nearly linear posterior margin with a thickened tympanic lip. The conical process is relatively short in height and broadly arched (Table S5).

In dorsal view (Figure 16B) the involucral ridge is distinct and retracted laterally from the medial margin of the bulla, which is formed by the main ridge. The main ridge extends posteriorly as a short projection. Posteriorly, the involucral ridge is retracted from the posterior end, while anteriorly it extends with the main ridge to form the anterior margin. The surface of the involucrum adjacent to the Eustachian opening is slightly convex. Transverse creases on this surface are variably developed. The Eustachian opening is ovoid and intermediate in relative size between *B. acutorostrata* and *Megaptera novaeangliae.* The conical process is posteriorly thickened, (Table S5) with a broad and shelf-like posterior portion. The sigmoid process is positioned posterior to the midline.

In medial view (Figure 16C) the main and involucral ridges parallel one for their entire length. In several specimens (i.e. USNM 504244 and USNM 504699) the main ridge has an elevated medial margin that flattens posteriorly similar to the condition in *Balaenoptera physalus*. In most specimens the involucral surface is shallowly depressed near the posterior end of the bulla, producing a slightly sinuous margin. The dorsal posterior prominence is weakly developed.

In lateral view (Figure 16D) the relative development of the broad lateral furrow and sharp sigmoid fissure is visible, as is the distinct anteroventral shelf. This shelf gives the anterior lobe of the bulla an obliquely truncated, concave profile as in *B. acutorostrata* and *B. bonaerensis*. The posterior lobe of the bulla is relatively smooth and not rugose. At the posterior end of the bulla the main ridge extends as a short projection.

#### Balaenopteridae, *Balaenoptera borealis*: Petrosal

**Figure 17:**
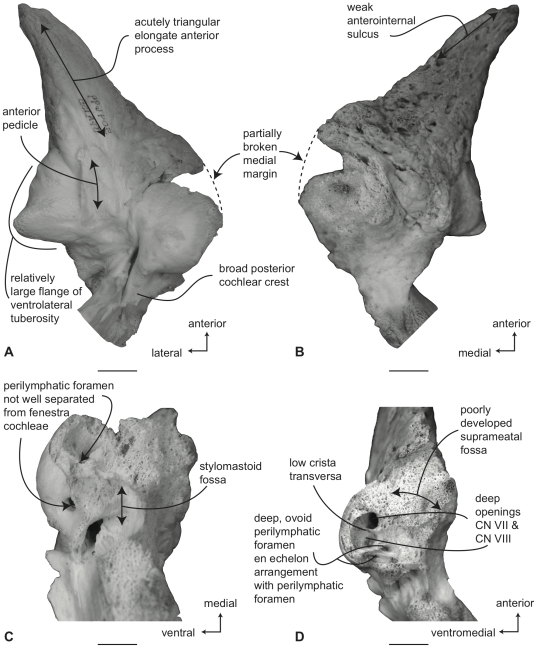
Right petrosal of *Balaenoptera borealis* (USNM 504699). Distal portion of posterior process not shown. A. ventral, B. dorsal, C. posterior and D. dorsomedial views. Scale bar  = 2 cm.

In ventral view (Figure 17A) the flange of the ventrolateral tuberosity of the anterior process of the petrosal is larger than in *B. acutorostrata* and more acutely triangular. The body of the anterior process is anteroposteriorly elongated and dorsoventrally compressed. Overall, the anterior process is triangular with an attenuated apex. The promontorium is very broadly attached to the anterior process with no embayment of the anteromedial edge. The medial margin of the anterior process is broadly convex as it rises toward the anteromedial margin of promontorium. In younger individuals this area of the petrosal is easily broken. The anteroposterior length of the anterior process is greater than that of the pars cochlearis (Table S6). The promontorium is ventrally flattened as in all species of *Balaenoptera* except *B. musculus* and *B. physalus*. The transverse diameter of the promontorium is greater than the anteroposterior diameter. This difference is even greater with *in situ* petrosals where the more poorly ossified dorsomedial extension of the ventral face of the pars cochlearis is preserved and extended well into the cranial hiatus. There is no promontorial groove below the dorsomedial rim of the promontorium. The tympanic opening for the facial nerve is in its traditional location adjacent to the fenestra vestibuli and at the head of the distinct facial nerve sulcus. Unlike most species of *Balaenoptera* the hiatus Fallopii opens through the ventral surface of the anterior process lateral to the juncture between the promontorium and the anterior process. The groove for the tensor tympani muscle is recessed along this same juncture. The epitympanic recess is broad and smooth and lacks a clearly defined malleolar fossa. The posterior cochlear crest of the petrosal is a posteriorly oriented elongate process that does not contact the tympanic portion of the posterior process. The posterior extension of the posterior cochlear crest is continuous medially with the posterior edge of the promontorium and is extended as a broad flange posterior to the fenestra cochleae. The dorsolateral surface of the posterior cochlear crest is thin and concave and forms the floor of the relatively large and hemispherical stapedial fossa. The stapedial fossa is confluent with the facial nerve sulcus and not separated from it by a low longitudinal ridge. The composite posterior process is very long, especially in adults. The body of the posterior process is compressed anteroposteriorly and expanded dorsoventrally. A relatively shallow sulcus for CN VII extends laterally on the ventral side of the posterior process.

In dorsal view (Figure 17B) the pars cochlearis is not extended dorsomedially as a pyramidal process, but instead forms a continuous, flattened surface with the tegmen tympani. Consequently, there is no suprameatal fossa and the suprameatal region itself is merely the medial portion of the flattened dorsal surface. The dorsal face of the anterior process has the typical rugose surface texture seen in other balaenopterids. In some specimens of *B. borealis* the dorsomedial corner of the anterior process is extended dorsally in concert with the dorsally extended promontorium. The sinuous anteroexternal sulcus seen on the dorsal surface of the anterior process in some specimens of *B. acutorostrata* is not obvious on specimens of *B. bonaerensis*. A weakly developed marginal anteroexternal sulcus for transmission of the mandibular branch of CN V is variably present on the anteromedial edge of the anterior process as in *B. acutorostrata* and most specimens of *B. bonaerensis*.

In posterior view (Figure 17C) the fenestra cochleae is relatively small, roughly circular, and slightly recessed into the posterior face of the promontorium. Dorsally, the fenestra cochleae is variably separated from the perilymphatic foramen; a deeply retracted dorsal margin occurs in some specimens (e.g., USNM 504701), while in others the dorsal margin forms a tall septum (e.g., USNM 571436). In most specimens a suture extends from the dorsal rim of the fenestra cochleae to the posterior rim of the promontorium. The fenestra cochleae and fenestra vestibuli are nearly equal in size. The facial nerve sulcus continues as a shallow groove onto the posterior side of the posterior process. The stylomastoid fossa is weakly developed and demarcated by weak dorsal and ventral posterior bony margins. The fossa is short and barely extends onto the base of the posterior process of the petrosal.

In dorsomedial view (Figure 17D) the internal auditory meatus is a large, elliptical opening containing similarly sized deep openings for CN VII and VIII. The crista transversa is thin and low and does not extend beyond the rim of the internal auditory meatus. Immediately posterior to the internal auditory meatus are the elliptically shaped perilymphatic and endolymphatic foramina. The foramina are generally of equal size and closely overlap one another in an *en echelon* arrangement separated by a mediolaterally oriented thin bony septum. In some specimens, the two foramina share a common basin.

#### Balaenopteridae, *Balaenoptera edeni*: Tympanic bulla

**Figure 18:**
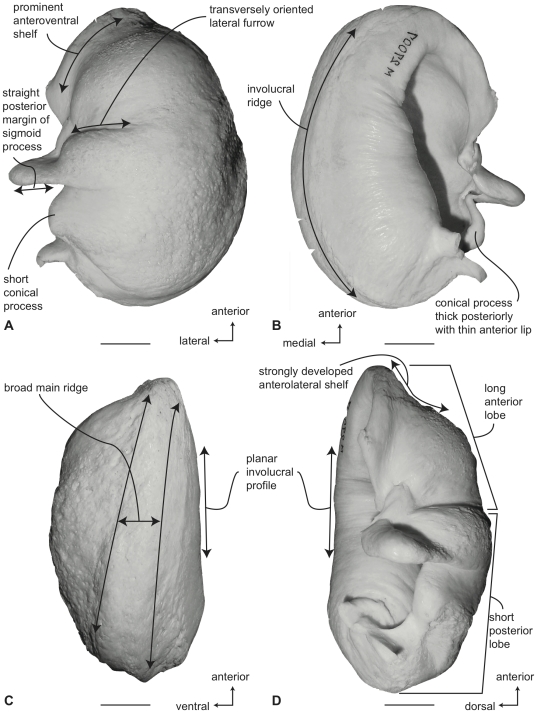
Right tympanic bulla of *Balaenoptera edeni* (NSMT 27007). A. ventral, B. dorsal, C. medial and D. lateral views. Scale bar  = 2 cm.

The tympanic bulla of *B. edeni* is similar in many features to that of *B. borealis* but is slightly smaller (Table S3). In ventral view (Figure 18A) the medial half of the bulla is slightly depressed immediately adjacent to the main ridge. This depression of the ventral surface is strongest posteriorly. The anterolateral margin of the ventral surface is developed as a prominent obliquely oriented shelf that begins at the juncture of the main and involucral ridges and terminates at the anterior pedicle. The anterior lobe is slightly shorter than the posterior lobe (Table S4) and the lateral furrow is distinct and transversely oriented. The sigmoid process has a nearly straight posterior margin with a thickened tympanic lip. The conical process is short in height and broadly arched (Table S5).

In dorsal view (Figure 18B) the involucral ridge is distinct and retracted laterally from the medial margin of the bulla, which is formed by the main ridge. The main ridge forms the anterior end of the bulla, while the posterior end of the bulla is formed by both the main and involucral ridges. There is no short posterior projection of the main ridge as seen in *B. borealis*. The surface of the involucrum adjacent to the Eustachian opening is planar to slightly convex. Transverse creases on this surface are variably developed. The Eustachian opening is ovoid and similar in size to that in *B. borealis.* The conical process is posteriorly thickened, (Table S5) with a posterior portion that is broad and shelf-like. The sigmoid process is positioned posterior to the transverse midline.

In medial view (Figure 18C), the main ridge is broad, especially posteriorly, and parallels the involucral ridge for most of its length, although converging with the latter at the anterior end. The involucral surface is planar and lacks a distinct dorsal posterior prominence.

In lateral view (Figure 18D) the strongly developed anterolateral shelf is clearly visible on the ventral surface and accentuates the obliquely truncated profile of the anterior lobe. The posterior lobe of the bulla is relatively smooth and not rugose.

#### Balaenopteridae, *Balaenoptera edeni*: Petrosal

**Figure 19:**
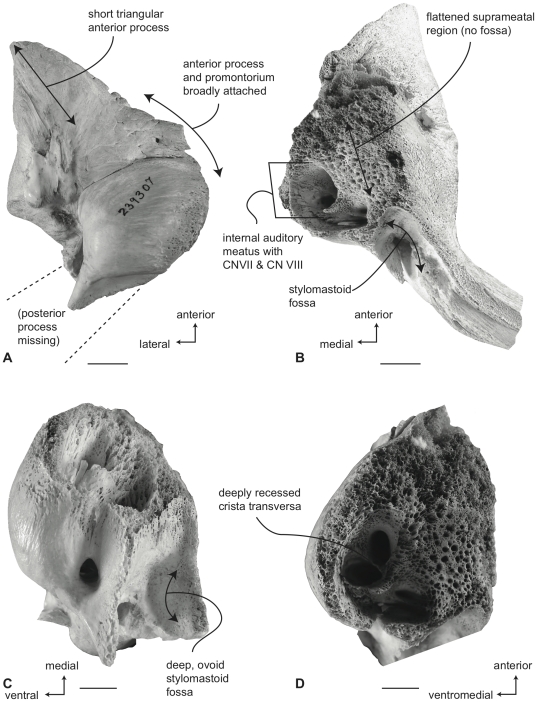
Right petrosal of *Balaenoptera edeni* (USNM 239307). Distal portion of posterior process not shown. A. ventral, B. dorsal, C. posterior and D. dorsomedial. Scale bar  = 2 cm.

In ventral view (Figure 19A), the flange of the ventrolateral tuberosity of the anterior process of the petrosal is broad and forms a dorsoventrally thickened obtuse triangle. The body of the anterior process is relatively short and dorsoventrally compressed. Overall, the anterior process is distinctly triangular, but not attenuated as in *B. borealis*. The promontorium and anterior process are firmly attached to one another, with a distinctly convex and continuous medial margin. The anteroposterior length of anterior process is greater than that of the pars cochlearis (Table S6). The surface of the promontorium is more convex than in *B. borealis*, but not globular as in *B. musculus* and *B. physalus*. The transverse diameter of the promontorium is greater than the anteroposterior diameter. There is no promontorial groove below the dorsomedial rim of the promontorium. The hiatus Fallopii is positioned lateral to the juncture between the anterior process and the promontorium. The epitympanic recess is broad and smooth and lacks a clearly defined malleolar fossa. The posterior cochlear crest is a posteriorly oriented elongate process that nearly contacts the tympanic portion of the posterior process. The posterior cochlear crest is shelf-like with a relatively straight margin that contacts the tympanic portion of the posterior process and forms a roof over the secondary facial sulcus. The stapedial fossa is relatively large, excavated into the posterior cochlear crest, and confluent with the facial nerve sulcus. The body of the composite posterior process is compressed anteroposteriorly and expanded dorsoventrally. A relatively shallow sulcus for CN VII extends laterally on the ventral side of the posterior process.

In dorsal view (Figure 19B) the pars cochlearis is not extended dorsomedially as a pyramidal process, but instead forms a continuous, flattened surface with the tegmen tympani. Consequently, there is no suprameatal fossa and the suprameatal region itself is merely the medial portion of the flattened dorsal surface of the tegmen tympani. The dorsal face of the anterior process is not as rugose as seen in other balaenopterids. There is no trace of the anterointernal sulcus that is variably present on the anteromedial edge of the anterior process as in *B. acutorostrata* and most specimens of *B. bonaerensis*.

In posterior view (Figure 19C) the fenestra cochleae is moderately large, teardrop shaped, and slightly recessed into the posterior face of the promontorium. Dorsally, the fenestra cochleae is well separated from the perilymphatic foramen, with a suture extending from the dorsal rim of the fenestra cochleae to the posterior rim of the promontorium. The fenestra cochleae and fenestra vestibuli are nearly equal in size. The facial nerve sulcus continues as a shallow groove onto the posterior side of the posterior process. The stylomastoid fossa is a relatively deep ovoid fossa that is prolonged as a narrow channel dorsomedially a short distance onto the posterior process.

In dorsomedial view (Figure 19D) the internal auditory meatus containing the facial nerve and CN VIII is an irregular single opening. The crista transversa is developed as a deeply recessed septum dividing the cranial nerve openings. The exit for the CN VII is nearly equal in size to the opening for CN VIII. The internal opening of the facial nerve canal is ovoid. The small narrowly compressed, deep perilymphatic foramen is separated by a thin bony strut from the slightly larger slit-like endolymphatic foramen. The two foramina are positioned in an *en echelon* arrangement. The septum between the internal auditory meatus and the perilymphatic foramen is also very thin.

#### Balaenopteridae, *Balaenoptera musculus*: Tympanic bulla

**Figure 20:**
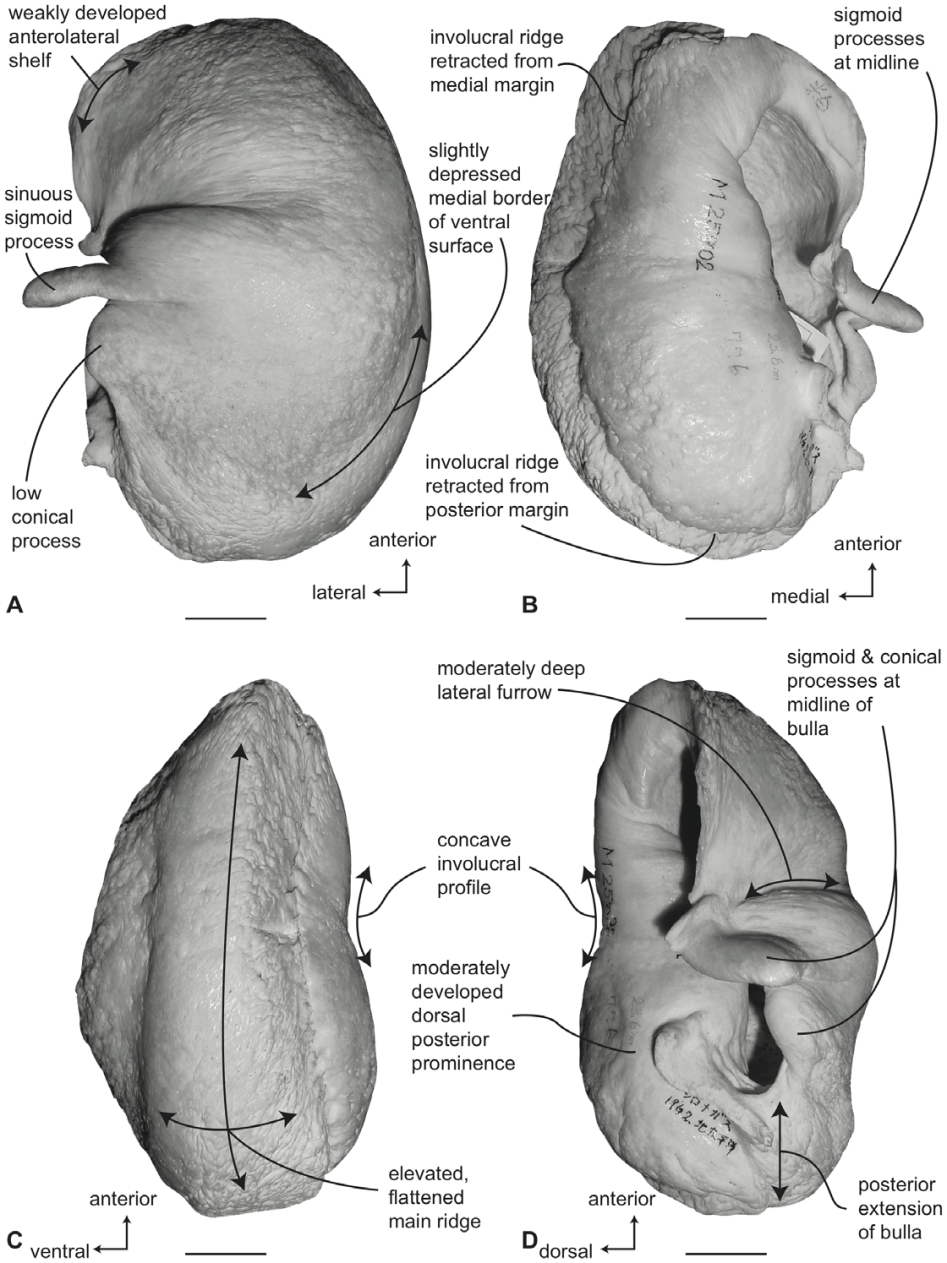
Right tympanic bulla of *Balaenoptera musculus* (NSMT 25902). A. ventral, B. dorsal, C. medial and D. lateral views. Scale bar  = 2 cm.

The tympanic bulla of *B. musculus* is distinguished from other balaenopterids by its large size (Table S3). In ventral view (Figure 20A) the posteromedial half of the ventral side is slightly depressed posteriorly, but not as deeply as in balaenids. The anterior lobe is slightly shorter than the posterior lobe (Table S4), the length of which is increased by a longitudinal thickening of the region posterior to the tympanic cavity. A moderately deep lateral furrow is present and transversely directed. The surface of the anterior lobe is rounded and convex with a weakly developed anterolateral shelf. The sigmoid process has a slightly sinuous posterior margin with a thickened tympanic lip. The conical process is relatively short in height (Table S5) and broadly arched.

In dorsal view (Figure 20B) the involucral ridge is distinct and retracted laterally from the medial margin of the bulla, which is formed by the main ridge. The main ridge forms both the anterior and posterior ends of the bulla, but is not extended as a short posterior projection as in *B. borealis*. Posteriorly, the involucral ridge is retracted from the posterior end. The surface of the involucrum adjacent to the Eustachian opening is slightly convex, while posteriorly there is a moderately developed dorsal posterior prominence. Transverse creases on this surface are poorly developed. The Eustachian opening is ovoid and similar in size to that in *B. borealis.* The conical process is thicker posteriorly than anteriorly, but not to the extent seen in other species of *Balaenoptera* (Table S5). The sigmoid process is positioned at the transverse midline, a condition unique to this species.

In medial view (Figure 20C), the main ridge is dorsoventrally broad and parallels the involucral ridge for its entire length. The main ridge is thickest posteriorly and in some specimens is developed as an elevated and flattened plateau similar to that in *B. physalus*. The involucral surface is markedly sinuous and strongly concave at its midpoint, due in part to the dorsal posterior prominence.

In lateral view (Figure 20D) the anterior lobe lacks the obliquely truncated profile of other balaenopterids, a condition accentuated by the weakly developed anterolateral shelf. The posterior lobe of the bulla is relatively smooth and not rugose.

#### Balaenopteridae, *Balaenoptera musculus*: Petrosal

**Figure 21:**
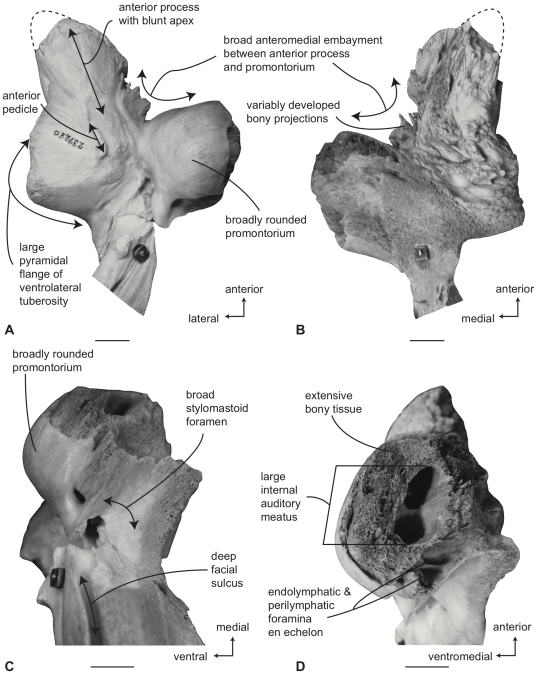
Right petrosal of *Balaenoptera musculus* (USNM 239280). A. ventral, B. dorsal, C. posterior and D. dorsomedial. Scale bar  = 2 cm.

In ventral view (Figure 21A) the flange of the ventrolateral tuberosity is relatively large, dorsoventrally thickened, and broadly pyramidal. The anterior process forms a narrowly triangular, finger-like projection that is well separated from the promontorium by an anteromedial embayment. This condition is also seen in *B. physalus* among extant balaenopterids. In other balaenopterids the anterior process is broadly attached to the promontorium, in most cases ascending to the dorsomedial rim of the promontorium as a convex medial ridge. The medial margin varies from a broadly convex edge to one marked by irregular medially directed bony extensions, not unlike those seen in *B. omurai*. These structures may be age related, apparently occurring only in older individuals. The anteroposterior length of the anterior process is greater than that of the pars cochlearis (Table S6). The promontorium is globular as in *B. physalus* and unlike the more flattened promontoria of other species of *Balaenoptera*. The transverse diameter of the promontorium is slightly greater than the anteroposterior diameter, although with *in situ* petrosals with preserved dorsomedial extensions, the difference is much greater. The hiatus Fallopii opens through the ventral surface of the promontorium medial to the juncture between the promontorium and the anterior process. The groove for the tensor tympani muscle is indistinct. The epitympanic recess is narrow and smooth and lacks a clearly defined malleolar fossa. The posterior cochlear crest of the petrosal is a posteriorly oriented elongate process that does not contact the tympanic portion of the posterior process. The posterior extension of the posterior cochlear crest is continuous medially with the posterior edge of the promontorium and is extended as a narrow (not broad) flange posterior to the fenestra cochleae. The dorsolateral surface of the posterior cochlear crest is thin and concave and forms the floor of the relatively large and hemispherical stapedial fossa. The composite posterior process is very long, especially in adults. The body of the posterior process is compressed anteroposteriorly and expanded dorsoventrally. A relatively deep sulcus for CN VII extends laterally onto the ventral side of the posterior process and in some specimens is nearly completely enclosed by a bony roof.

In dorsal view (Figure 21B) the pars cochlearis is not extended dorsomedially as a pyramidal process, but instead forms a continuous, flattened surface with the tegmen tympani. There is no suprameatal fossa and the suprameatal region is merely the medial portion of the flattened dorsal surface. The dorsal face of the anterior process has the typical rugose surface texture seen in other balaenopterids. The sinuous anteroexternal sulcus seen on the dorsal surface of the anterior process in some specimens of *B. acutorostrata* is not obvious on specimens of *B. musculus*. The anterointernal sulcus is absent from the anteromedial edge of the anterior process.

In posterior view (Figure 21C) the fenestra cochleae is relatively small, teardrop shaped, and only slightly recessed into the posterior face of the promontorium. Dorsally, the fenestra cochleae is well separated from the perilymphatic foramen. In some specimens a suture extends from the dorsal rim of the fenestra cochlea to the posterior rim of the promontorium. The fenestra cochleae and fenestra vestibuli are nearly equal in size. The facial nerve sulcus continues as a shallow groove onto the posterior side of the posterior process. The stylomastoid fossa is broad and deep and demarcated by prominent dorsal and ventral posterior bony margins. The fossa is relatively short and barely extends onto the base of the posterior process of the petrosal. With petrosals still in place in the cranial hiatus, there is a dorsomedial extension of the pars cochlearis that is composed of less densely ossified bone than the promontorium.

In dorsomedial view (Figure 21D) the internal auditory meatus is a large, elliptical opening containing large openings for CN VII and CN VIII. In some specimens the opening for CNVII is more elliptical than the opening for CN VIII. The crista transversa in adult specimens is thin and low and does not extend beyond the rim of the internal auditory meatus. Immediately posterior to the internal auditory meatus are the elliptically shaped perilymphatic and endolymphatic foramina. The foramina are generally of equally large size and closely overlap one another in an *en echelon* arrangement separated by a thin, mediolaterally oriented bony septum. In this view the sharply truncated dorsomedial rim of the promontorium is clearly evident with a rim of cancellous bone marking the area where the dorsomedial bony extension of the pars cochlearis has been broken away.

#### Balaenopteridae, *Balaenoptera omurai*: Tympanic bulla

**Figure 22:**
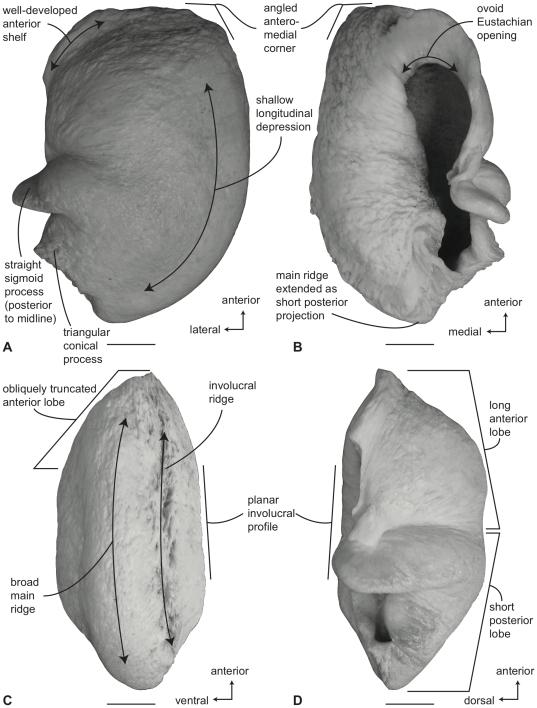
Right tympanic bulla of *Balaenoptera omurai* (NSMT 32505). A. ventral, B. dorsal, C. medial and D. lateral views. Scale bar  = 2 cm.

In comparison to other balaenopterid species, the tympanic bulla of *B. omurai* is intermediate in size (Table S3). In ventral view (Figure 22A) the medial half of the ventral side is slightly depressed posteriorly, but not as deeply as in balaenids. The anterolateral margin of the ventral surface is developed as an obliquely oriented shelf. A rather broad lateral furrow is transversely directed. The surface of the anterior lobe is marked by a well-developed anterolateral shelf, which is strongest anteriorly. The sigmoid process has a nearly straight posterior margin with a thickened tympanic lip. The conical process is relatively short in height (Table S5) and distinctly triangular in profile, closely resembling the conical process of *Megaptera novaeangliae*. The anterior and medial bullar margins meet at a relatively low angle, thus imparting a more rhomboid shape to the bulla than seen in other species of *Balaenoptera*.

In dorsal view (Figure 22B) the involucral ridge is distinct and retracted laterally from the medial margin of the bulla, which is formed by the main ridge. The main ridge forms both the anterior and posterior ends of the bulla, and is extended as a short posterior projection as in *B. borealis*. Posteriorly, the involucral ridge is not retracted from the posterior end. The surface of the involucrum adjacent to the Eustachian opening is slightly convex, and posteriorly there is no dorsal posterior prominence. Transverse creases on this surface are well developed. The Eustachian opening is ovoid. The conical process is thicker posteriorly than anteriorly (Table S3), with a flattened (not rounded) dorsal surface. The sigmoid process is positioned posterior to the midline.

In medial view (Figure 22C), the main ridge is dorsoventrally broad and parallels the involucral ridge for its entire length. The main ridge is relatively uniform in thickness, broadly rounded transversely, and set off from the ventral surface by a slightly depressed longitudinal flexion. The involucral surface is planar to slightly convex and lacks a dorsal posterior prominence.

In lateral view (Figure 22D), the anterior lobe is slightly shorter than the posterior lobe (Table S4). In addition, the anterior lobe has an obliquely truncated profile accentuated by the well-developed anterolateral shelf. The posterior lobe of the bulla is relatively smooth and not rugose. At the posterior end of the bulla, the main ridge extends as a short projection similar to the condition in *B. borealis*.

#### Balaenopteridae, *Balaenoptera omurai*: Petrosal

**Figure 23:**
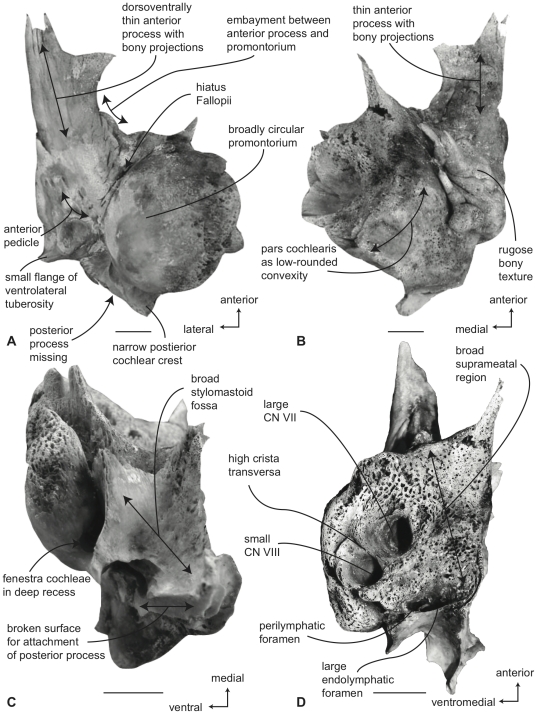
Right petrosal of *Balaenoptera omurai* (NSMT 32992). Posterior process missing. A. ventral, B. dorsal, C. posterior and D. dorsomedial view. Scale bar  = 2 cm.

In ventral view (Figure 23A) the flange of the ventrolateral tuberosity of the anterior process of the petrosal is small and globular. The body of the anterior process is unique among balaenopterids in its flattened, elongated trapezoidal shape with terminal bony projections. Longitudinal creases occur along the apex on the anterior process, which itself is dorsoventrally compressed and blade-like in medial aspect. The promontorium is narrowly attached to the anterior process, similar to the condition *B. musculus* and *B. physalus*. The area of contact is variably filled with irregular bony extensions from the medial edge of the anterior process. The anteroposterior length of the anterior process is only slightly greater than that of the pars cochlearis (Table S6). The promontorium is intermediate in degree of inflation between the globular promontorium of *B. musculus* and the more flattened promontorium of *B. acutorostrata*. The promontorium is more circular in outline than other species of *Balaenoptera* and consequently, the transverse and anteroposterior diameters are subequal. The hiatus Fallopii opens through the ventral surface of the promontorium medial to the juncture between the promontorium and the anterior process. The groove for the tensor tympani muscle is recessed along this same juncture. The epitympanic recess is narrow and smooth and lacks a clearly defined malleolar fossa. The posterior cochlear crest of the petrosal is a posteriorly oriented short process that does not contact the tympanic portion of the posterior process. There is no posterior extension of the posterior cochlear crest and the posterior edge of the promontorium is not extended as a flange posterior to the fenestra cochleae. The dorsolateral surface of the posterior cochlear crest is short, thin, and concave and only forms the anterior portion of the floor of the relatively large and hemispherical stapedial fossa. The stapedial fossa is separated from the facial nerve sulcus by a low longitudinal ridge. The composite posterior process is very long, especially in adults. The body of the posterior process is compressed anteroposteriorly and expanded dorsoventrally. A relatively deep and narrow sulcus for CN VII extends laterally on the ventral side of the posterior process.

In dorsal view (Figure 23B) the pars cochlearis is not extended dorsomedially as a pyramidal process, but instead is extended dorsally as a low, rounded convexity. This convexity results in a change in elevation between the surface of the tegmen tympani and the dorsolateral rim of the suprameatal region of the pars cochlearis. The suprameatal region itself is relatively broad and its surface slopes towards the dorsal elevation of the tegmen tympani. There is no suprameatal fossa. The dorsal face of the anterior process has the typical rugose surface texture seen in other balaenopterids. However, there a distinct dense bony projections extending medially from the posteromedial corner of the tegmen tympani. In NSMT M-32992 a bony spine composed of spongy bone extends anterodorsally from the anteromedial corner of the pars cochlearis.

In posterior view (Figure 23C) the fenestra cochleae is tear-drop shaped and lies in a deep fissure recessed into the posterior face of the promontorium, which is extended posteriorly as a swollen region below the fenestra cochleae. A closed seam marked by a distinct suture extends the length of the deep fissure from the dorsal rim of the fenestra cochleae to the posterior rim of the perilymphatic foramen. The fenestra cochleae and fenestra vestibuli are subequal in size. The facial nerve sulcus continues as a deep groove onto the posterior side of the posterior process. The stylomastoid fossa is very broad and deep and demarcated by prominent dorsal and posterior bony margins. The fossa extends only short distance onto the base of the posterior process of the petrosal.

In dorsomedial view (Figures 23D) the internal auditory meatus is large elliptical opening containing similarly sized deep circular openings for CN VII and VIII. The crista transversa is broad and tall and extends to the rim of the internal auditory meatus. Immediately posterior to the internal auditory meatus are the elliptically shaped perilymphatic and endolymphatic foramina. The endolymphatic foramen is a relatively large and narrowly elliptical opening separated from the smaller, more circular perilymphatic foramen by a relatively broad, anteroposteriorly oriented bony septum.

#### Balaenopteridae, *Balaenoptera physalus*: Tympanic bulla

**Figure 24:**
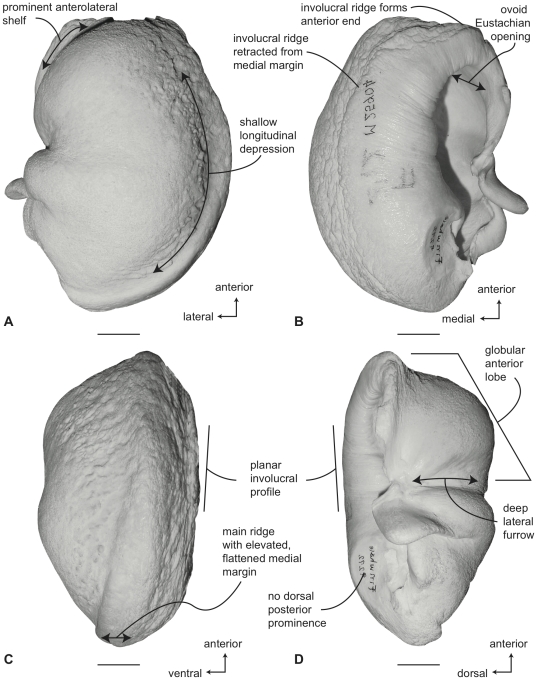
Left (reversed) tympanic bulla of *Balaenoptera physalus* (ZMUC 27A). A. ventral, B. dorsal, C. medial and D. lateral views. Scale bar  = 2 cm.

Relative to other species of *Balaenoptera*, the tympanic bulla of *B. physalus* is large with an inflated anterior lobe (Table S3). In ventral view (Figure 24A) the medial half of the bulla is flattened and depressed immediately adjacent to the broad main ridge. The obliquely oriented ventral shelf is strongly developed anterolaterally. The anterior lobe is slightly shorter than the posterior lobe (Table S4) and the lateral furrow is deeply incised and transversely oriented. The sigmoid process has a sinuous posterior margin with a thickened tympanic lip. The conical process is tall in height and broadly arched (Table S5).

In dorsal view (Figure 24B) the involucral ridge is distinct and retracted laterally from the medial margin of the bulla, which is formed by the main ridge. The main ridge forms the posterior end of the bulla, while the involucral ridge forms the anterior end. Posteriorly, the involucral ridge is only slightly retracted from the posterior end. The surface of the involucrum adjacent to the Eustachian opening is slightly convex. Transverse creases on this surface are variably developed. The Eustachian opening is ovoid and similar in size to that in *B. borealis.* The conical process is posteriorly thickened, (Table S5) with a posterior portion that is broad and shelf-like. The sigmoid process is positioned posterior to the transverse midline.

In medial view (Figure 24C), the main ridge is dorsoventrally broad and parallels the involucral ridge for its entire length. Posteriorly, the main ridge is variably developed as an elevated and flattened plateau similar to the condition in some specimens of *B. musculus*. The involucral surface is relatively planar to slightly concave and lacks a distinct dorsal posterior prominence.

In lateral view (Figure 24D) the strongly developed anterolateral shelf is clearly visible on the ventral surface and is accentuated by the distinctly globular form of the anterior lobe. The profile of the anterior lobe is thus more sinuous than the obliquely truncated profile in *B. acutorostrata*. The posterior lobe of the bulla is relatively smooth and not rugose.

#### Balaenopteridae, *Balaenoptera physalus*: Petrosal

**Figure 25:**
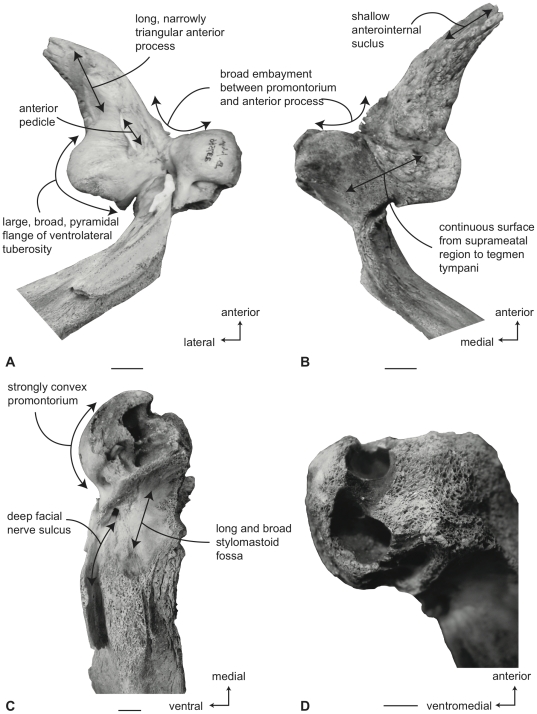
Left (reversed) petrosal of *Balaenoptera physalus* (USNM 237566). Distal portion of posterior process not shown. A. ventral, B. dorsal, C. posterior and D. dorsomedial views. Scale bar  = 2 cm.

In ventral view (Figure 25A) the flange of the ventrolateral tuberosity of the anterior process of the petrosal is relatively large, dorsoventrally thickened, and broadly pyramidal, although more globular than in *B. musculus*. The anterior process is relatively large and extremely elongate with a narrowly triangular apex. The anterior process and the promontorium are separated by a distinct anteromedial embayment. This condition is also seen in *B. musculus* among extant balaenopterids. In other balaenopterids (except *B. omurai*) the anterior process is broadly attached to the anterior margin of the promontorium with a convex dorsomedial rim. The medial margin of the anterior process is relatively smooth, but in some specimens possesses short, irregular, medially directed bony extensions at the point of contact between the promontorium and anterior process. This is similar to the condition in some specimens of *B. musculus*, although in the latter taxon the bony extensions generally are more elaborate. The anteroposterior length of the anterior process is greater than that of the pars cochlearis (Table S6). The promontorium is globular as in *B. musculus* and unlike the more flattened promontoria of other species of *Balaenoptera*. The transverse diameter of the promontorium is only slightly greater than the anteroposterior diameter, although with *in situ* petrosals with preserved dorsomedial extensions the difference is much greater. The hiatus Fallopii opens through the ventral surface of the promontorium medial to the juncture between the promontorium and the anterior process. The groove for the tensor tympani muscle is indistinct. The epitympanic recess is broad and smooth and lacks a clearly defined malleolar fossa. The posterior cochlear crest of the petrosal is a posteriorly oriented elongate process and does not contact the tympanic portion of the posterior process. The posterior extension of the posterior cochlear crest is continuous medially with the posterior edge of the promontorium, which is extended as a narrow (not broad) flange posterior to the fenestra cochleae. The dorsolateral surface of the posterior cochlear crest is thin and concave and forms the floor of the relatively large stapedial fossa. Unlike the condition in *B. musculus* the stapedial fossa is not excavated into the posterior cochlear crest and is separated from the secondary facial sulcus. The composite posterior process is very long, especially in adults. The body of the posterior process is compressed anteroposteriorly and expanded dorsoventrally. A relatively deep sulcus for CN VII extends laterally onto the ventral side of the posterior process and in some specimens is nearly completely enclosed ventrally by a bony floor.

In dorsal view (Figure 25B) the pars cochlearis is not extended dorsomedially as a pyramidal process, but instead forms a continuous, flattened surface with the tegmen tympani. Consequently, there is no suprameatal fossa and the suprameatal region itself is merely the medial portion of the flattened dorsal surface. The dorsal face of the anterior process has the typical rugose surface texture seen in other balaenopterids. In some specimens, the marginal anterointernal sulcus is weakly developed on the anteromedial edge of the elongated anterior process.

In posterior view (Figure 25C) the fenestra cochleae is a relatively small, teardrop shaped opening, only slightly recessed into the posterior face of the promontorium. Dorsally, the fenestra cochleae is well separated from the perilymphatic foramen. In adult specimens a distinct, but fused suture extends from the dorsal rim of the fenestra cochleae to the posterior rim of the promontorium [20]. The fenestra cochleae and fenestra vestibuli are nearly equal in size. The facial nerve sulcus continues as a deep groove onto the posterior side of the posterior process. The stylomastoid fossa is broad and demarcated by prominent dorsal and ventral posterior bony margins. The fossa is long and extends onto the base of the posterior process of the petrosal.

In dorsomedial view (Figure 25D) the internal auditory meatus varies from a single, large, elliptical opening containing deeply recessed circular foramina for CN VII and CN VIII to a divided pair of circular openings for CNVII and CN VIII. The crista transversa in adult specimens is thin and low and does not extend beyond the rim of the internal auditory meatus. However, in younger individuals the crista transversa is quite broad and extends to the suprameatal surface. Immediately posterior to the internal auditory meatus are the elliptically shaped perilymphatic and endolymphatic foramina. The foramina are generally of equally large size and closely overlap one another in an *en echelon* arrangement separated by a thin, mediolaterally oriented bony septum. In this view the sharply truncated dorsomedial rim of the promontorium is clearly evident with a rim of cancellous bone marking the area where the dorsomedial bony extension of the pars cochlearis has been broken away.

#### Balaenopteridae, *Megaptera novaeangliae*: Tympanic bulla

**Figure 26:**
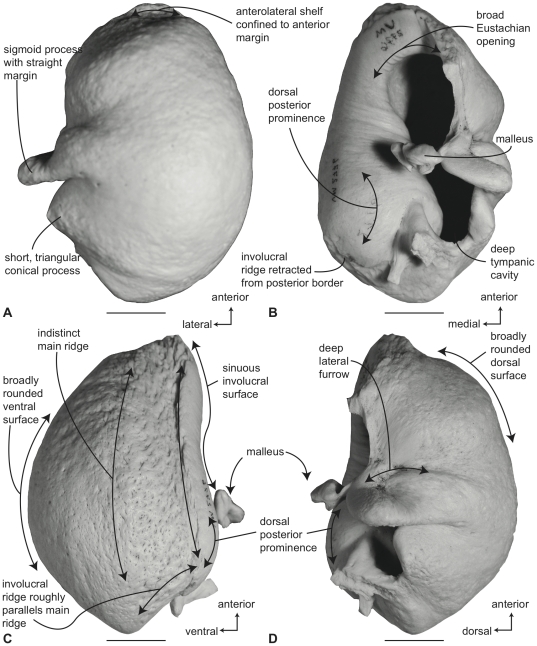
Left (reversed) tympanic bulla of *Megaptera novaeangliae* (VM 2776). A. ventral, B. dorsal, C. medial and D. lateral views. Scale bar  = 2 cm.

Relative to other balaenopteroid species the tympanic bulla of *M. novaeangliae* is intermediate in size and distinctly inflated as reflected in both the broadly convex ventral surface and the enlarged tympanic cavity (Table S3). In ventral view (Figure 26A) the medial half of the bulla is broadly convex and lacks the longitudinal depression immediately adjacent to the main ridge. The anterolateral margin of the ventral surface has a weakly developed anterolateral ventral shelf similar to the condition in *B. musculus*. The anterior lobe is slightly shorter than the posterior lobe (Table S4) and the lateral furrow is distinct and transversely oriented. The sigmoid process has a nearly straight posterior margin with a thickened tympanic lip. The conical process is relatively short in height and resembles that in *B. omurai* in being triangular in external profile (Table S5).

In dorsal view (Figure 26B) the involucral ridge is distinct and retracted laterally from the medial margin of the bulla, which is formed by the main ridge. The main ridge forms the posterior end of the bulla, while the involucral ridge forms the anterior end. Posteriorly, the involucral ridge is well retracted from the posterior end. The surface of the involucrum adjacent to the Eustachian opening is narrow and slightly convex. Posteriorly, the involucral surface is developed as a rounded dorsal posterior prominence. Transverse creases on this surface are poorly developed. The Eustachian opening is relatively large transversely and ovoid. The conical process is thicker posteriorly than anteriorly (Table S5), with a distinctly flattened (not rounded) dorsal surface. The sigmoid process is positioned posterior to the midline.

In medial view (Figure 26C), the main ridge is indistinct on the very broadly rounded medial margin and roughly parallels the sinuous involucral ridge for its entire length. The involucral surface is slightly concave due in part to the presence of the dorsal posterior prominence. The posterior lobe is distinctly inflated ventrally and thus the greatest dorsoventral bullar thickness occurs posteriorly. This inflation of the posterior lobe is evident in the large volume of the tympanic cavity.

In lateral view (Figure 26D) the anterior lobe lacks the obliquely truncated profile of other balaenopterids, a condition accentuated by the weakly developed anterolateral shelf. The posterior lobe of the bulla is relatively smooth and not rugose.

#### Balaenopteridae, *Megaptera novaeangliae*: Petrosal

**Figure 27:**
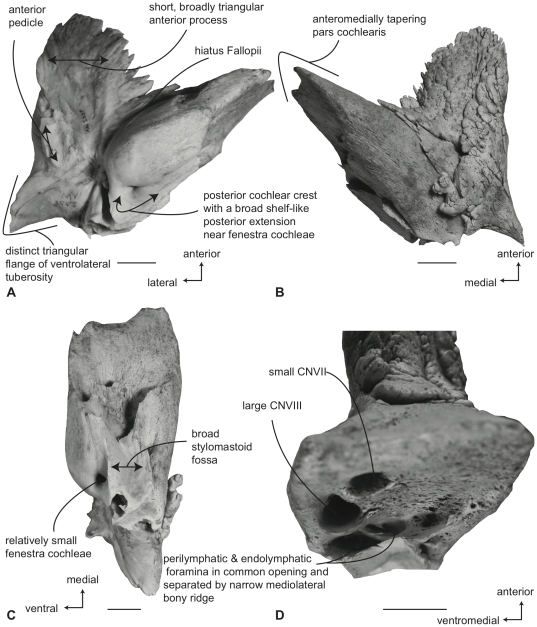
Right petrosal of *Megaptera novaeangliae* (VM 2776). Distal portion of posterior process not shown. A. ventral, B. dorsal, C. posterior and D. dorsomedial. Scale bar  = 2 cm.

In ventral view (Figure 27A) the flange of the ventrolateral tuberosity is relatively small and bluntly triangular in some specimens, while in others (e.g., HSU VM-2776) the flange is sharply and acutely triangular and more elongated. When preserved *in situ*, this projection overlaps the adjacent squamosal rather than simply lying against it. The anterior process is relatively short with a bluntly triangular apex. It is broadly continuous with the promontorium in most specimens (USNM 21492) but less so in others (e.g. USNM 48175, USNM 16252). Irregular bony projections occur on the anteromedial portion of the anterior process in more immature specimens that possess a slight anteromedial embayment between the anterior process and promontorium. A broad, anteroposteriorly oriented sulcus occurs in some specimens on the ventral surface of the anterior process between the groove for the tensor tympanii and the anterior bullar pedicle. The anteroposterior length of the anterior process is greater than that of the pars cochlearis (Table S6). The promontorium is externally convex and intermediate in inflation between the flattened condition in *B. acutorostrata* and the globular condition in *B. physalus*. The transverse diameter of the promontorium is slightly greater than the anteroposterior diameter, although with *in situ* petrosals with preserved dorsomedial extensions, the difference is much greater. In most specimens the pars cochlearis is extended anteromedially as a tapering projection into the cranial hiatus. In more mature specimens this projection seems to be overridden by the smooth surface of the promontorium. The hiatus Fallopii is large in diameter and opens through the ventral surface of the promontorium medial to the juncture between the promontorium and the anterior process. The groove for the tensor tympani muscle is indistinct. The epitympanic recess is broad and smooth and lacks a clearly defined malleolar fossa. The posterior cochlear crest of the petrosal is a posteriorly oriented elongate process that does not contact the tympanic portion of the posterior process. The posterior extension of the posterior cochlear crest is continuous medially with the posterior edge of the promontorium, which is extended as a broad flange posterior to the fenestra cochleae. The dorsolateral surface of the posterior cochlear crest is thin and concave and forms the floor of the relatively large and hemispherical stapedial fossa, which is separated from the adjacent facial sulcus by a low, longitudinal ridge. The composite posterior process is shorter relative to the condition in most balaenopterids. The body of the posterior process is compressed anteroposteriorly and expanded dorsoventrally. A relatively shallow sulcus for CN VII extends laterally onto the ventral side of the posterior process.

In dorsal view (Figure 27B) the pars cochlearis is not extended dorsomedially as a pyramidal process, but instead forms a continuous, flattened surface with the tegmen tympani. Consequently, there is no suprameatal fossa and the suprameatal region itself is merely the medial portion of the flattened dorsal surface. The pars cochlearis, however, possesses an unusual morphology in its anteromedial tapering and elongation. The dorsal face of the anterior process has a very rugose surface texture marked by an irregular series of bony growths. The anterointernal sulcus is absent from the anteromedial edge of the anterior process. However, there is a variably developed anteroexternal sulcus. In some specimens, the anteroexternal sulcus is roofed over to form a bony canal.

In posterior view (Figure 27C) the fenestra cochleae is relatively small, circular, and only slightly recessed into the posterior face of the promontorium. Dorsally, the fenestra cochleae is broadly separated from the perilymphatic foramen, more so than in any other balaenopterid. The fenestra cochleae and fenestra vestibuli are roughly equal in size. The facial nerve sulcus continues as a shallow groove onto the posterior side of the posterior process. The stylomastoid fossa is extremely broad and shallow and demarcated by prominent dorsal and ventral posterior bony margins. The fossa is elongate and it slightly extends onto the base of the posterior process of the petrosal.

In dorsomedial view (Figure 27D) the shape of the internal auditory meatus is influenced by the unusual tapering elongation of the anteromedial corner of the pars cochlearis. As a consequence the internal auditory meatus occupies a sloping surface and depending upon developmental age is either divided by two separate stepped openings, a single elliptical opening, or a single circular opening. In the first two cases the circular openings for CN VII and CN VIII are separated by a broad crista transversa, which reaches or nearly reaches the external surface of the pars cochlearis. In the third case the crista transversa is deeply recessed within the internal auditory meatus. Immediately posterior to the internal auditory meatus is a single slit-like elongation containing the deeply recessed and also elliptically shaped perilymphatic and endolymphatic foramina. The foramina are generally of equal size and closely overlap one another in an *en echelon* arrangement separated by a thin, mediolaterally oriented bony septum. The septum separating the internal auditory meatus from the common, slit-like recess for the perilymphatic and endolymphatic foramina is extremely thin, relative to the condition in other balaenopterids.

#### Eschrichtiidae, *Eschrichtius robustus*: Tympanic bulla

**Figure 28:**
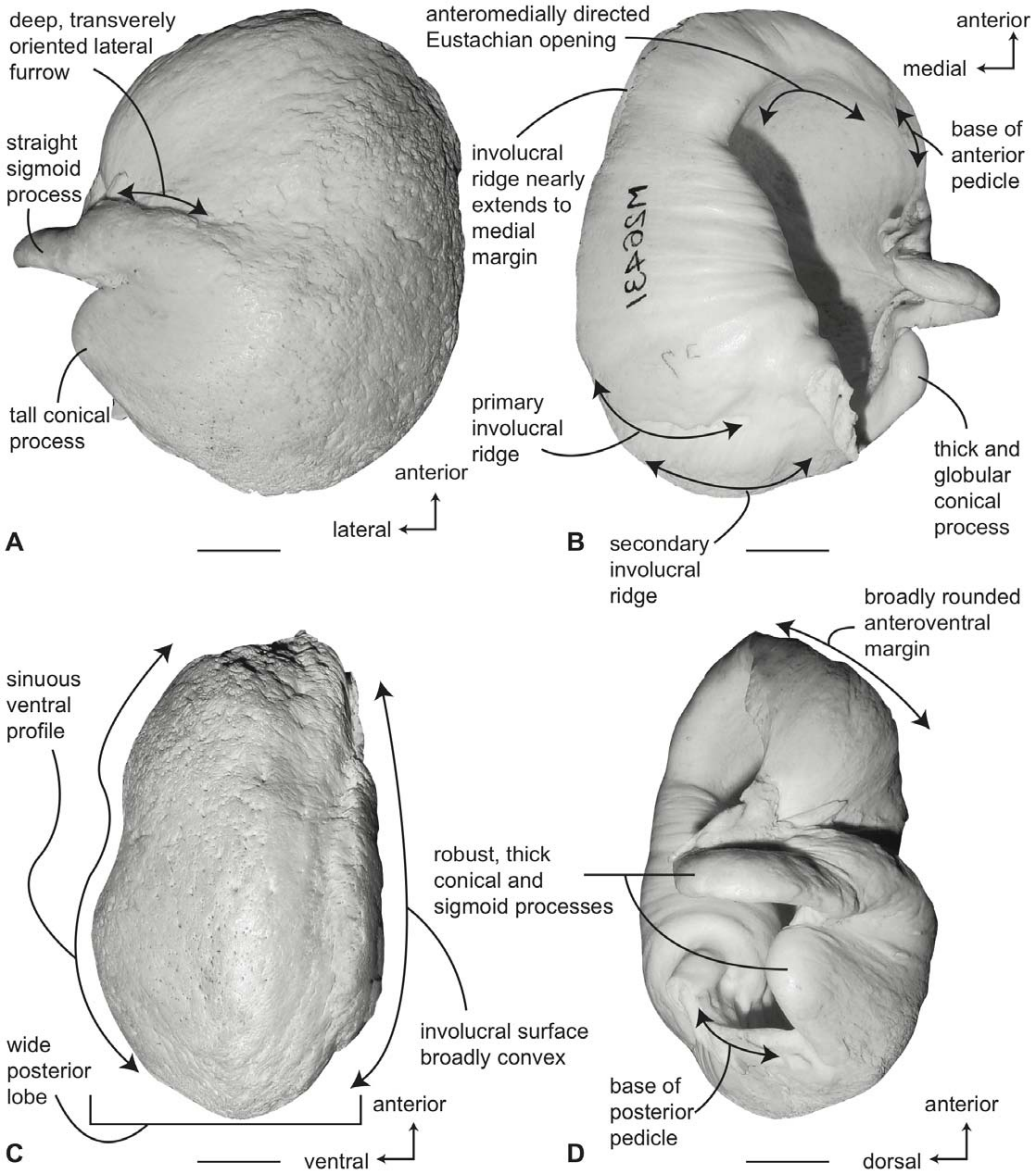
Left (reversed) tympanic bulla of *Eschrichtius robustus* (NSMT 26431). A. ventral, B. dorsal, C. medial and D. lateral views. Scale bar  = 2 cm.

Relative to other balaenopteroids the tympanic bulla of *E. robustus* is more equidimensional in anteroposterior and transverse width (Table S3). In addition, the bulla is more dorsoventrally compressed and has a relatively shallow tympanic cavity with a distinctly reduced volume. In ventral view (Figure 28A) the medial half of the ventral surface is convex. The anterior lobe is only slightly shorter than the posterior lobe (Table S4). A deep lateral furrow is present and transversely directed. The surface of the anterior lobe is rounded and convex and lacks any trace of an anterolateral shelf. The sigmoid process has a nearly straight posterior margin with a thickened tympanic lip. The conical process is relatively tall in height (Table S5) and broadly arched. The sigmoid fissure is especially well developed.

In dorsal view the involucral ridge is distinct and only slightly retracted laterally from the medial margin of the bulla, which is formed by the main ridge. Both the main and involucral ridges are involved in formation of the anterior and posterior ends of the bulla. Posteriorly, the involucral ridge is relatively complex and developed as overlapping primary and secondary ridges (Figure 28B). In some specimens (HSU VM-6443) a similar condition occurs at the anterior end where a secondary involucral ridge is rather well developed and extended as an anteromedially directed keel. The surface of the involucrum adjacent to the Eustachian opening is slightly convex. This is also the case posteriorly. There is no dorsal posterior prominence. Transverse creases on the involucral surface are well developed. The Eustachian opening is lobate and oriented anteromedially rather than directly anteriorly. The conical process is uniformly thick and globular (Table S5). The sigmoid process is positioned posterior to the transverse midline.

In medial view (Figure 28C), the main ridge is narrow and parallels the involucral ridge for its entire length. The involucral surface is broadly convex and dorsoventrally swollen. The ventral surface is distinctly sinuous with a dorsoventrally broader posterior lobe behind the relatively elongated lateral furrow.

In lateral view (Figure 28D) the anteroventral portion of the anterior lobe is broadly rounded and lacks the obliquely truncated profile of most species of *Balaenoptera*. The posterior lobe of the bulla is relatively smooth and not rugose.

#### Eschrichtiidae, *Eschrichtius robustus*: Petrosal

**Figure 29:**
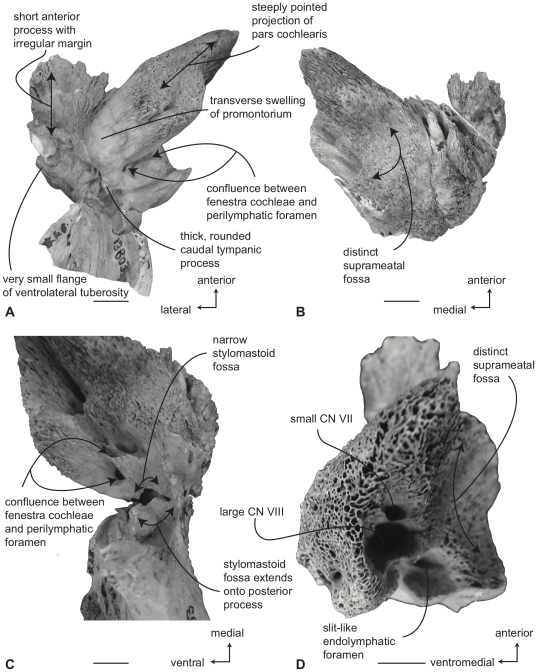
Right petrosal of *Eschrichtius robustus* (USNM 13803). Distal portion of posterior process not shown. A. ventral, B. dorsal, C. posterior and D. dorsomedial views. Scale bar  = 2 cm.

In ventral view (Figure 29A) the flange of the ventrolateral tuberosity of the anterior process of the petrosal is extremely reduced and the region of the tegmen tympani lateral to the anterior pedicle is a narrow rounded edge thicker dorsoventrally and transversely. The anterior process is very short with a bluntly triangular apex reminiscent of the condition in *M. novaeangliae*. There is a distinct anteromedial embayment between the medial edge of the anterior process and the promontorium, most noticeable in specimens from more mature individuals. The anteroposterior length of the anterior process is less than that of the pars cochlearis (Table S6) in contrast to the opposite condition seen in all other balaenopteroids. The surface of the promontorium is neither globular nor flattened but instead consists of roughly planar anterior and posterior surfaces that meet in a transversely oriented flexure or ridge positioned midway between the anterior and posterior margins of the promontorium. The development of this ridge varies from a low longitudinal swelling to a more prominent divide most strongly developed medially. The transverse diameter of the promontorium is distinctly greater than the anteroposterior diameter, especially in specimens from mature individuals. The hiatus Fallopii opens through the ventral surface of the promontorium medial to the juncture between the promontorium and the anterior process. The groove for the tensor tympani muscle is distinct and paralleled by a crease along the anterolateral margin of the promontorium. The epitympanic recess is relatively broad with an indistinct smooth malleolar region and a narrow and pitted incudal fossa. The posterior cochlear crest is a posteriorly oriented and relatively short and blunt process that does not contact the tympanic portion of the posterior process. There is no flange-like posterior extension of the posterior cochlear crest and consequently the posterior edge of the promontorium adjacent to the fenestra cochleae is low and nearly at the same level as the narrow stylomastoid fossa. The dorsolateral portion of the posterior cochlear crest is thick and concave and forms the floor of the very large and hemispherical stapedial fossa, which is well separated from the adjacent facial sulcus. The composite posterior process is shorter relative to the condition in most balaenopteroids and becomes very broad distally. The body of the posterior process is compressed anteroposteriorly and expanded dorsoventrally. A relatively deep sulcus for CN VII extends laterally onto the ventral side of the posterior process.

In dorsal view (Figure 29B) the pars cochlearis is not extended dorsomedially as a pyramidal process, but instead is flattened to form a suprameatal fossa. This fossa is separated from the tegmen tympani along an anteroposteriorly directed flexure such that the dorsal surface of the tegmen tympani is actually facing dorsolaterally. This difference in orientation between the pars cochlearis and tegmen tympani is also obvious on the ventral side of the petrosal where the ventral surfaces of the promontorium and anterior process are not subparallel but rather meet at distinct angle. The dorsal face of the anterior process has a relatively smooth texture and in some specimens is crossed anteroposteriorly by the sinuous anteroexternal sulcus.

In posterior view (Figure 29C) the fenestra cochleae is teardrop shaped and broadly confluent with the perilymphatic foramen. The deeply recessed channel connecting the two openings is broadly open and no specimens of *E. robustus* have been observed in which the channel is ossified. The fenestra cochlea and fenestra vestibuli are roughly equal in size. The fenestra vestibuli is separated by a bony strut from the short, deep facial sulcus, which is discontinuous from the fenestra vestibuli across the stapedial fossa and onto the posterior side of the posterior process. The stylomastoid fossa is narrow and irregularly shaped with an indistinct ventral margin and a more prominent dorsal margin. The fossa is relatively short, but does extend onto the base of the posterior process of the petrosal.

In dorsomedial view (Figure 29D) the internal auditory meatus is variably developed probably based on developmental age. Younger individuals typically have separate openings for CN VII and CN VIII and a broad crista transversa that reaches the endocranial surface, while older individuals tend to have a single oblong opening with each opening deeply recessed and separated by a retracted crista transversa. In all specimens examined the opening for CN VIII is circular and much larger than the smaller and more oval opening for CN VII. The latter also tends to preserve an anterior fissure reminiscent of structures described previously in certain fossil mysticetes [11]. Posterior to the internal auditory meatus but separated from it by a relatively thick bony septum is the small, elliptical perilymphatic foramen with the open channel connecting it with the fenestra cochlea. The slit-like endolymphatic foramen is placed dorsal to the perilymphatic foramen with the two opening separated by a thin, mediolaterally oriented bony septum. These openings overlap one another in an *en echelon* arrangement.

### Phylogenetic Analysis and Discussion

Based on our anatomical descriptions and comparisons, it is evident that petrotympanic morphology is diagnostic for individual species of extant mysticetes. Although a few of the species can be distinguished based solely on features of the bulla or petrosal alone, the majority are identifiable by features of the combined petrotympanic complex. As a practical expression of the diagnostic aspect of mysticete petrotympanics, we have provided a dichotomous key for identifying extant mysticete ear regions as Text S1.

**Figure 30:**
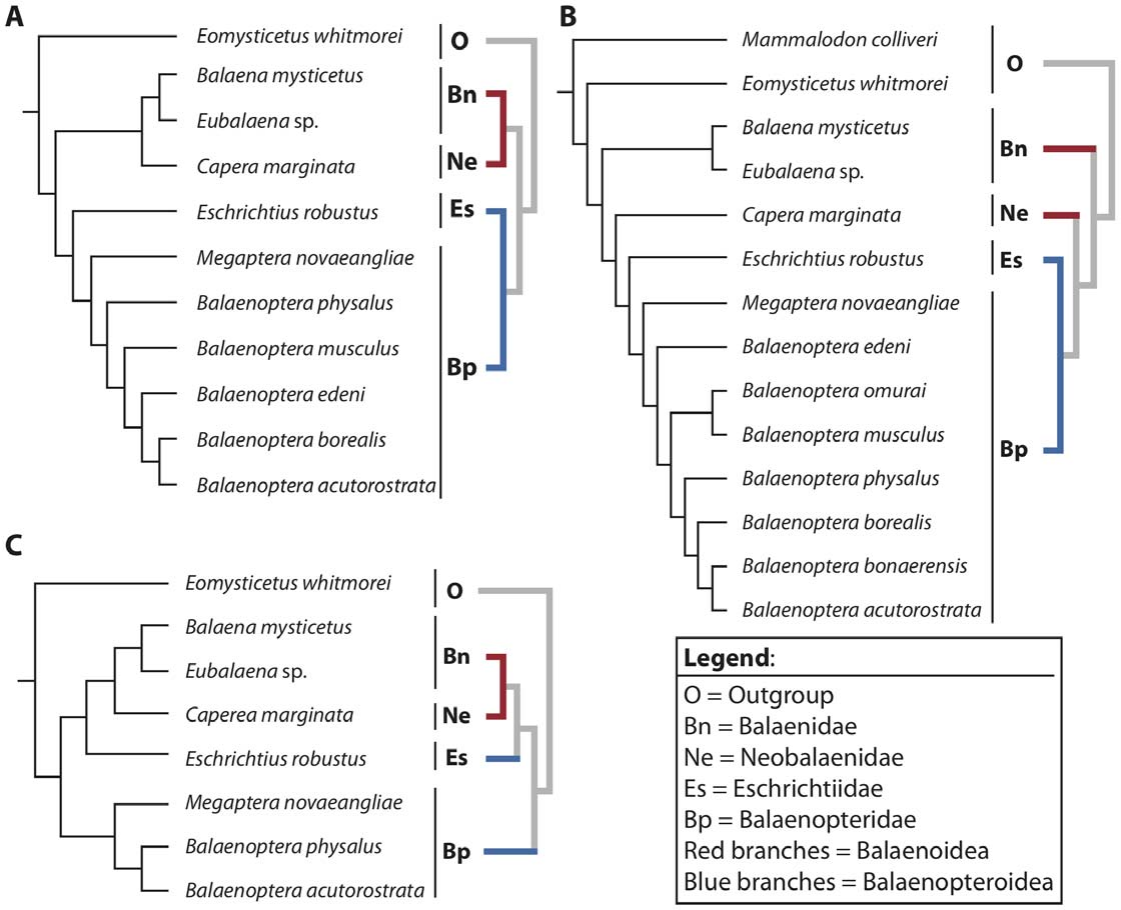
Competing hypotheses of extant mysticete relationships. A. Monophyly of Balaenoidea and Balaenopteroidea (modified from Deméré et al. [69]), B. Monophyly of Balaenopteroidea, but not Balaenoidae (modified from Marx [70]), C. Monophyly of Balaenoidea, but not Balaenopteroidea (modified from Bouetel and Muizon [71]).

Given the diagnostic nature of the mysticete petrotympanic complex, we hypothesize that the ear region of baleen whales is phylogenetically informative, as has been suggested for odontocetes and many terrestrial mammals. In order to demonstrate the phylogenetic significance of mysticete petrotympanic anatomy, we chose to test three competing published hypotheses of mysticete relationships [69]–[71] by using only petrotympanic characters. The three hypotheses differ in the affinities of the four major extant clades, namely Balaenidae, Balaenopteridae, Eschrichtiidae, and Neobalaenidae (Figure 30). In the first hypothesis (Figure 30A), Neobalaenidae is placed as the sister taxon to Balaenidae (the two constituting the clade Balaenoidea), and Eschrichtiidae is placed as the sister taxon to Balaenopteridae (the two constituting the clade Balaenopteroidea). Such a result is consistent with traditional classifications based on morphology [21], [23], [49], [69], [72]–[74]. In a second hypothesis (Figure 30B), Balaenoidea is refuted, and Balaenidae and Neobalaenidae are placed as successive sister taxa to Balaenopteroidea. Under this hypothesis, which is supported by some morphological [70], molecular [75]–[77], and combined data [78], Balaenoidea is paraphyletic. A third alternative hypothesis based on morphology alone (Figure 30C) [71] preserves monophyly of Balaenoidea, but places Balaenopteridae and Eschrichtiidae as successive sister taxa, thereby recovering a paraphyletic Balaenopteroidea.

In our test of the three hypotheses, we identified 48 morphological characters (Text S2) from the petrotympanic complex, which include some that have been used in previous studies and others that are newly described in the current paper, and these characters were scored for all extant mysticete species (Table S7). We used two extinct mysticetes as outgroups, which are the toothed taxon *Mammalodon colliveri* from the Oligocene of southeastern Australia [24] and the edentulous mysticete *Eomysticetus whitmorei* from the Oligocene of southeastern North America [79].

**Figure 31:**
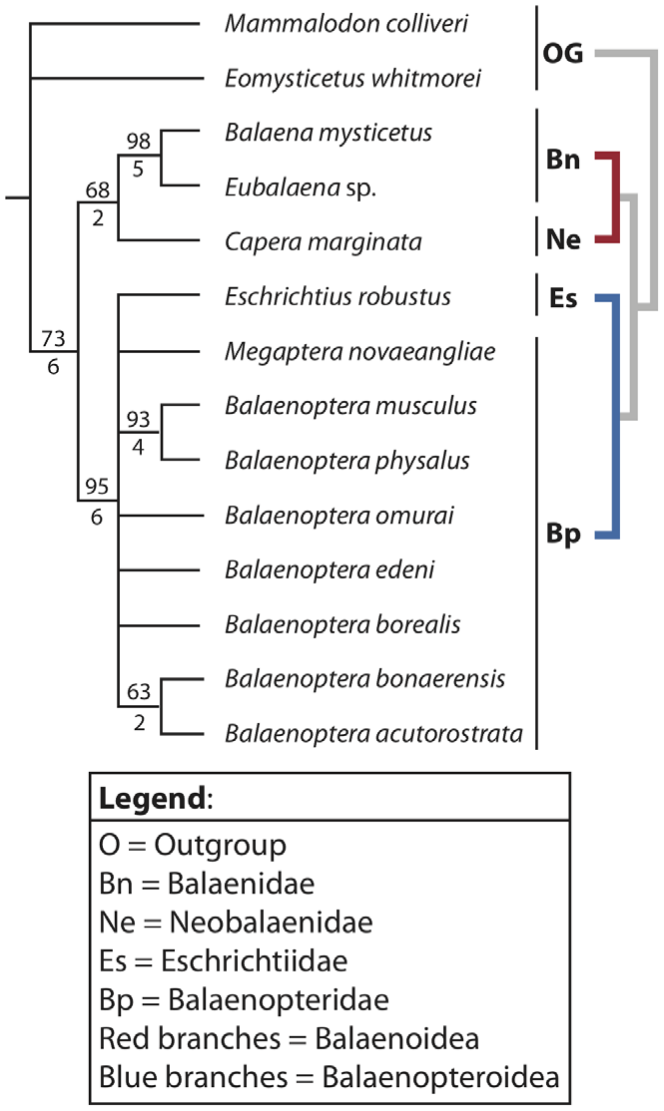
Strict consensus of three most parsimonious trees recovered from analysis of petrotympanic characters. Numbers above and below branches refer to bootstrap values and number of additional steps required to collapse the branch respectively.

The results of our analysis using only characters from the petrotympanic complex yielded three most parsimonious trees that recovered high-level relationships among extant mysticetes. Within our strict consensus tree (Figure 31), monophyly of Balaenidae was recovered with *C. marginata* (Neobalaenidae) as its sister taxon (supporting Balaenoidea monophyly), but balaenopteroid relationships were unresolved. However, a monophyletic Balaenopteroidea was recovered as a polytomy (Figure 31) that included two resolved clades (*B. acutorostrata* plus *B. bonaerensis* and *B. musuculus* plus *B. physalus*). These results support the Balaenoidea plus Balaenopteroidea hypothesis [69] discussed above (Figure 30A), and suggest that features of the petrotympanic complex are useful in addressing phylogenetic questions. Monophyly of Balaenopteroidea was strongly supported in bootstrap analyses (recovered from 95% of 1000 pseudoreplicates), as was Balaenidae monophyly (98%), but there was less support for Balaenoidea (68%). Although only two additional steps are needed to collapse the branch leading to Balaenoidea, as well as to support the *C. marginata* plus Balaenopteroidea hypothesis (Figure 30B) [70], a total of six additional steps are needed to collapse Balaenopteroidea, and an additional two are required (total of 8 steps) to support the *E. robustus* plus Balaenoidea hypothesis (Figure 30C) [70].

Because there was no consensus surrounding balaenopteroid relationships from the analyses that we performed, we have limited our discussion of character support to those major mysticete lineages that were resolved (crown Mysticeti, Balaenoidea, Balaenidae, and Balaenopteroidea). We also provide a brief discussion concerning the lack of resolution within Balaenopteridae.

Crown Mysticeti was supported by bootstrap analyses (73%) and three unambiguous synapomorphies from the petrotympanic complex. Those characters include elongation of the main ridge to the anterior end of the bulla (character 3, state 1; Table S7), presence of the posterior cochlear crest (character 26, state 1), and loss of the fossa for the malleus (character 43, state 2); however, the fossa for the malleus is partially defined in balaenids (state 1). The majority of recently published mysticete phylogenies recover monophyly of crown Mysticeti to the exclusion of *Mammalodon* and *Eomysticetus* (Figure 30) [69]–[71], [78], and such a relationship is not contentious.

Although most morphological studies recover balaenoid monophyly [21], [23], [49], [69], [71]–[74], the majority of molecular studies [75]–[78], plus at least one phylogenetic study based entirely on morphological data [70], support a closer relationship between *C. marginata* and Balaenopteroidea (Figure 30B). Balaenoidea was not strongly supported in bootstrap analyses that we performed (68%), but there are two unambiguous synapomorphies that unite the group. Both Balaenidae and Neobalaenidae (*C. marginatia*) share the rhomboid shape of the tympanic bulla (character 1, state 1) and orientation of the posterior process at a right angle to the long axis of the pars cochlearis (character 25, state 1). Neither of these character states were observed in any other extant mysticete that we examined for the currenty comparative study. The short anterior lobe (character 14, state 1) and the weakly developed conical process of the tympanic bulla (character 8, state 0) that characterize balaenids and neobalaenids likely are other synapomorphies for Balaenoidea, although we were unable to accurately measure these features for the outgroup taxa in order to polarize the two characters.

A monophyletic Balaenoidea is consistent with most previous morphological studies. In the morphological analysis disputing balaenoid monophyly proposed by Marx [70] (Figure 30B), the placement of *C. marginata* with Balaenopteroidea was supported by six unambiguous synapomorphies. Of those six characters, one was taken from the ear region (transverse elongation of the pars cochlearis; character 84 of that study), and we included that character in our analysis (character 27 of our study) as well. However, we differ in our interpretation of pars cochlearis elongation in *C. marginata* (scored as absent here but as present in the previous study). The transverse elongation of the pars cochlearis that characterizes the petrosal of balaenopteroids is associated with a medial flaring of the promontorium (e.g., Figures 13, 27, and 29). In the plesiomorphic condition (absence of elongation), no such flaring is observed. Rather, the promontorium of balaenids and extinct taxa (e.g., *Mammalodon* and *Eomysticetus*) is much more globular, and almost boxy (Figures 7 and 9). In this regard, the promontorium of *C. marginata* (Figure 11) appears much more similar to balaenids than it does to balaenopterids, including those that have ventrally rounded promontoria, such as *B. musculus* (as opposed to the flattened promontorium of *B. bonaerensis*). It is unlikely that rescoring the character for *C. marginata* would overturn the Marx's hypothesis [70]; however, we would be remiss to not comment on our contradictory observation of the mysticete ear region. In any case, the discrepancies among analyses of mysticete relationships highlight the need for more in depth studies of the morphology of both extant and extinct mysticetes.

Monophyly of Balaenidae has been well supported by both molecular and morphological data [69]–[71], [73], [77]–[78], [80]–[85], and the clade was recovered with strong bootstrap support (98%) in our analysis (Figure 31). In addition to the bootstrap analyses, Balaenidae was supported by dorsoventral compression of the tympanic bulla (character 2, state 1), a hypertrophied flange of the ventrolateral tuberosity (character 18, state 3), dorsal extension of the junction between the tegmen tympani and pars cochlearis to form a robust pyramidal structure (character 24, state 1), enlargement if the fenestra cochleae relative to the fenestra vestibule (character 36, state 1), narrow separation between the endolymphatic foramen and fenestra cochleae (character 39, state 1), and development of an acute angle between the posterior process and the ventrolateral tuberosity of the anterior process (character 48, state 2).

Balaenopteroidea (the monophyletic group composed of Eschrichtiidae and Balaenopteridae) is less controversial than Balaenoidea, but the results of at least one morphological study [71] support a closer relationship between Eschrichtiidae and Balaenoidea (Figure 30C). However, the results of our analysis strongly support balaenopteroid monophyly (bootstrap value of 95%). Nine unambiguous synapomorphies unite the group, including an enlargement of the flange of the ventrolateral tuberosity (character 18, state 1), attachment of the anterior process to the promontorium (character 19, state 1), shape of the apex of the anterior process of the petrosal (character 20, state 2), transverse elongation of the pars cochlearis (character 29, state 0), elevation of the suprameatal region of the petrosal to a level dorsal to the anterior process (character 34, state 1), an *en echelon* arrangement of the perilymphatic and endolymphatic openings (character 38, state 1), and an acute angle between the posterior process and the flange of the ventrolateral tuberosity (character 48, state 1). It should be noted that several of these characters, such as shape of the apex of the anterior process and attachment of the process to the promontorium, vary within the genus *Balaenoptera* (Table S7).

Within Balaenopteroidea, the failure to recover a fully resolved balaenopterid clade underscores the limitations of our analysis based solely on the petrotympanic complex. However, a phylogenetic signal is clearly evident in the three most parsimonious ‘ear’ trees (Figure S1). Among these trees, the primary difference is the conflicting placements of *E. robustus* and *M. novaeangliae*. In trees 1 and 2, these taxa form a clade nested among other balaenopteroid species (sister to *B. borealis* in tree 1 and sister to *B. musculus-B. physalus* in tree 2). In contrast, *M. novaeangliae* was placed as the sister taxon to all species of *Balaenoptera* with *E. robustus* excluded from Balaenopteridae all together in tree 3 (Figure S1). The later topology is reminiscent of traditional morphological hypotheses (Figure 30A).

**Figure 32:**
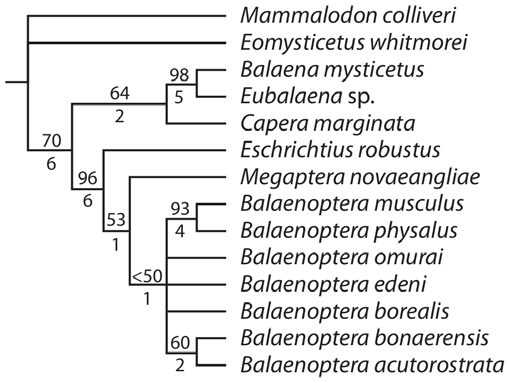
Strict consensus of six most parsimonious trees recovered from analysis of petrotympanic characters when character 45 (position of hiatus Fallopii) is excluded. Numbers above and below branches refer to bootstrap values and number of additional steps required to collapse the branch respectively.

Only a single unambiguous synapomorphy was found to unite *M. novaeangliae* and *E. robustus* (hiatus Fallopii within the juncture between the anterior process and promontorium; character 45, state 1). If character 45 is excluded from the phylogenetic analyses, both *M. novaeangliae* and *E. robustus* fall outside a clade composed of all species of *Balaenoptera* (Figure 32) in all of the six most parsimonious trees that are recovered (Figure S2). We interpret this result as a conflicting phylogenetic signal within the ear region, but the majority of petrotympanic characters support monophyly of both *Balaenoptera* and Balaenopteridae.

It is clear from our analysis that the petrotympanic complex exhibits a phylogenetic signal, even if only at a gross level. The morphology of the auditory region alone can support monophyly of the two traditional lineages of Mysticeti, which are Balaenoidea and Balaenopteroidea, and which is a result that follows traditional interpretations of mysticete relationships while competing with more recent studies that either place neobalaenids (*C. marginata*) with Balaenopteroidea, or place eschrichtiids (*E. robustus*) with Balaenoidea.

The phylogenetic analyses that we performed involved a restricted region of the mysticete basicranium, and the ingroup consisted of extant taxa only. The problems associated with mysticete phylogeny are not easily solved, but the recent interest in baleen whale evolution and discovery of new mysticete fossils have fostered a renewed optimism. Of course, the addition of more characters from across the full anatomy of the animals that are scored for a thorough taxonomic sampling of extinct and extant mysticetes certainly will help to resolve the evolutionary relationships among extant baleen whales. Our comparative descriptions and introduction of new phylogenetically informative characters of the mysticete petrotympanic complex provides a valuable service to both current and future research in cetacean evolution.

## Materials and Methods

We examined the petrosals and tympanic bullae of all known extant mysticetes, including the bowhead, *Balaena mysticetus*; all three species of the right whale, *Eubalaena*; the enigmatic pygmy right whale, *Caperea marginata*; all nominal species of *Balaenoptera* including the recently described *B. omurai*; the humpback whale, *Megaptera novaeangliae*; and the monotypic gray whale, *Eschrichtius robustus* (Table S1). In the end we chose to combine the anatomical descriptions of species of *Eubalaena* because of the small sample size of available ear bones, a maddeningly high level of overall similarity, and the compounding problems associated with comparing a juvenile ear bone from one species with an adult ear bone from another species.

Within Balaenopteridae, the taxonomic validity of *Balaenoptera brydei* is questionable, and the name may be a junior synonym of *B. edeni* [86]. Although there is some molecular support for division of the two species [80], [87], the statistical power for the separation is weak, prompting the IUCN to tentatively consider them the same species, using the name *B. edeni* following ICZN guidelines of priority [88], [89]. Consequently, we treat *B. brydei* and *B. edeni* as conspecifics in our descriptions.

We identified 48 morphological characters within the petrotympanic complex (Text S2) and coded the characters for all extant mysticete species (Table S7). Twenty-one out of the 48 characters are unique to this study. The data matrix (with expanded illustrated character descriptions) has been submitted to the online MorphoBank data depository (www.morphobank.org) [90]. Phylogenetic analyses were preformed in Paup [91] using the maximum parsimony criterion, and the extinct taxa *Mammalodon colliveri* (toothed mysticetes) and *Eomysticetus whitmorei* (edentulous mysticete) were chosen as outgroup taxa. Bootstrap analyses included 1000 pseudoreplicates.

## Supporting Information

Figure S1 **Three most parsimonious trees recovered from phylogenetic analysis of 48 petrotympanic characters.** Major difference between topologies lies with *Eschrichtius robusts* and *Megaptera novaeangliae* (in bold).
(TIFF)

Figure S2 Six most parsimonious trees recovered from phylogenetic analysis of petrotympanic characters excluding #45 (location of hiatus Fallopii).
(TIFF)

Table S1 List of extant mysticetes studied (institutional abbreviations in text).
(PDF)

Table S2 Measurements (mm) of tympani bulla of balaenid and neobalaenid species.
(PDF)

Table S3 Measurements (mm) of tympanic bulla of balaenopterid and eschrichtiid species.
(PDF)

Table S4 Tympanic bulla anterior lobe measurements (mm) among mysticetes.
(PDF)

Table S5 Tympanic bulla conical process measurements (mm) among mysticetes.
(PDF)

Table S6 Petrosal measurements (mm) among mysticetes and reated taxa.
(PDF)

Table S7 **Data matrix of petrotrympanic characters scored for extant mysticetes used in phylogeneti analyses.** Matrix can be downloaded from project page associated with this manuscript at MorphoBank (www.morphobank.org).
(PDF)

Text S1 Dichotomous key for identifying extant species of mysticetes using the petrotympanic complex.
(PDF)

Text S2 **Phylogenetic characters derived from the petrotympanic complex of extant mysticetes.** Further information can be found on the project page associated with this study at MorphoBank (www.morphobank.org).
(PDF)
