# Supplementary figures and images for: The Comparative Osteology of the Petrotympanic Complex (Ear Region) of Extant Baleen Whales (Cetacea: Mysticeti)
Source: PLoS One. 2011 Jun 22;6(6):e21311. doi: 10.1371/journal.pone.0021311 (PMC3120854; doi:10.1371/journal.pone.0021311)

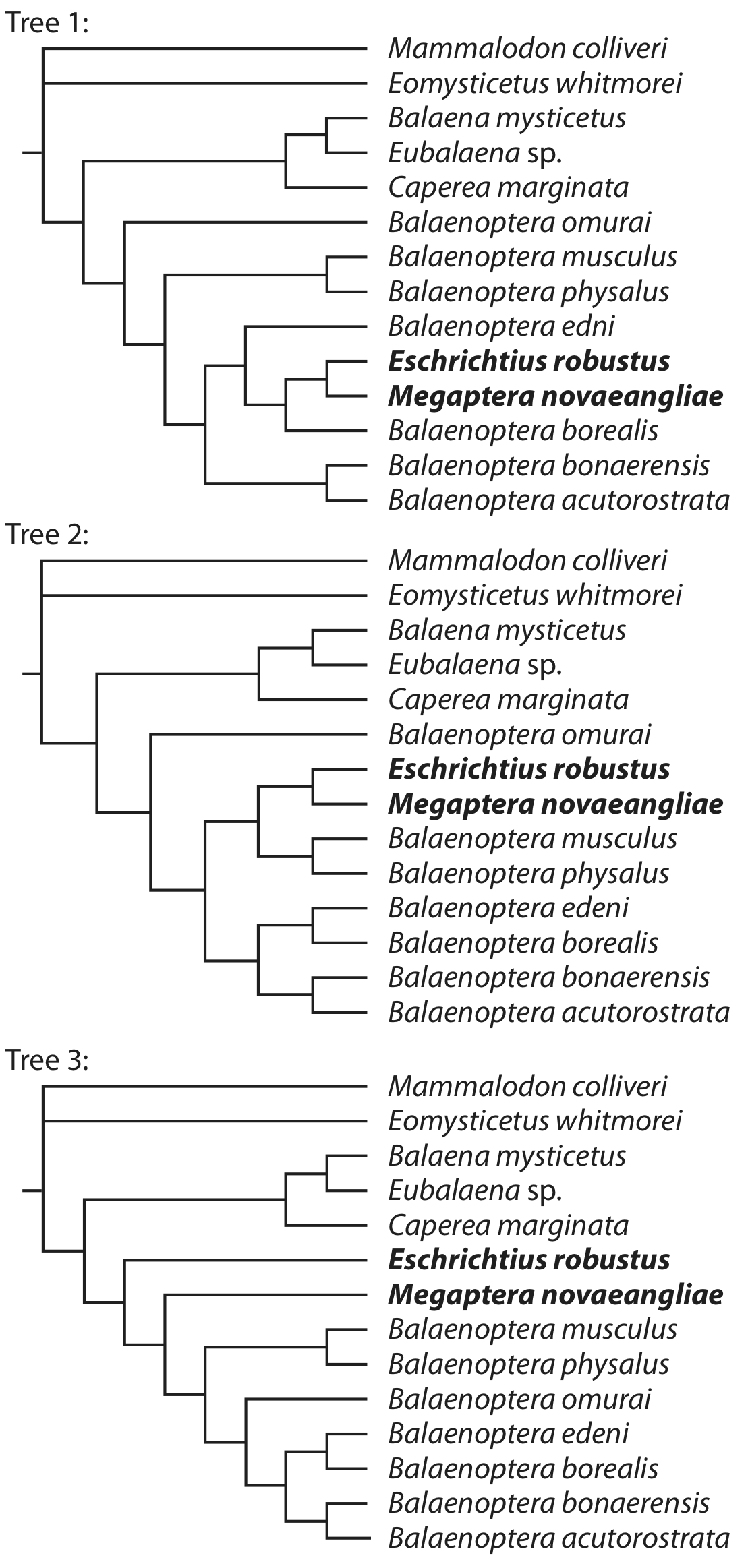

Supplement: Figure S1 — Three most parsimonious trees recovered from phylogenetic analysis of 48 petrotympanic characters. Major difference between topologies lies with Eschrichtius robusts and Megaptera novaeangliae (in bold). (TIFF) [file pone.0021311.s001.tiff]

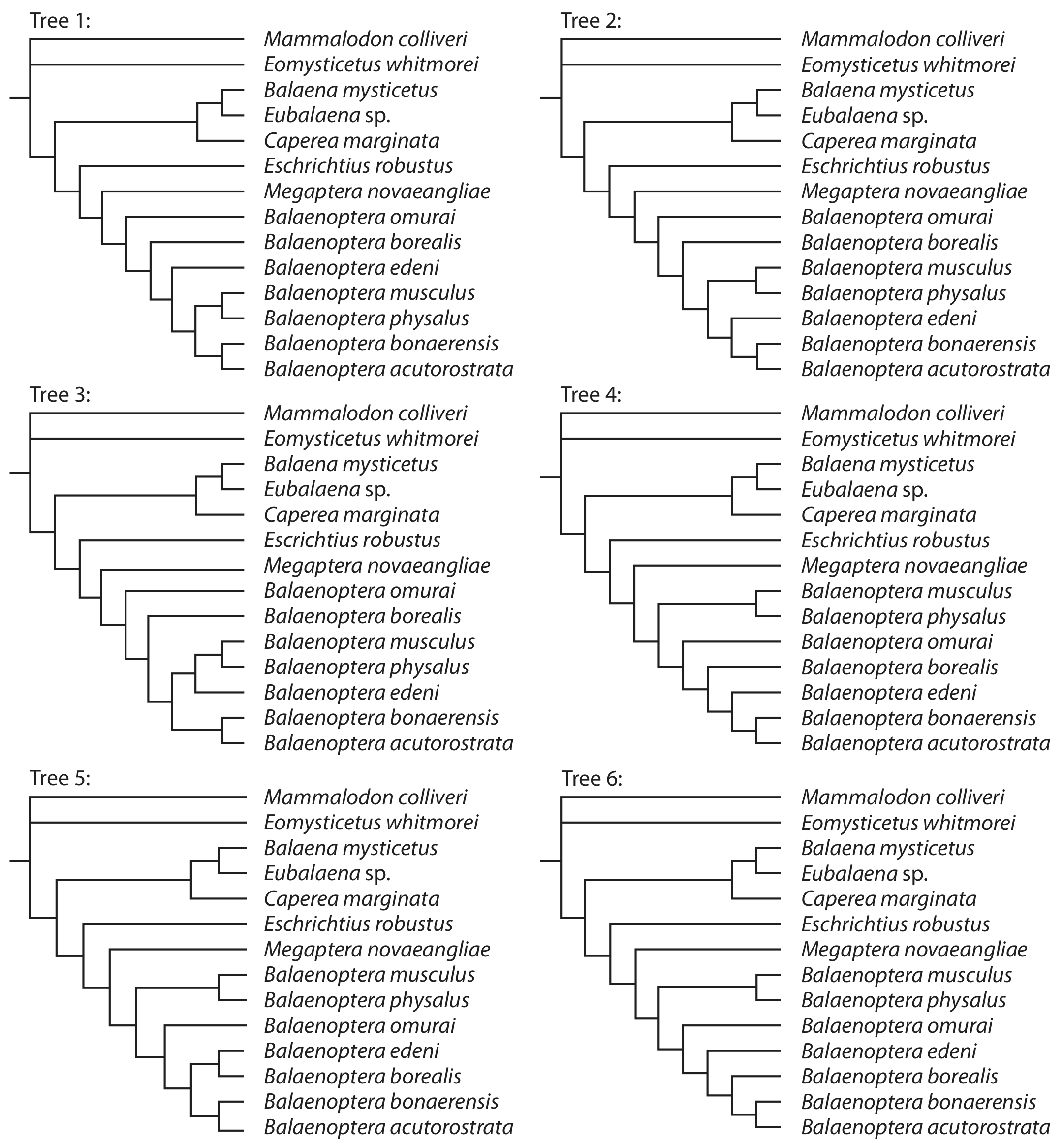

Supplement: Figure S2 — Six most parsimonious trees recovered from phylogenetic analysis of petrotympanic characters excluding #45 (location of hiatus Fallopii). (TIFF) [file pone.0021311.s002.tiff]
