# Supplementary material for: The Comparative Osteology of the Petrotympanic Complex (Ear Region) of Extant Baleen Whales (Cetacea: Mysticeti)
Source: PLoS One. 2011 Jun 22;6(6):e21311. doi: 10.1371/journal.pone.0021311 (PMC3120854; doi:10.1371/journal.pone.0021311)
Supplement: Table S1 — List of extant mysticetes studied (institutional abbreviations in text). (PDF) [file pone.0021311.s003.pdf]

Table S1. List of extant mysticetes studied (institutional abbreviations in text).

Balaenidae

Balaena mysticetus – LACM 84150, 84148 tympanic bulla, LACM 97312 R petrosal (juvenile?); NSMT 25893 R tympanic bulla; SDSNH 23715 L tympanic bulla; USNM 12182 L tympanic bulla; USNM 49407 L tympanic bulla; USNM 63300 L bulla and petrosal, USNM 63301 R petrosal, USNM 15695 L tympanic bulla, USNM 258780, USNM 258782 L tympanic bullae, USNM 259000 L and R tympanic bulla, USNM 25593 petrosal (juvenile), USNM 265596 R petrosal (juvenile), USNM 291101 L petrosal, USNM 291001 L petrosal, USNM 291101 L petrosal, USNM 257513 R and L petrosal; ZMUC 27[x] R tympanic bulla

Eubalaena australis – USNM 484901, USNM 484899 L bullae and petrosals (juvenile); USNM 267612 L and R tympanic bulla and petrosal with malleus, incus and stapes; SAM 18071 R tympanic bulla and petrosal

Eubalaena glacialis – AMNH 169829 L tympanic bulla and petrosal; LACM 54763 R tympanic bulla and petrosal; USNM 269161 (juvenile) L tympanic bulla, USNM 20868 (juvenile), USNM 504886 (juvenile), USNM 504343 (juvenile) tympanic bulla and petrosal, USNM 23077, USNM 504886 (juvenile) R petrosals, USNM 50860 R tympanic bulla and petrosal (fetal); ZMUC CN1 L and R tympanic bulla and L petrosal (neonate), ZMUC CN 7 R tympanic bulla (neonate), ZMUC CN 2, L tympanic bulla and petrosal, ZMUC 4 L tympanic bulla, ZMUC CN 12x, CN 5 R tympanic bulla and petrosal, ZMUC CN 35 R petrosal

Eubalaena japonica – USNM 16434 R tympanic bulla and petrosal; USNM 16435 L tympanic bulla and petrosal

Neobalaenidae

Caperea marginata – USNM 550146 L petrosal, NMV 28531 R tympanic bulla and petrosal, IRSNB 1536; SAM M9079 R tympanic bulla and petrosal; SAM 6110 L tympanic bulla and petrosal; SAM 9079 R tympanic bulla and petrosal (juvenile)

Balaenopteridae

Balaenoptera acutorostrata – AMNH 35680 L petrosal; USNM 300321 R and L tympanic bulla, USNM 314864 (neonate) R and L petrosal, USNM 504854 R and L tympanic bulla; SDSNH 23642 R tympanic bulla and petrosal; LACM 54598 R tympanic bulla, LACM 72507 L tympanic bulla and attached petrosal (neonate); ZMUC 19[X] R tympanic bulla

Balaenoptera bonaerensis – NSMT 25092 R tympanic bulla, NSMT 25924 tympanic bulla, NSMT 25919 tympanic bulla, NSMT 25921 tympanic bulla, NSMT 25922 tympanic bulla; USNM 504951 R and L tympanic bulla and petrosals, USNM 504952 L and R tympanic bulla and L petrosal, USNM 504953 R bulla and petrosal; USNM 504954 R and L tympanic bulla and petrosals, USNM 504955 R and L tympanic bulla and petrosals; SAM M11365 L tympanic bulla and petrosal (juvenile)

Balaenoptera borealis – NSMT 25924 R tympanic bulla; USNM 236680 R and L

bulla and petrosals, USNM 504244 L and R tympanic bulla, USNM 504692 R and L tympanic bulla and petrosal attached (misidentified as *B. edeni*),USNM 504698 L and R tympanic bulla and petrosal attached, USNM 504699 R and L tympanic bulla and petrosal, USNM 504700 L and R tympanic bulla and petrosal, USNM 504701 R tympanic bulla and petrosal, USNM 486174 L and R tympanic bulla, USNM 571340 L and R tympanic bulla and petrosals, USNM 571436 R petrosal and fragmentary tympanic bulla, USNM 571925 L tympanic bulla; NSMT 25908 L tympanic bulla, NSMT 25909 tympanic bulla, NSMT 25915 tympanic bulla; ZMUC 2 R tympanic bulla, ZMUC L tympanic bulla

Balaenoptera edeni – USNM 239307 R petrosal (juvenile); NSMT 2007 L tympanic bulla, NSMT 27006 tympanic bulla, NSMT 25918 tympanic bulla

Balaenoptera musculus – USNM 124326 R and L tympanic bulla, USNM 239280 R tympanic bulla and R petrosal, USNM 259329 R tympanic bulla, USNM 269540 R bulla and petrosal (neonate), USNM 268001 R tympanic bulla and petrosal (neonate); NSMT 25902 R tympanic bulla, NSMT 25900, NSMT 25901 tympanic bulla, NSMT 25902 R tympanic bulla; SDSNH 23760 L tympanic bulla; ZMUC 10, 18 L tympanic bulla; ZMUC 16 R tympanic bulla, CN 12^{x} R tympanic bulla and petrosal (neonate)

Balaenoptera omurai – NSMT 32505 R tympanic bulla, NSMT 32992 R petrosal

Balaenoptera physalus – AMNH 148407 R tympanic bulla and petrosal; NSMT 25903 tympanic bulla, NSMT 25905 tympanic bulla, NSMT 25904 L tympanic bulla; SAM 20752 R tympanic bulla and petrosal (juvenile);SDSNH 23590 petrosal, SDSU S-970 neonate L and R tympanic bulla and petrosal; USNM 16039 L and R petrosal, USNM 237566, R tympanic bulla and L petrosal, USNM 239707 R tympanic bulla and petrosal, USNM 239332 R tympanic bulla and petrosal, USNM 269155 L tympanic bulla and petrosal (neonate), USNM 269156 R tympanic bulla and petrosal (neonate), USNM 48494 R and L tympanic bulla and petrosal (juvenile), USNM 504243 L and R tympanic bulla, USNM 504258 L tympanic bulla and petrosal, USNM 504344 L tympanic bulla, USNM 504485 L and R tympanic bulla, USNM 504702 L and R tympanic bulla and petrosals, USNM 504703 L and R tympanic bulla and petrosals, USNM 504704 L and R tympanic bulla and petrosal, USNM 504709 and USNM 504711L and R tympanic bulla, USNM 550116, USNM 571916, USNM 571919, USNM 571920, R tympanic bulla, USNM 572539 R tympanic bulla and petrosal (neonate), USNM 572675, USNM 572676, USNM 572677 L tympanic bulla, USNM 572680 R tympanic bulla, USNM 572681 L and R tympanic bulla, USNM 572682 R and L tympanic bulla, USNM 572683 and USNM 572684 L tympanic bulla, USNM 572685, USNM 572686, USNM 572687, and USNM 572688 L tympanic bulla; ZMUC 17a R tympanic bulla, ZMUC 22, 27, 29, 32b L tympanic bulla

Megaptera novaeangliae – LACM 484911, LACM 52453 L tympanic bulla;

NSMT 25929, NSMT 25935, NSMT 25930, NSMT 25934, NSMT 25937, NSMT 25933, NSMT 25936 tympanic bulla, NSMT 26435 L tympanic bulla; USNM 13656 L and R tympanic bulla, USNM 237259 R and L tympanic bulla and petrosals (neonate), USNM 25300 L and R tympanic bulla, USNM 259328 R tympanic bulla, USNM 486175 R petrosal, USNM 484991 L tympanic bulla, USNM 21492 R petrosal, USNM 504216 L and R tympanic bulla, USNM 504956 R tympanic bulla; ZMUC uncatalogued R tympanic bulla, ZMUC CN 16^{x} L tympanic bulla and petrosal (neonate)

Eschrichtiidae

Eschrichtius robustus – LACM 31684 neonate R tympanic bulla and petrosal, LACM 54541 neonate L tympanic bulla and petrosal, LACM 84152, 85980, 84151, 31683, 31679, 31681 tympanic bulla; NSMT 26431 L tympanic bulla, NSMT 504305, NSMT 364973, NSMT 364971, NSMT 364975, NSMT 364970, NSMT 364979, NSMT 364972, NSMT 571931, NSMT 484991 tympanic bulla; SDSNH 23762 R and L tympanic bulla and petrosal (neonate);USNM 13803 R and L petrosal and L tympanic bulla, USNM 364969 L tympanic bulla, USNM 364970 and USNM 364971 L and R tympanic bulla; USNM 364972, USNM 364973, USNM 364974, USNM 364975, USNM 364977 L tympanic bulla, USNM 364979 R tympanic bulla, USNM 364980 R petrosal, USNM 504305 L tympanic bulla, USNM 571931 R tympanic bulla and petrosal with glove finger
