# Supplementary material for: The Comparative Osteology of the Petrotympanic Complex (Ear Region) of Extant Baleen Whales (Cetacea: Mysticeti)
Source: PLoS One. 2011 Jun 22;6(6):e21311. doi: 10.1371/journal.pone.0021311 (PMC3120854; doi:10.1371/journal.pone.0021311)
Supplement: Table S2 — Measurements (mm) of tympani bulla of balaenid and neobalaenid species. (PDF) [file pone.0021311.s004.pdf]

Table S2. Measurements (mm) of tympanic bulla of balaenid and neobalaenid species.

Specimens	Length	Width[a]	Height
Balaenidae			
Balaena mysticetus			
SDSNH 23715	139.79	110.46	80.27
USNM 12182	144.80	105.74	86.16
USNM 49407	146.70	111.67	87.86
USNM 63330	139.47	(60.66), 87.56	74.88
USNM 15595	–	97.72	82.07
USNM 259000	162.1	99.37	71.67
Eubalaena australis			
SAM 18071	133.92	108.00	93.00
USNM 26712	145.90	(93.82), 121.63	73.00
Eubalaena glacialis			
AMNH 169829	123.21	(106.00[b]), 114.11	84.96
LACM 54763	112.32	(63.67), 84.52	81.06
USNM 20868	138.11	88.56	82.16
USNM 269161	133.47	96.30	80.76
USNM 504886	123.64	(78.02), 113.53	8.68
Eubalaena japonica			
USNM 16435	161.79	(113.74), 131.01	94.49
Neobalaenidae			
Caperea marginata			
NMV 28531	107.72	86.13	–
SAM 6110	106.07	86.15	47.41

[a]width measured to conical process
[b]estimated
