# Supplementary material for: The Comparative Osteology of the Petrotympanic Complex (Ear Region) of Extant Baleen Whales (Cetacea: Mysticeti)
Source: PLoS One. 2011 Jun 22;6(6):e21311. doi: 10.1371/journal.pone.0021311 (PMC3120854; doi:10.1371/journal.pone.0021311)
Supplement: Table S3 — Measurements (mm) of tympanic bulla of balaenopterid and eschrichtiid species. (PDF) [file pone.0021311.s005.pdf]

Table S3. Measurements (mm) of tympanic bulla of balaenopterid and eschrichtiid species.

Specimen	Length	Width	Height
Balaenopteridae			
Balaenoptera acutorostrata			
LACM 54598	94.14	68.91	53.09
LACM 72507	81.19	62.88	51.80
SDSNH 23642	88.44	62.89	47.14
USNM 292055	86.99	64.88	45.16
USNM 504854	82.85	64.79	44.10
ZMUC 19[x]	78.58	62.69	41.54
Balaenoptera bonaerensis			
NSMT 25919	90.20	72.34	54.61
NSMT 25921	96.05	72.28	56.77
NSMT 25922	99.47	–	54.12
NSMT 25924	98.89	72.24	52.06
USNM 504951	94.97	73.65	–
USNM 504952	94.65	–	47.92
USNM 504953	94.11	70.32	52.59
USNM 504954	95.13	72.25	56.21
Balaenoptera borealis			
NSMT 25908	129.46	94.59	61.15
NSMT 25909	138.41	88.35	72.68
NSMT 25915	126.83	92.60	62.49
USNM 236680	121.80	87.52	62.66
USNM 486174	123.69	89.19	64.74
USNM 504244	132.49	96.31	64.46
USNM 504700	121.48	92.26	60.83
USNM 504701	124.63	92.10	65.72
USNM 504699	124.89	97.60	61.22
USNM 504698	118.75	87.99	62.02
USNM 571925	126.25	94.00	61.71
ZMUC 2	115.39	87.56	59.08
ZMUC 5	122.85	89.67	57.90
Balaenoptera edeni			
NSMT 25918	106.51	79.99	53.15
NSMT 27006	114.89	87.10	56.85
NSMT 27007	112.89	91.27	55.74
USNM 504692	124.22	85.52	66.79
Balaenoptera musculus			
NSMT 25900	146.43	102.33	78.37
NSMT 25901	133.81	93.26	68.32

NSMT 25902	140.75	106.09	76.01
NSMT 25907	137.34	103.31	75.83
SDSNH 23760	134.75	87.36	73.27
USNM 259329	138.04	105.43	74.49
ZMUC 10	136.50	97.14	73.84
ZMUC 16	136.52	104.07	71.39
ZMUC 18	134.48	102.03	77.16
Balaenoptera omurai			
NSMT 32505	122.34	85.93	57.91
Balaenoptera physalus			
AMNH 148407	119.63	92.76	68.19
NSMT 25903	133.29	93.24	70.43
NSMT 25904	130.83	97.33	70.38
NSMT 25905	126.13	91.87	66.33
SDSU-S-970	120.22	83.04	75.36
USNM 237566	127.12	88.77	67.46
USNM 239707	130.84	84.24	62.35
USNM 484994-	120.85	83.82	67.33
USNM 504243	125.10	89.99	69.38
USNM 504244	132.02	96.90	62.62
USNM 504344	115.27	80.39	69.31
USNM 504485	110.88	86.90	65.72
USNM 504703	121.81	87.76	64.98
USNM 504704	131.63	90.74	69.58
USNM 504702	123.41	84.86	66.28
USNM 550115	114.85	81.83	62.31
USNM 571916	124.39	91.11	68.95
USNM 571919	123.24	90.44	66.84
USNM 571920	127.36	91.19	71.35
ZMUC 17a	128.30	93.61	70.68
ZMUC 22	127.67	90.43	66.15
ZMUC 27	133.91	96.53	67.45
ZMUC 29	128.54	95.10	69.52
ZMUC 32b	128.80	93.89	69.95
Megaptera novaeangliae			
LACM 484911	114.38	84.74	69.09
LACM 52453	122.80	–	78.92
NSMT 25929	116.25	91.86	71.10
NSMT 25930	113.46	93.60	72.06
NSMT 25933	114.74	86.97	75.15
NSMT 25934	113.11	92.22	75.44
NSMT 25935	120.49	92.66	73.18
NSMT 25936	114.49	89.38	70.93

NSMT 25937	113.63	90.05	71.55
NSMT 26435	107.17	80.28	63.96
USNM 13656	115.85	86.31	73.37
USNM 25300	117.94	–	72.18
USNM 259328	120.81	86.60	74.76
USNM 484991	113.13	83.64	65.38
USNM 504216	116.73	87.69	75.98
USNM 504956	106.70	–	70.19
ZMUC uncat.	110.56	88.92	69.29
Eschrichtiidae			
Eschrichtius robustus			
LACM 31679	88.17	73.18	53.65
LACM 31681	81.94	71.07	47.02
LACM 31683	90.88	78.83	62.85
LACM 31684	86.56	76.48	50.33
LACM 54541	94.07	80.96	67.03
LACM 84151	98.93	86.16	56.91
LACM 84152	108.18	85.95	55.24
LACM 85980	110.67	101.41	58.78
NSMT 25431	93.02	86.67	56.48
NSMT 25898	105.20	92.55	59.67
NSMT 25899	107.28	94.24	–
NSMT 25897	106.65	93.65	59.90
NSMT 25896	107.24	98.44	62.33
NSMT 26432	103.38	90.19	57.02
SDSNH 23751	107.28	90.16	56.67
USNM 364973	105.01	89.58	57.63
USNM 364975	101.07	85.41	54.94
USNM 364970	97.38	90.23	53.22
USNM 364979	105.06	87.95	53.59
USNM 364972	110.76	91.51	58.69
USNM 504305	104.96	87.91	55.93
USNM 571931	105.62	87.09	55.18
USNM 4844991	114.97	84.99	65.88
