# Supplementary material for: The Comparative Osteology of the Petrotympanic Complex (Ear Region) of Extant Baleen Whales (Cetacea: Mysticeti)
Source: PLoS One. 2011 Jun 22;6(6):e21311. doi: 10.1371/journal.pone.0021311 (PMC3120854; doi:10.1371/journal.pone.0021311)
Supplement: Table S4 — Tympanic bulla anterior lobe measurements (mm) among mysticetes. (PDF) [file pone.0021311.s006.pdf]

Table S4. Tympanic bulla anterior lobe measurements (mm) among mysticetes.

Specimen	Lobe Length	Lobe/Bulla Length[a] (%)
Balaenidae		
Balaena mysticetus		
SDSNH 23715	46.48	47
Eubalaena glacialis		
LACM 169829	37.24	30
LACM 54763	32.64	29
Balaenopteridae		
Balaenoptera acutorostrata		
LACM 72507	35.08	43
LACM 54598	94.14	45
SDSNH 23642	88.44	51
Balaenoptera bonaerensis		
USNM 504952	40.7	43
USNM 504953	44.8	48
Balaenoptera borealis		
USNM 504698	50.37	42
Balaenoptera edeni		
USNM 504692	55.55	45
Balaenoptera musculus		
SDSNH 23760	53.16	40
USNM 259329	56.34	41
Balaenoptera physauls		
AMNH 148407	48.72	41
SDSU S-970	49.13	41
Megaptera novaeangliae		
LACM 52453	57.34	47
USNM 484911	51.89	45
Eschrichtiidae		
Eschrichtius robustus		
LACM 31684	36.62	42
LACM 54541	39.64	42
SDSNH 23761	51.53	48
SDSNH 23762	94.72	47

[a]Bulla length measurements used are listed in Tables 2-5.
