# Supplementary material for: The Comparative Osteology of the Petrotympanic Complex (Ear Region) of Extant Baleen Whales (Cetacea: Mysticeti)
Source: PLoS One. 2011 Jun 22;6(6):e21311. doi: 10.1371/journal.pone.0021311 (PMC3120854; doi:10.1371/journal.pone.0021311)
Supplement: Table S5 — Tympanic bulla conical process measurements (mm) among mysticetes. (PDF) [file pone.0021311.s007.pdf]

Table S5. Tympanic bulla conical process measurements (mm) among mysticetes.

Specimen	Length	Height	Height/Length (%)
Balaenidae			
Eubalaena glacialis			
AMNH 169829	26.09	6.98	27
Balaenopteridae			
Balaenoptera acutorostrata			
LACM 54598	18.23	6.13	34
ZMUC 19[x]	17.99	6.53	36
Balaenoptera bonaerensis			
USNM 504953	14.96	7.11	46
USNM 504952	18.13	7.35	41
Balaenoptera borealis			
USNM 504698	19.68	7.06	36
ZMUC 2	19.34	4.59	24
ZMUC 5	16.28	5.97	37
Balaenoptera edeni			
USNM 504692	21.34	8.11	38
Balaenoptera musculus			
USNM 259329	29.36	8.49	29
ZMUC 10	21.16	5.98	28
ZMUC 16	25.82	6.78	26
ZMUC 18	25.55	7.79	30
Balaenoptera physalus			
AMNH 148407	23.88	11.39	48
ZMUC 17a	22.73	11.03	49
ZMUC 22	19.4	11.62	60
ZMUC 27	21.82	9.67	44
ZMUC 29	22.76	11.4	51
Megaptera novaeangliae			
USNM 484991	16.24	7.4	46
Eschrichtiidae			
Eschrichtius robustus			
SDSNH 23762	22.16	10.13	46
