# Supplementary material for: The Comparative Osteology of the Petrotympanic Complex (Ear Region) of Extant Baleen Whales (Cetacea: Mysticeti)
Source: PLoS One. 2011 Jun 22;6(6):e21311. doi: 10.1371/journal.pone.0021311 (PMC3120854; doi:10.1371/journal.pone.0021311)
Supplement: Table S6 — Petrosal measurements (mm) among mysticetes and reated taxa. (PDF) [file pone.0021311.s008.pdf]

Table S6. Petrosal measurements (mm) among mysticetes and related taxa.

Specimen	PC Length	AP Length	PC/AP (%)
Balaenidae			
Eubalaena glacialis			
AMNH 169829	51.6	87.64	59
Balaenopteridae			
Balaenoptera acutorostrata			
USNM 35680	52.23	74.46	70
Balaenoptera bonaerensis			
USNM 504952	66.45	98.14	68
Balaenoptera borealis			
USNM 504698	58.98	129.58e	46
Balaenoptera edeni			
USNM 504692	65.17	128.57	51
Balaenoptera musculus			
USNM 239280	69.76	112.64	62
Balaenoptera physalus			
AMNH 148407	65.15	185.00e	35
Megaptera novaeangliae			
USNM 486175	70.62	103.52	68
Eschrichtiidae			
Eschrichtius robustus			
SDSNH 23762	59.61	56.3	106

Abbreviations: AP=anterior process, e= estimated, PC=pars cochlearis.
