# Supplementary material for: The Comparative Osteology of the Petrotympanic Complex (Ear Region) of Extant Baleen Whales (Cetacea: Mysticeti)
Source: PLoS One. 2011 Jun 22;6(6):e21311. doi: 10.1371/journal.pone.0021311 (PMC3120854; doi:10.1371/journal.pone.0021311)
Supplement: Table S7 — Data matrix of petrotrympanic characters scored for extant mysticetes used in phylogeneti analyses. Matrix can be downloaded from project page associated with this manuscript at MorphoBank (www.morphobank.org). (PDF) [file pone.0021311.s009.pdf]

Table S7. Data matrix of petrotympanic characters scored for extant mysticetes used in phylogenetic analyses. Matrix can be downloaded from project page associated with this manuscript at MorphoBank (www.morphobank.org).

```
                                     1 1111111112 2222222223 3333333334 44444444
Taxon/Node                  1234567890 1234567890 1234567890 1234567890 12345678
--------------------------------------------------------------------------------
Mammalodon colliveri        0000000??0 0?0????00 0000?0011? ?01010?0?0 000??000
Eomysticetus whitmorei      000?000??0 00????000 00?000011? 0100010010 ??00?000
Balaenoptera acutorostrata  0011011110 0100211121 1010011200 0101001010 00110111
Balaenoptera bonaerensis    0011011210 0100011121 1010011200 0110101111 00110101
Balaenoptera borealis       0011011110 0000010121 0010011200 0110001111 11112101
                                              2   1
Balaenoptera edeni          0011011210 0000010121 0010011200 0110001110 01112111
Balaenoptera musculus       0011011111 1010010201 0010011200 1111001111 01110011
                                1
Balaenoptera omurai         0011011?10 0000010111 0110011100 011???1010 ??110101
Balaenoptera physalus       0011011210 0010010201 0010011200 11110011?1 0011?011
                                       1          1           2
Megaptera novaeangliae      0011011210 0000200112 0010011200 00110111?0 11121001
                                1      1
Eschrichtius robustus       0011000200 0000000112 0000021202 0201000100 11201121
                                                                 1
Caperea marginata           1011000020 0001010003 001011011? 010?101010 0?20?010
Balaena mysticetus          1111000020 0001100310 0011110111 0010011020 01200002
                                 1
Eubalaena sp.               1111000020 0001200300 0011110111 0010011020 11200002
                                 1
```
