# Supplementary material for: The Comparative Osteology of the Petrotympanic Complex (Ear Region) of Extant Baleen Whales (Cetacea: Mysticeti)
Source: PLoS One. 2011 Jun 22;6(6):e21311. doi: 10.1371/journal.pone.0021311 (PMC3120854; doi:10.1371/journal.pone.0021311)
Supplement: Text S1 — Dichotomous key for identifying extant species of mysticetes using the petrotympanic complex. (PDF) [file pone.0021311.s010.pdf]

Text S1. Dichotomous key for identifying extant species of mysticetes using the petrotympanic complex.

1a. Main ridge of tympanic bulla is short and confined to posterior end…**Odontoceti**

1b. Main ridge is long and extends to the anterior end of the bulla…**2** (Mysticeti)

2a(1b). Tympannic bulla is rhomboid and dorsoventrally compressed, anterior lobe of bulla short, posterior process of petrosal at a right angle to pars cochlearis…**3** (Balaenoidea)

2b. Tympanic bulla is ovoid and convex, anterior lobe of bulla is long, posterior process of petrosal extends posterolaterally from pars cochlearis…**5** (Balaenopteroidea)

3a(2a). Apex of anterior process elongated and inflated, perilymphatic foramen narrowly separated from fenestra cochleae…***Caperea marginata***

3b. Apex of anterior process rounded, perilymphatic foramen is separated from fenestra cochleae, lateral projection is hypertrophied…**4** (Balaenidae)

4a(3b). Main and involucral ridges converge at posterior end, stapedial fossa and facial nerve sulcus separated be a thin ridge…***Balaena mysticetus***

4b. Main and involucral ridges converge at anterior end, stapedial fossa confluent with facial nerve sulcus…***Eubalaena* sp.**

5a(2b). Fenestra cochleae significantly larger than fenestra vestibuli, suprameatal area of petrosal flat or nearly flat…***Megaptera novaengliae***

5b. Size of fenestra cochleae equal to or smaller than fenestra vestibuli, suprameatal area bulbous or rugose…**6** (*Balaenoptera* or *Eschrichtius*)

6a(5b). Involucral and main ridges at same approximate elevation, no anterolateral ridge or shelf, conical process thick, anterior process bluntly triangular, anteroexternal sulcus may be present on anterior process, medially directed process extending ventrally from promotorium anterior to fenestra vestibuli, posterior cochlear crest developed as a ventrally directed process…***Eschrichtius robustus***

6b. Involucral and main ridges not coincident, anterior ridge or shelf present on tympanic bulla, anteroexternal sulcus never present, apex of anterior process narrowly triangular…**7** (*Balaenoptera*)

7a(6b). Promontorium rounded and convex ventrally…**8**

7b. Promontorium broad and flattened ventrally…**9**

8a(7a). Bulla exends posteriorly from end of tympanic cavity, pars cochlearis anteroposterior length less than anterior process length (45-70%), perilymphatic foramen well separated from fenestra cochleae…***Balaenoptera musculus***

8b. Posterior border of bull adjacent to opening of tympanic cavity, pars cochlearis longer than anterior process (>100%), perilymphatic foramen narrowly separated or confluent with fenestra cochleae…***Balaenoptera physalus***

9a(7b). Thin bony projections extending from anterior border of petrosal, anterior process not strongly attached to promontorium…***Balaenoptera omurai***

9b. Anterior border of petrosal free from thin bony projections, anterior process strongly attached to promontorium…**10**

10a(9b). Stapedial fossa and facial nerve sulcus confluent, internal opening of facial nerve canal circular but without a crista transversa reaching the cerebellar surface of the petrosal…***Balaenoptera borealis***

10b. Stapedial fossa and facial nerve sulcus separated by thin ridge, internal opening of facial canal oval or circular with crista transversa reaching cerebellar surface…**11**

11a(10b). Dorsal surface of involucrum near Eustachian notch rounded, anterointernal sulcus absent…***Balaenoptera edeni***

11b. Dorsal surface of involucrum planar adjacent to Eustachian notch, anterointernal sulcus present on anteromedial surface of anterior process…**12**

12a(11b). Crista transversa low and does not reach cerebellar surface, involucral and main ridges converge…***Balaenoptera acutorostrata***

12b. Crista transversa high and reaches cerebellar surface, involucral and main ridges of bulla extend parallel to each other…***Balaenoptera bonaerensis***
