# Supplementary material for: The Comparative Osteology of the Petrotympanic Complex (Ear Region) of Extant Baleen Whales (Cetacea: Mysticeti)
Source: PLoS One. 2011 Jun 22;6(6):e21311. doi: 10.1371/journal.pone.0021311 (PMC3120854; doi:10.1371/journal.pone.0021311)
Supplement: Text S2 — Phylogenetic characters derived from the petrotympanic complex of extant mysticetes. Further information can be found on the project page associated with this study at MorphoBank (www.morphobank.org). (PDF) [file pone.0021311.s011.pdf]

Text S2. Phylogenetic characters derived from the petrotympanic complex of extant mysticetes. Further information can be found on the project page associated with this study at MorphoBank (www.morphobank.org).

Character 1– Tympanic bulla, shape.
STATES: 0=ovoid; 1=rhomboid.

DESCRIPTION: The shape of the tympanic bulla has been used to distinguish various clades of mysticetes [16, 92], and bulla shape has been used in phylogenetic analyses (e.g., Steeman who described the 'rhomboid' state used here as "box shaped and elongate" [73], character 49, p. 892; Geisler and Sanders who described the shape as "narrow and long" versus "wide" [49], character 251, p. 120). In the plesiomorphic state, the tympanic bulla is ovoid and kidney-shaped, where the medial border curves continuously in ventral view. In the apomorphic state, the anterior edge of the main ridge has a steeper slope, giving the anterior region of the bulla a flattened appearance in ventral view with a distinct anteromedial corner. The breadth of the anteromedial expansion of the bulla that forms the anteromedial corner appears to increase with ontogenetic age (personal observation), whereas more mature individuals tend to have a better developed anteromedial corner (although overall length of the bulla does not appear to increase with ontogenetic age in *Balaenoptera acutorostrata*, as reported by Oishi and Hasegawa ([16], fig. 2). The 'rhomboid' condition also can be expressed as the maximum transverse width (perpendicular to the longitudinal axis) at the anterior end of the bulla, rather than posterior to, or at the level of, the sigmoid process.

TAXONOMIC DISTRIBUTION: The bulla is ovoid (plesiomorphic condition) in *Eomysticetus whitmorei*, *Eschrichtius robustus*, and all extant balaenopterids. Balaenids and *Caperea marginata* are characterized by having the apomorphic rhomboid condition. The anterior border of the bulla of *Caperea* indeed is flattened with an angle at the anteromedial corner, although it is not as well defined as in balaenids. Nonetheless, we consider *Caperea* to possess the derived condition for the shape of the tympanic bulla.

HISTORICAL USAGE: This character was based on previously described morphology [5, 16, 92] and was previously used in phylogenetic analyses ([24], character 113; [49], character 251; [73], characters 49 and 51).

Character 2– Tympanic bulla, dorsoventral compression.
STATES: 0=absent; 1=present.

DESCRIPTION: Dorsoventral compression of the medial half of the tympanic bulla was described by Geisler and Sanders [49] as a concavity in the profile of the tympanic bulla. Although Geisler and Sanders focused their study on the phylogeny of odontocetes, and their character 268 is similar to our character, the compression and associated concavity observed on the medial aspect of the ventral surface of balaenids may not be homologous between baleen and toothed whales. The bulla is inflated in the plesiomorphic state, but the dorsoventral compression of the bulla characterizes the apomorphic state of this character. The concavity produced by the compression is on the ventral surface of the bulla, extending longitudinally.

TAXONOMIC DISTRIBUTION: The plesiomorphic state is observed in most cetaceans, and the typical mammalian condition for the tympanic bulla. Dorsoventral compression (state 1) is observed only in balaenids among extant mysticetes.

HISTORICAL USAGE: This character was based on previously described morphology and a phylogram ([81], character suite 8), and it was previously used in phylogenetic analyses ([21], character 34; [22], character 31; [23], character 119; [49], character 268; [60], character 44; [69], character 47; [70], character 104; [73], character 53; [82], character 26).

Character 3– Tympanic bulla, main ridge.
STATES: 0=short, confined to posterior end; 1=long, extends to anterior end.

DESCRIPTION: The term 'main ridge' of the tympanic bulla (referred to as the median keel by Luo [10], table 2]) was defined by Oishi and Hasegawa ([16], fig. 3), but the morphology of the ridge has received little to no treatment in phylogenetic analyses. The main ridge gives the medial border of the tympanic bulla a prominent edge in many taxa, including *Balaenoptera physalus*. In the plesiomorphic state, the main ridge is short and restricted to the posterior end of the bulla. In the apomorphic state, the main ridge is long and extends from the posterior to anterior ends of the bulla. There is some variation in the main ridge in terms of degree of dorsoventral thickness, and its edge is either bluntly rounded (e.g., *Balaenoptera musculus*) or keeled (e.g., *Balaenoptera bonaerensis*), and the main ridge is indistinct but nonetheless present in *Megaptera novaeangliae*. In certain taxa, the main ridge is extended posteriorly to form a type of outer posterior prominence (e.g., *Megaptera*), but it is unclear whether or not this projection is homologous with the outer posterior prominence of odontocetes (e.g., *Tursiops truncatus* [25]).

TAXONOMIC DISTRIBUTION: The plesiomorphic state of a short main ridge is seen in extinct mysticetes, such as *Eomysticetus whitmorei* and *Mammalodon colliveri*. The main ridge is long (state 1) in all extant mysticetes.

HISTORICAL USAGE: This character has not been previously used in phylogenetic analyses.

Character 4– Tympanic bulla, median furrow.
STATES: 0= present; 1=absent.

DESCRIPTION: The median furrow is a longitudinal sulcus that extends anteriorly from the interprominential notch (between the inner and outer posterior prominences of the bulla [24], fig. 28; [25], fig. 25) on the posterior border of the bulla in many cetaceans ([5], [25], p. 141). The furrow is homologous with several structures identified by previous researchers, including the ventral groove [22, 67, 93], posterior cleft [94], and deep median hollow [18]. We prefer the term median furrow, following the terminology used in the landmark descriptions by Kasuya ([5], fig. 1), as well as by Mead and Fordyce ([25], p. 141), as it complements the term 'lateral furrow', which commonly is used to refer to a distinct mediolaterally directed groove anterior to the sigmoid process

on the lateral side of the ventral surface of the bulla ([16], fig. 3). The median furrow is well-developed but short in the plesiomorphic state, occupying the posterior 40% of the bulla and forming the median border of broadly rounded inner (medial and outer (lateral) posterior prominences. The median furrow is lost all together in the apomorphic state.

TAXONOMIC DISTRIBUTION: The plesiomorphic condition is observed in some extinct mysticetes including *Mammalodon colliveri* and *Eomysticetus whitmorei*. The furrow is altogether absent (apomorphic state) in extant mysticete species.

HISTORICAL USAGE: This character was based on described morphology ([93], fig. 4), and was previously used in phylogenetic analyses ([2], character 41; [22], character 32; [23], character 118; [24], character 116; [49], character 266; [60], character 45; [67], character 59; [68], character 18; [69], character 49; [70], character 100; [82], character 27; [95], character 14).

Character 5– Tympanic bulla, dorsal posterior prominence.
STATES: 0=absent or weakly developed; 1=conspicuous.

DESCRIPTION: The dorsal posterior prominence is a raised swelling on the posterior portion of the dorsal surface of the involucrum (not to be confused with either the inner or outer posterior prominences of the odontocete bulla [25]). The apomorphic state is the presence of a conspicuous dorsal posterior prominence, which is absent in the plesiomorphic condition.

TAXONOMIC DISTRIBUTION: Oishi and Hasegawa [16] identified the dorsal posterior prominence in *Megaptera novaeangliae* and several extinct taxa, but we observed variation in the development of the structure in this species, as well as in *Balaenoptera musculus*. Absence of significant swelling in the posterodorsal region of the bulla (the plesiomorphic condition) characterizes the remaining mysticetes, in which the dorsal aspect of the involucrum is flattened with no anterior-posterior swelling in the area.

HISTORICAL USAGE: Presence versus absence of the dorsal posterior prominence was used in at least one study focused on mysticete systematics ([16], character 6, fig. 4), although it was not used in a phylogenetic analysis.

Character 6– Tympanic bulla, involucral ridge and main ridge.
STATES: 0=involucral ridge coincident with medial edge (main ridge); 1=involucral ridge laterally retracted.

DESCRIPTION: In the plesiomorphic state, the involucral ridge extends to the medial border of the bulla, which is formed by the main ridge, when the bulla is in dorsal view ([16] fig. 3). The involucral ridge is laterally retracted from the main ridge and does not extend to the medial border of the bulla in the apomorphic state.

TAXONOMIC DISTRIBUTION: The plesiomorphic state characterizes *Eomysticetus whitmorei*, *Mammalodon colliveri*, *Eschrichtius robustus*, and the balaenoids (*Balaena*, *Eubalaena*, and *Caperea*). The apomorphic state is characteristic of extant species of

Balaenopteridae. However, the morphology is polymorphic for some mysticetes. For example, bullae of *Balaena mysticetus* exhibit both the ancestral (USNM 15695, 259000) and derived (USNM 49407, 63300) conditions. Likewise, we observed the apomorphic state in some specimens of *Eubalaena glacialis* (AMNH 169829, 267612, 504343, 504886), and the plesiomorphic state is observed in others (USNM 269161).

HISTORICAL USAGE: This character has not been previously used in phylogenetic analyses.

Character 7– Tympanic bulla, anterolateral ridge or shelf.
STATES: 0=absent; 1=present.

DESCRIPTION: A ridge or shelf-like extension is located on the ventral surface of the tympanic bulla at the anterolateral end, adjacent to the anterior pedicle in some extant mysticetes. The shelf is recognizable as a slight concavity at the anterolateral corner of the bulla in ventral view (apomorphic state), and the absence of the structure is the plesiomorphic state that characterizes the anterior border of the bullae of most cetaceans and non-cetacean mammals. Development of the shelf ventral to the opening for the Eustachian tube varies among taxa, ranging from a ridge (e.g., *Balaenoptera acutorostrata*) to a well-developed shelf (e.g., *Balaenoptera borealis*).

TAXONOMIC DISTRIBUTION: The plesiomorphic state characterizes the majority of extant mysticetes. However, the apomorphic state in which the anterolateral shelf is present on the bullae of balaenopterids is weakly developed in *Megaptera novaeangliae* and *Balaenoptera musculus*.

HISTORICAL USAGE: This character has not been previously used in phylogenetic analyses.

Character 8– Tympanic bulla, conical process height in proportion to length.
STATES: 0=weakly developed or absent; 1=short, height between 27-36% of length; 2=tall, height between 41-48% of length.

DESCRIPTION: The conical process (often referred to as the conical apophysis or middle conical process ([2], p. 36; [10], p. 277; [18], p. 113; [25], p. 136) is a raised projection along the lateral edge of the tympanic bulla, immediately posterior to the sigmoid process. Both the sigmoid and conical processes are homologous to processes on the tympanic ring of non-cetacean mammals ([25], p. 136). The conical process serves as the distal attachment for the conical tympanic ligament [10]. The conical tympanic ligament may be homologous to all [10, 96] or part [4] of the planar tympanic membrane of terrestrial mammals. The proximal end of the conical tympanic ligament originates on the manubrium of the malleus [10]. The height of the conical process was measured as the greatest distance between the apex and base of the process, where the base was determined to be at the level of the lateral lip of the tympanic bulla, as viewed ventrally. The length of the conical process was measured as the greatest distance perpendicular to the height (Table S5). In the plesiomorphic state, the conical process is short (<36% of length). In apomorphic state 1, the conical process is tall (41-48% of

length), and a very tall conical process (>50% of length) characterizes apomorphic state 2.

TAXONOMIC DISTRIBUTION: The conical process is weakly developed or absent (plesiomorphic state) in extant balaenids and the neobalaenid *Caperea marginata*, but the structure is developed as an distinctly arched bullar feature in all other extant mysticetes. The process is short (apomorphic state 1) in *Balaenoptera acutorostrata*, *Balaenoptera borealis*, and *Balaenoptera musculus*, but the process is tall (apomorphic state 2) in all remaining balaenopteroid taxa, including *Eschrichtius robustus*. We were unable to measure the conical processes of *Mammalodon colliveri* and *Eomysticetus whitmorei*.

HISTORICAL USAGE: This character has not been previously used in phylogenetic analyses.

Character 9– Tympanic bulla, conical process thickness.
STATES: 0=thick; 1=thin anterior lip; 2=uniformly thin.

DESRIPTION: The conical process is situated on the lateral lip of the tympanic bulla, immediately posterior to the sigmoid process, and the dorsoventral thickness of the process varies among mysticetes. In the plesiomorphic state, the conical process is thick, and is developed as a globular structure with a rounded anterior end. In apomorphic state 1, the anterior lip of the conical process is thin, giving the crest of the conical process a roughly triangular shape in dorsal view. The conical process is uniformly thin across its length in apomorphic state 2.

TAXONOMIC DISTRIBUTION: The plesiomorphic state is observed in *Eschrichtius robustus*, as well as in several archaeocetes and odontocetes (personal observation). Apomorphic state 1, in which the anterior lip of the conical process is thin, is observed in extant balaenopterids, while extant balaenoids are characterized by apomorphic state 2. In *Megaptera novaeangliae*, the posterior portion of the conical process is flattened laterally.

HISTORICAL USAGE: This character has not been previously used in phylogenetic analyses.

Character 10– Tympanic bulla, posterior extension.
STATES: 0=absent; 1=present.

DESCRIPTION: The position of the sigmoid and conical processes relative to the longitudinal axis of the tympanic bulla in lateral view varies among extant mysticetes. In the plesiomorphic state, the sigmoid and conical processes are positioned at or near the posterior one-third of the longitudinal axis of the bulla, and the posterior border of the bulla is near the posterior termination of the opening (ostium) of the tympanic cavity. In the apomorphic state, the bulla extends posteriorly from the ostium for a considerable distance, which places the sigmoid and conical processes at or near the midpoint of the longitudinal axis of the bulla (note that this is the case when considering the anterior-posterior length of the bulla as a whole, and not the opening of the tympanic cavity).

TAXONOMIC DISTRIBUTION: The plesiomorphic condition is observed in most extant mysticetes, except for *Balaenoptera musculus*, which exhibits the apomorphic condition.

HISTORICAL USAGE: This character has not been previously used in phylogenetic analyses.

Character 11– Tympanic bulla, dorsal involucral surface in medial view.
STATES: 0=relatively straight or slightly curved; 1=markedly sinuous and strongly concave.

DESCRIPTION: The dorsal involucral profile in medial view presents a straight or slightly curved margin at the posterior end of the bulla in several mysticetes, which is identified as the plesiomorphic state. In the apomorphic state, the dorsal involucral surface presents a sinuous to strongly concave margin.

TAXONOMIC DISTRIBUTION: The plesiomorphic condition is seen in the outgroups *Eomysticetus whitmorei* and *Mammalodon colliveri*, as well as the extant mysticetes *Balaenoptera acutorostrata*, *Balaenoptera bonaerensis*, *Balaenoptera omurai* and balaenoids. The apomorphic state of a markedly sinuous or concave involucral surface characterizes *Balaenoptera musculus* and variably in *Balaenoptera physalus* and *Megaptera novaeangliae*.

HISTORICAL USAGE: This character has not been previously used in phylogenetic analyses.

Character 12– Tympanic bulla, dorsal surface of the involucrum adjacent to the Eustachian opening.
STATES: 0=convex; 1=planar.

DESCRIPTION: The surface of the involucrum may be either convex or planar near the opening for the Eustachian tube on the dorsal side of the tympanic bulla. In the plesiomorpic state, the surface of the involucrum adjacent to the Eustachian opening is convex, both in medial-lateral and anterior-posterior directions. Conversely, the surface is planar and almost concave, primarily in the anterior-posterior direction, in the apomorphic state.

TAXONOMIC DISTRIBUTION: Most mysticetes exhibit the pelsiomorphic state, but the apomorphic state was observed in *Balaenoptera acutorostrata* and *Balaenoptera bonaerensis*.

HISTORICAL USAGE: This character has not been previously used in phylogenetic analyses.

Character 13– Tympanic bulla, elevated and flattened posterior portion of medial margin of main ridge.
STATES: 0=absent; 1=present.

DESCRIPTION: The morphology of the main ridge of the tympanic bulla varies among mysticete taxa, not only in its anterior-posterior development, but also in its development as either a rounded ridge or a medial keel along its complete path. In some taxa, the main ridge is further modified and flattened medially, particularly at the posterior end of the ridge (apomorphic state). In the plesiomorphic state, the posterior portion of the medial margin of the bulla is gently rounded and not developed into a flattened margin.

TAXONOMIC DISTRIBUTION: The plesiomorphic state is observed in most mysticetes. An elevated and flattened medial margin (apomorphic state) is present in *Balaenoptera musculus* and *Balaenoptera physalus*, particularly at the posterior end, where the main ridge is developed as an elongate plateau. However, the character is variably developed in those taxa and may be age-related as to robustness of flattening.

HISTORICAL USAGE: This character has not been previously used in phylogenetic analyses.

Character 14– Tympanic bulla, length of anterior lobe.
STATES: 0=long, 40% or greater than total length of bulla; 1=short, 30% or less than total length of bulla.

DESCRIPTION: The anterior lobe of the tympanic bulla is the ventral swelling of the bulla anterior to the sigmoid process. The length of the anterior lobe is measured as the greatest distance (perpendicular to the lateral furrow) from the base of the trough formed by the lateral furrow to the anteriormost point of the bulla. The total length of the bulla is the greatest anterior-posterior distance along the long axis of the bulla (Table S4). The anterior lobe contributes 40% or more to the total length of the tympanic bulla in the plesiomorphic condition, but the lobe is shortened, contributing less than 30% to the total bullar length in the apomorphic state.

TAXONOMIC DISTRIBUTION: Most cetaceans possess long anterior lobes of the bullae (plesiomorphic state), although the anterior lobe is shortened in extant balaenids and neobalaenids (apomorphic state). The anterior lobe of *Balaenoptera musculus* is relatively shorter than that of other balaenopterids owing to the posterior extension of the bulla behind the ostium of the tympanic cavity, but the lobe is not reduced in *B. musculus* to the same degree as in balaenoids.

HISTORICAL USAGE: This character has not been previously used in phylogenetic analyses.

Character 15– Tympanic bulla, main ridge and involucral ridge.
STATES: 0=extend parallel with one another; 1=convergent at posterior end; 2=convergent at anterior end.

DESCRIPTION: Convergence of the main and involucral ridges along the mediodorsal edge of the tympanic bulla characterizes different mysticete taxa [16]. In the plesiomorphic state, the main and the involucral ridges are parallel along their respective courses. However, the two ridges converge at either the posterior (apomorphic state 1), or the anterior end of the bulla (apomorphic state 2).

TAXONOMIC DISTRIBUTION: The plesiomorphic state is observed in *Eschrichtius robustus*, *Balaenoptera musculus*, and *Balaenoptera physalus*, where the involucral and main ridges are parallel to one another along the length of the bulla. Apomorphic state 1 is observed in *Balaena mysticetus*, and apomorphic state 2 is observed in *Balaenoptera acutorostrata* and *Megaptera novaeangliae*.

HISTORICAL USAGE: This character was based on morphology described by Oishi and Hasegawa ([16], character 4), but it was not previously used in phylogenetic analyses.

Character 16– Tympanic bulla, involucral ridge extends to posterior end of bulla.
STATES: 0=absent; 1=present.

DESCRIPTION: In the plesiomorphic state, the involucral ridge is retracted from the posterior end of the bulla, and the main ridge continues posteriorly. In the apomorphic state, the involucral ridge curves along the posterior edge of the bulla.

TAXONOMIC DISTRIBUTION: The pleisomorphic state of the anterior retraction of the involucral ridge from the posterior border is exhibited by *Balaena mysticetus*, *Eschrichtius robustus*, and *Megaptera novaeangliae*, among extant mysticetes. The retraction of the involucral ridge in *Megaptera* is not as pronounced as that observed in *Eschrichtius*, but it does not contact the posterior border of the bulla. The involucral ridge extends to the posterior end of the bulla (apomorphic state 1) in extant species of *Balaenoptera*.

HISTORICAL USAGE: This character was based on previously described morphology ([16], character 5), but it has not been previously used in phylogenetic analyses.

Character 17– Tympanic bulla, involucral ridge extends further anterior than main ridge.
STATES: 0=absent; 1=present.

DESCRIPTION: The main ridge forms the medial border of the tympanic bulla in mysticetes, but the anterior extent of the ridge is variable among taxa. In the plesiomorphic state, the main ridge forms the anterior border of the tympanic bulla. Conversely in taxa exhibiting the apomorphic state, the involucral ridge forms the anterior border, extending further anterior than the main ridge.

TAXONOMIC DISTRIBUTION: The ancestral condition is characteristic of most mysticetes. However, *Balaenoptera acutorostrata* and *Balaenoptera bonaerensis* exhibit the apomorphic state.

HISTORICAL USAGE: This character has not been previously used in phylogenetic analyses.

Character 18– Petrosal, flange (lateral projection) of the ventrolateral tuberosity of the anterior process.
STATES: 0=absent; 1=present and small; 2=present and large; 3=present and hypertrophied.

DESCRIPTION: In mysticetes, the lateral aspect of the anterior process of the petrosal is expanded into a distinct extension lateral to the anterior pedicle for the tympanic bulla. The lateral extension in mysticetes is in a similar position to the ventrolateral tuberosity of odontocetes, and the two structures likely are homologous ([49], p. 53-54]); however, see character discussions by Geisler and Luo [11]. In the plesiomorphic state, the ventrolateral tuberosity of the anterior process is rounded and featureless. In apomorphic state 1, a small flange (homologous to the lateral projection of Geisler and Luo [11]) is present on the ventrolateral tuberosity, forming a horizontal shelf on the anterior process. The flange is large in apomorphic state 2, and it is developed as a hypertrophied structure in apomorphic state 3.

TAXONOMIC DISTRIBUTION: The plesiomorphic state is exhibited by *Caperea marginata* among extant mysticetes, and apomorphic state 1 characterizes most baleen whales. The flange of the ventrolateral tuberosity is large in *Balaenoptera physalus* and *Balaenoptera musculus*, but the size of the flange is variable in *Balaenoptera borealis*. In balaenids, the flange of the ventrolateral tuberosity is hypertrophied, often extending posteriorly to nearly the same length as the posterior process of the petrosal. A portion of the lateral tuberosity is preserved for *Mammalodon colliveri* ([24], fig. 23), but the structure is damaged so that the presence or full extent of a flange cannot be determined.

HISTORICAL USAGE: This character was previously used in phylogenetic analyses ([11], character 4; [21], character 53; [22], character 54; [23], character 89; [24], character 85; [49], characters 210-211; [59], character 4; [60], character 47; [69], character 51; [70], character 86; [72], character 201; [73], character 28; [97]; [98], character 55).

Character 19– Petrosal, anterior process attached to promontorium.
STATES: 0=absent; 1=present with an embayment; 2=present, broadly attached (no embayment).

DESCRIPTION: The anterior process of the petrosal is a neomorphic structure for Cetacea. In the plesiomorphic state, the anterior process is separated from the apex of the promontorium by a noticeable gap. In apomorphic state 1, the anterior process is connected to the promontorium, but the anterior process does not contact the rostral apex of the promontorium. Rather, there is an embayment between the two structures. There is a broader area of attachment between the anterior process and the promontorium in apomorphic state 2, where there is a continuous profile between the medial border of the anterior process and the promontorium, without an embayment as observed in state 1.

TAXONOMIC DISTRIBUTION: The plesiomorphic state characterizes the extinct taxa *Mammalodon colliveri* and *Eomysticetus whitmorei*, as well as the extant mysticetes *Balaenoptera musculus*, *Balaenoptera physalus*, *Eubalaena glacialis*, and *Balaena mysticetus*. We observed apomorphic state 1 in *Balaenoptera omurai*, *Megaptera novaeangliae*, and *Eschrichtius robustus*, in which there is an embayment between the anterior process and the rostral apex of the promontorium. The anterior edge of the anterior process is convex in ventral view in apomorphic state 2, which we observed in *Balaenoptera acutorostrata*, *Balaenoptera borealis*, and *Balaenoptera edeni*.

HISTORICAL USAGE: This character has not been previously used in phylogenetic analyses.

Character 20– Petrosal, shape of anterior process apex.
STATES: 0= rounded; 1=narrowly triangular; 2=bluntly triangular; 3=elongated and inflated.

DESCRIPTION: The shape of the apex of the anterior process of the petrosal is diagnostic for several different groups of mysticetes. In the plesiomorphic state, the apex of the anterior process is rounded. However, the anterior process assumes a triangular shape with either a narrow (apomorphic state 1) or blunt apex (apomorphic state 2) in many extant species. A third derived condition (apomorphic state 3) is an elongated and inflated anterior process.

TAXONOMIC DISTRIBUTION: We observed the plesiomorphic state in extant balaenids. The apex of the anterior process is narrowly triangular (apomorphic state 1) in several balaenopterids, including *Balaenoptera physalus*, *Balaenoptera edeni*, and *Balaenoptera borealis*, and the anterior process is triangular in *Megaptera novaeangliae* and *Eschrichtius robustus*, although the apex is blunt compared to many species of *Balaenoptera* (apomorphic state 2). *Caperea marginata* is unique among extant mysticetes in that the anterior process of this species is elongated and inflated (apomorphic state 3).

HISTORICAL USAGE: This character was previously used in phylogenetic analyses ([2], character 11; [9], character 12; [23], character 89; [49], character 201).

Character 21– Petrosal, anterointernal sulcus.
STATES: 0= absent; 1= present, located on anteromedial edge of anterior process.

DESCRIPTION: A groove is observed extending along the anteromedial edge of the anterior process and terminating at the foramen ovale in the articulated skulls of some species of *Balaenoptera* (e.g., *B. acutorostrata* and *B. physalus*), which we interpret to be the anterointernal sulcus. Absence of the sulcus is the plesiomorphic state, and presence characterizes apomorphic state 1. An anteroexternal sulcus has been described for other cetaceans ([49], [67], p. 161, fig. 11), including *Mammalodon colliveri* ([24], p. 33-36, fig. 23-26). In this toothed mysticete, the anteroexternal sulcus is directed towards the foramen ovale (although it is unclear from published descriptions if it leads directly to the opening [24]), which has been interpreted to carry the middle meningeal artery in life. The middle meningeal artery exits the cranial cavity through the foramen ovale in dogs and humans, along with the mandibular branch of the trigeminal nerve [56, 99]. The sulcus that we observed in *B. physalus* may be homologous to the anteroexternal sulcus of *Mammalodon* [24] and others, but it is not in the same position. Two grooves that have been termed anterointernal sulci were described for the extinct cetacean *Waipatia* ([67], p. 158, fig. 11), although the specimen also possesses an anteroexternal sulcus. The location of the anterointernal sucli in *Waipatia* agrees with that of the sulcus that we observed in the extant balaenopterids identified above, and we suggest that the sulcus is homologous to one of the anterointernal sulci of *Waipatia*, given the similarities in

position [67]. Although there are few detailed dissections through the ear region of mysticetes, we suggest that the occupant of the anterointernal sulcus is the mandibular branch of the trigeminal nerve, given the relationship between the sulcus and the foramen ovale. The trigeminal nerve is the fifth cranial nerve, which separates into the opthalmic and maxillary branches for sensory input, and the mixed sensory and motor mandibular branch [99]. One of the anterointernal sulci in *Waipatia* may have transmitted the lesser petrosal nerve [67], which may also pass through the foramen ovale along with the trigeminal nerve.

TAXONOMIC DISTRIBUTION: In the plesiomorphic state that we observed in the balaenopteroids *B. edeni*, *B. omurai*, *B. musculus*, *B. physalus*, *Eschrichtius robustus*, and *Megaptera novaeangliae*, no anterointernal sulcus is developed on the anterior process. Apomorphic state 1, in which the anterointernal sulcus is present, is seen in *B. acutorostrata* and *B. bonaerensis*. The sulcus is evident as a broad and distinct groove extending in an anterior-posterior direction on the medial side of the anterior process in *B. acutorostrata*, and the sulcus is variably developed (0/1) in *B. physalus* and *B. borealis*. In some individuals of *B. borealis*, the sulcus is positioned more ventrally than in others.

HISTORICAL USAGE: This character was based on previously described morphology ([67], p. 158, fig. 11).

Character 22– Petrosal, anterior process with prominent, thin, bony projections.
STATES: 0= absent; 1= present.

DESCRIPTION: The petrosal of some mysticetes possesses several thin, bony projections along the anterior margin of the anterior process (apomorphic state 1), which are absent in the plesiomorphic state.

TAXONOMIC DISTRIBUTION: The plesiomorphic state is seen in most extinct and extant mysticetes. In the apomorphic condition, which is an autapomorphy for *Balaenoptera omurai* among extant species, several thin bony projections are developed along the anterior margin of the anterior process in some undescribed mysticete fossils.

HISTORICAL USAGE: This character has not been previously used in phylogenetic analyses.

Character 23– Petrosal, anteroexternal sulcus.
STATES: 0= present; 1= reduced or absent.

DESCRIPTION: The vascularization through the ear region of cetaceans and close terrestrial relatives of Cetacea has garnered interest among the scientific community [68], particularly on account of the systematic value of osteological correlates of blood vessels. In some mysticetes, there is development of a distinct groove that likely is homologous to the anteroexternal sulcus observed in the extinct odontocete *Waipatia* [67] and the toothed mysticete *Mammalodon* [24]. The well-developed sulcus is present in the plesiomorphic state anterior to the lateral projection of the anterior process of the petrosal. The groove runs between the petrosal and squamosal, and it follows a course

that begins near the junction of the pars cochlearis and posterior border of the anterior process, and it terminates at the anterior pedicle. The groove is absent in the apomorphic state. The occupant of the sulcus is unknown owing to the paucity of detailed dissections and descriptions of the nerves and blood vessels that cross through the ear region of mysticetes. However, two possibilities are the capsuloparietal emissary vein [68], which is a major vessel that branches from the sigmoid sinus [100] and drains the cranial cavity in the postglenoid region of the skull, and the middle meningeal artery [24], which is a major supplier of blood to the meninges [99].

TAXONOMIC DISTRIBUTION: The plesiomorphic state is observed in the extinct *Mammalodon colliveri* and the extant *Eschrichtius robustus*. The groove is present in some neonate balaenopterids, including *B. acutorostrata* (SDSU S970) and *B. physalus* (LACM 72507), but the groove is absent in adults of those species. However, the groove is present in both neonate (SDSNH 23762) and adult (SDSNH 24316) *E. robustus* individuals. Because the sulcus is absent in adults of *B. acutorostrata* and *B. physalus*, the taxa are scored as possessing the apomorphic state. In the derived condition, the anteroexternal sulcus is not well developed, which characterizes by most balaenids and balaenopterids (excluding neonatal *B. acutorostrata* and *B. physalus*). The presence or absence of the groove in *Eomysticetus whitmorei* could not be determined.

HISTORICAL USAGE: This character was based previously described morphology [67-68] and was previously used in phylogenetic analyses ([24], character 82; [49], character 206).

Character 24– Petrosal, dorsal junction between tegmen tympani and pars cochlearis extended dorsally as robust pyramidal process.
STATES: 0= absent; 1= present.

DESCRIPTION: In the plesiomorphic state, there is no dorsal extension of the tegmen tympani and pars cochlearis. Rather, the connection between the dorsal surface of the anterior process and the pars cochlearis is relatively flat or else the pars cochlearis is elevated above the tegmen tympani, as seen in *Balaenoptera musculus*. In the apomorphic state, the junction between the tegmen tympani and pars cochlearis on the dorsal side of the petrosal is extended as a robust pyramidal process, towards which both the tegmen tympani and pars cochlearis contribute.

TAXONOMIC DISTRIBUTION: The plesiomorphic state is observed in *Eomysticetus whitmorei*, *Mammalodon colliveri*, and balaenopterids. A robust pyramidal process (apomorphic state) is observed in balaenids.

HISTORICAL USAGE: This character has not been previously used in phylogenetic analyses.

Character 25– Petrosal, orientation of posterior process.
STATE: 0= posterolaterally relative to long axis of pars cochlearis; 1= at right angles to pars cochlearis.

DESCRIPTION: In the plesiomorphic state, the posterior process of the petrosal is posterolaterally oriented relative to the long axis of the pars cochlearis (promontorium). In this condition, the posterior process extends in line with the long axis of the promontorium. In the apomorphic state, the posterior process is oriented at a right angle to the pars cochlearis.

TAXONOMIC DISTRIBUTION: The plesiomorphic state is characteristic of most mysticetes, although the apomorphic state is observed in balaenids and neobalaenids. The posterior process is missing from isolated petrosals of *Mammalodon colliveri* (Fitzgerald, 2010).

HISTORICAL USAGE: This character was previously used in phylogenetic analyses 11: character 22; 12: character 23; 23: character 108; 24: character 110].

Character 26– Petrosal, posterior cochlear crest.
STATES: 0= absent or reduced; 1= present, medially extending shelf adjacent to fenestra cochleae; 2= present, ventrally directed.

DESCRIPTION: The posterior cochlear crest (sensu Fordyce [66], p. 205, fig. 15) extends posterolaterally from the bridge of bone separating the fenestrae cochleae and vestibuli (termed the crista interfenestralis). The structure often is referred to as the 'caudal tympanic process' in cetacean literature ([110, fig. 3), but this can be confused with a non-homologous structure on the posterior portion of the petrosal that is called the caudal tympanic process of terrestrial mammals ([62], p. 50; [64], p. 170; [101], p. 8; Mead and Fordyce [25] provide further discussion of this terminology). Because of this, we prefer 'posterior cochlear crest' to avoid confusion and eliminate any potential assertions of homology between the two evolutionarily independent structures. In the plesiomorphic state, the area between the two fenestrae is smooth or with a small bump, but not a distinct posterior cochlear crest. In apomorphic state 1, the posterior cochlear crest is large, in some cases forming a shelf. In apomorphic state 2, the posterior cochlear crest is large and ventrally directed. In the apomorphic conditions, the posterior cochlear crest may join with the tympanohyal on the posterior process, thereby forming a bony channel for the hyomandibular branch of the facial nerve.

TAXONOMIC DISTRIBUTION: The ancestral condition characterizes *Eomysticetus whitmorei*. Apomorphic state 1 characterizes the interfenestral region of *Balaenoptera*, *Megaptera novaeangliae*, and Balaenoidea, and apomorphic state 2 is observed in *Eschrichtius robustus*.

HISTORICAL USAGE: This character was previously used in phylogenetic analyses ([73], character 36).

Character 27– Petrosal, transverse elongation of pars cochlearis.
STATES: 0= absent; 1= present.

DESCRIPTION: The elongation of the pars cochlearis is best expressed as a ratio between the width and longitudinal length of the promontorium (which forms the ventral aspect of the pars cochlearis). The length of the promontorium was measured from the

fenestra cochleae to the anteriormost extent of the promontorium, and the width was measured as the longest axis of the promontorium, perpendicular to the width. In the plesiomorphic state, the ratio of transverse width to longitudinal length of the pars cochlearis is between 0.5 and 1.0. Ancestrally, the promontorium is globular, and it almost appears as though it is a separate element fused to the remainder of the pars cochlearis. In the apomorphic state, the width-length ratio is much greater than 1 and often closer to 2 [11], and the promontorium flares at its anterolateral aspect. The transverse elongation of the pars cochlearis is associated with a ventral flattening of the promontorium in some taxa (e.g., *Balaenoptera acutorostrata*), although the promontoria of *Balaenoptera musculus* and *Balaenoptera physalus* are markedly more convex in their ventral aspect.

TAXONOMIC DISTRIBUTION: The plesiomorphic state is observed in Balaenoidea and the apomorphic state is observed in Balaenopteroidea.

HISTORICAL USAGE: This character was previously used in phylogenetic analyses ([11], character 12; [12], character 18; [22], character 61; [23], character 92; [24], character 98; [49], character 230; [59], character 12; [69], character 50; [70], character 84; [71], character 77; [72], character 220; [73], character 97).

Character 28– Petrosal, attachment for tensor tympani muscle.
STATES: 0= present as enlarged fossa; 1= present as groove; 2= absent or poorly developed.

DESCRIPTION: The tensor tympani muscle originates on the ventral surface of the petrosal and inserts onto the malleus [56, 102]. When contracted, the muscle pulls on the malleus which tenses the tympanic membrane. In odontocetes, the tensor tympani muscle originates on the accessory ossicle [17, 25]. However, the accessory ossicle is not present in mysticetes. The accessory ossicle is fused embryonically with the anterior process in mysticetes, and tensor tympani muscle originates from a “furrow between the [anterior] process and cochlear sphere” on the mysticete petrosal ([17], p. 51). The tensor tympani muscle fossa of the plesiomorphic state is a large and oval depression situated between the anterior process of the petrosal and the anterior edge of the promontorium. The attachment site of the tensor tympani in apomorphic state 1 is a well defined groove, which is relatively small compared to the oval fossa observed in the plesiomorphic state. The attachment for the muscle is absent or very poorly developed in apomorphic state 2.

TAXONOMIC DISTRIBUTION: The plesiomorphic state is present in *Balaenoptera omurai*, and *Mammalodon colliveri*, *Eomysticetus whitmorei*, and balaenoids exhibit apomorphic state 1. Although the tensor tympani muscle is present in other mysticetes (at least *Balaenoptera acutorostrata* ([41], pl. 5), the muscle does not leave a trace on the petrosals of most balaenopterids (apomorphic state 2).

HISTORICAL USAGE: This character was previously used in phylogenetic analyses ([9], character 18; [11], character 7; [12], character 15; [22], character 56; [23], character 98; [24], character 88; [49], character 217; [60], character 48; [68], character 5; [69],

character 52; [71], character 80; [95], character 4; [103], character 14; [104], character 54).

Character 29– Petrosal, promontorial groove on medial side of pars cochlearis (promontorium).
STATES: 0= absent; 1= present.

DESCRIPTION: In many extant mysticetes, a groove is situated on the medial side of the promontorium, where it takes a sinuous and transverse course. Presence of such a groove is the apomorphic state, and an absence of the structure is considered to be plesiomorphic. The occupant of the groove has been reconstructed as the inferior petrosal sinus [2], but the sulcus may have carried the internal carotid artery, as seen in other ungulates [9].

TAXONOMIC DISTRIBUTION: We observed the plesiomorphic state in *Balaenoptera physalus* and *Eschrichtius robustus*. The groove is present (apomorphic state) on the petrosal of *Eubalaena glacialis*, and a groove is figured (but unlabeled) for *Mammalodon colliveri* ([24], fig. 23-24). We interpret the sulcus as the medial promontorial groove in *Mammalodon* as observed in other mysticetes.

HISTORICAL USAGE: This character was previously used in phylogenetic analyses ([2], character 51; [9], character 31; [11], character 13; [12], character 20; [22], character 62; [23], character 99; [60], character 49; [69], character 53; [71], character 81).

Character 30– Petrosal, ridge on anterolateral side of pars cochlearis, in ventral view.
STATES: 0= absent; 1= present and high, forms an anterior-posterior ridge; 2= present and well-developed.

DESCRIPTION: In the plesiomorphic state, the pars cochlearis is smooth with no development of a ridge on the anterolateral side. In apomorphic state 1, a high anterior-posteriorly oriented ridge is present on the anterolateral side of the pars cochlearis that also forms the medial ledge for a trough for the tensor tympani muscle. In apomorphic state 2, a well-developed ridge extends ventrally from the posterior half of the promontorium, immediately anterior to the level of the fenestra vestibuli and parallel to a groove for the tensor tympani muscle.

TAXONOMIC DISTRIBUTION: The plesiomorphic state is seen in species of *Balaenoptera*, and no ridge is developed along the anterolateral edge of the promontorium. Apomorphic state 1 is seen in extant balaenids, and apomorphic state 2 is only observed in *Eschrichtius robustus* among extant mysticetes. The states for *Mammalodon colliveri* and *Eomysticetus whitmorei* could not be determined.

HISTORICAL USAGE: This character was previously used in phylogenetic analyses ([12], character 11; [24], character 92).

Character 31– Petrosal, stylomastoid fossa.

STATES: 0= present, enlarged and extending to the pars cochlearis; 1= present, extending to the pars cochlearis and posterior process.

DESCRIPTION: The stylomastoid fossa is a depression that develops on the posteromedial surface of the promontorium adjacent to the fenestra cochleae. The fossa is absent ancestrally, and rather it is a neomorphic structure for Mysticeti. A stylomastoid fossa is present and forms a small square-shaped area in the plesiomorphic state, but the fossa extends laterally onto the base of the posterior process in the apomorphic condition.

TAXONOMIC DISTRIBUTION: We observed the plesiomorphic condition of the stylomastoid fossa in most mysticetes, although the depression is expanded (apomorphic state) in *Balaenoptera musculus* and *Balaenoptera physalus*. The state from *Mammalodon colliveri* could not be determined.

HISTORICAL USAGE: This character was previously used in phylogenetic analyses 11: character 14; 23: character 106].

Character 32– Petrosal, perilymphatic foramen in dorsolateral aspect.
STATES: 0= widely separated from fenestra cochleae; 1= narrowly separated from fenestra cochleae; 2= confluent with fenestra cochleae.

DESCRIPTION: Separation of the perilymphatic foramen (for transmission of the perilymphatic duct) and fenestra cochleae (covered by a secondary tympanic membrane) occurs in most mammals, including most mysticetes. The two openings are widely separated in the plesiomorphic state, but the openings are more closely spaced in apomorphic state 1. At the extreme, the perilymphatic foramen is confluent with the fenestra cochleae by a broad groove in apomorphic state 2. The confluence between the fenestra cochleae and perilymphatic foramen in proboscideans was hypothesized to be related to low-frequency hearing [105]. Eschrichtiids are likely sensitive to low-frequency vibrations [106], given that they vocalize using low frequency and even infrasonic sounds. However, all mysticetes are likely sensitive to low frequency sounds (although no audiogram data are available for mysticetes). A more thorough study of the ear regions of mysticetes, as well as other taxa in which the perilymphatic foramen is confluent with the fenestra cochleae (i.e., proboscideans and sirenians) would be useful in uncovering the relationship, if any, between auditory physiology and the perilymphatic foramen.

TAXONOMIC DISTRIBUTION: Wide separation of the perilymphatic and the fenestra cochleae (plesiomorphic state) occurs in *Mammalodon colliveri*, *Megaptera novaeangliae* and balaenids. The bony separation between the two foramina is very narrow (apomorphic state 1) in most other mysticetes, although in the extreme case of *Eschrichtius robustus*, the perilymphatic foramen is confluent with the fenestra cochleae by a broad groove (apomorphic state 2). In this condition, the bony separation between the perilymphatic duct and the fenestra rotunda does not ossify, which is convergent (or a reversal) to the state early non-therian mammals [63]. Among extant mammals, the perilymphatic foramen and fenestra cochleae are confluent in sirenians and proboscideans

[105; 107] in addition to *E. robustus*. A much narrower and slit-like confluence occurs in some specimens of immature *Balaenoptera physalus*, although the perilymphatic foramen is separate from the fenestra cochleae in most *B. physalus* specimens. *Balaenoptera physalus* is scored as polymorphic (1/2) in the present matrix.

HISTORICAL USAGE: This character was previously used in phylogenetic analyses ([11], character 21; [12], character 22; [23], character 95; [49], character 228; [60], character 51; [69], character 55).

Character 33– Petrosal, suprameatal fossa.
STATES: 0= present; 1= absent.

DESCRIPTION: The suprameatal region of the petrosal is situated on the dorsal surface of the pars cochlearis, dorsal to the internal auditory meatus. In the plesiomorphic state, the suprameatal region forms a broad concavity known as the suprameatal fossa, although the suprameatal area is flat or convex without a broad and shallow fossa in the apomorphic state.

TAXONOMIC DISTRIBUTION: A suprameatal fossa is present (plesiomorphic state) in *Eomysticetus whitmorei*, *Caperea marginata*, *Eschrichtius robustus*, and *Balaenoptera acutorostrata*. The apomorphic state (fossa absent) is characteristic of all remaining balaenopterids and balaenids, as well as *Mammalodon colliveri*, in which the suprameatal area is convex [24].

HISTORICAL USAGE: This character was previously used in phylogenetic analyses ([2], character 4; [9], character 7; [11], character 18; [22], character 67; [60], character 50; [71], character 71).

Character 34– Petrosal, suprameatal region.
STATES: 0= at the level of the anterior process; 1= elevated above (dorsal) to the anterior process.

DESCRIPTION: The suprameatal region of the petrosal is situated on the dorsal surface of the pars cochlearis, dorsal to the internal auditory meatus. The region is elevated with respect to the dorsal surface of the anterior process in the apomorphic state, so that there is a distinct dorsolateral surface to the pars cochlearis. The suprameatal region of the petrosal is not elevated in the plesiomorphic state.

TAXONOMIC DISTRIBUTION: The plesiomorphic state, in which the suprameatal region is not elevated dorsally, characterizes *Mammalodon colliveri*, *Eomysticetus whitmorei*, balaenids, and some balaenopterids (*B. bonaerensis*, *B. borealis*, and *B. edeni*). Note that in balaenids, the dorsolateral border of the pars cochlearis contributes to the dorsal pyramidal extension of the petrosal. The apomorphic state is observed in some balaenopteroid taxa (*B. acutorostrata*, *B. musculus*, *B. physalus*, *E. robustus*, and *M. novaeangliae*).

HISTORICAL USAGE: This character was previously used in phylogenetic analyses ([69], character 54).

Character 35– Petrosal, crista transversa.
STATES: 0= low, does not reach cerebral surface; 1= high, reaches cerebral surface.

DESCRIPTION: The crista transversa is a crest of bone that sits within the internal acoustic meatus and separates the internal opening of the facial nerve (CN VII) canal from the opening for the vestibulocochlear nerve (CN VIII). In the plesiomorphic state, the crista transversa is low and does not reach the cerebellar surface of the petrosal, forming a very deep internal acoustic meatus. In the apomorphic state, the crista transversa is high and extends to the cerebral surface. Although both states are observed across Mysticeti, the depth of the crista transversa within the internal acoustic meatus may change throughout ontogeny [20].

TAXONOMIC DISTRIBUTION: The ancestral condition characterizes *Eomysticetus whitmorei*, Balaenidae, *Megaptera novaeangliae* and some *Balaenoptera* species (namely *B. borealis*, *B. edeni*, and *B. musculus*). The apomorphic state is seen in *Caperea marginata*. The condition of the crista transversa is variably developed in *Eschrichtius robustus* and *Megaptera novaeangliae* (0/1).

HISTORICAL USAGE: This character was previously used in phylogenetic analyses ([11], character 19; [12], character 29; [23], character 102).

Character 36– Petrosal, fenestra cochleae size relative to fenestra vestibuli.
STATES: 0= small; 1= large.

DESCRIPTION: Two openings penetrate the promontorium and open into the inner ear cavities, which are the fenestra vestibuli for the accommodation of the footplate of the stapes, and the fenestra cochleae, which is covered by a secondary tympanic membrane in life. The relative size of each opening is determined by measuring the longest diameters of the two openings. In the plesiomorphic state, the fenestra cochleae is subequal in size or smaller than the fenestra vestibuli. In the apomorphic state, the fenestra cochleae is larger than the fenestra vestibuli.

TAXONOMIC DISTRIBUTION: The plesiomorphic condition is observed in most balaenopteroids, but the apomorphic state is seen in *Eomysticetus whitmorei*, *Megaptera novaeangliae*, *Caperea marginata*, and balaenids.

HISTORICAL USAGE: This character was based on previously described morphology ([108], p. 53).

Character 37– Petrosal, length of promontorium relative to length of anterior process.
STATES: 0= promontorium length nearly equivalent to anterior process length; 1= promontorium length 35-70% of anterior process length.

DESCRIPTION: The anteroposterior length of the promontorium was measured from the fenestra cochleae to the anteriormost termination of the promontorium. The length of the anterior process was measured from the facial sulcus to the apex of the anterior process

(Table S6). In the plesiomorphic state, the promontorium length approximates the anterior process length. In the apomorphic state, the promontorium length is 35-70% of the anterior process length.

TAXONOMIC DISTRIBUTION: The plesiomorphic condition is observed in *Eschrichtius robustus*, and the apomorphic state is characteristic of most balaenoids and balaenopteroids.

HISTORICAL USAGE: This character has not been used previously in phylogenetic analyses.

Character 38– Petrosal, arrangement of endolymphatic and perilymphatic foramina.
STATES: 0= separate, with thin bony septum that is anterior-posteriorly oriented; 1= *en echelon*, divided by a thin bony septum that is dorsoventrally oriented.

DESCRIPTION: The endolymphatic (also known as the ductus endolymphaticus [1] or vestibular aqueduct [25]) and perilymphatic ducts travel in separate bony canals that open on the endocranial surface of the petrosal through individual foramina. The morphology and arrangement of the foramina varies among extinct and extant mysticetes. In the plesiomorphic state, the foramina are separate and distinct from one another. In the apomorphic state, the cranial openings overlap on another in an en echelon arrangement divided by a thin bony septum dorsoventrally oriented.

TAXONOMIC DISTRIBUTION: The plesiomorphic state is seen *Eomysticetus whitmorei*, balaenids, *Balaenoptera acutorostrata*, and *Balaenoptera omurai*. The apomorphic state is observed in *Caperea marginata*, *Megaptera novaeangliae*, *Eschrichtius robustus*, and the remaining species of *Balaenoptera*.

HISTORICAL USAGE: This character has not been previously used in phylogenetic analyses.

Character 39– Petrosal, distance between apertures for endolymphatic foramen and fenestra cochleae.
STATES: 0= very narrow; 1= narrow; 2= very wide.

DESCRIPTION: The distance between the fenestra cochleae and endolymphatic foramen varies among mysticetes, and the states for this character can be quantified by comparing the measurement with the distance between the fenestrae cochleae and vestibuli, or the interfenestral distance ([49], character 229). A very narrow separation that is less than 112% of the interfenestral distance characterizes the plesiomorphic state. In apomorphic state 1, the distance between fenestra cochleae and endolymphatic foramen is 121-185% of interfenestral distance. A very wide separation where the distance between openings are over 222% of the interfenestral distance describes apomorphic state 2.

TAXONOMIC DISTRIBUTION: The plesiomorphic state is observed in *Eschrichtius robustus*, and apomorphic state 1 is shared by *Caperea marginata* and species of *Balaenoptera* among extant taxa. The fenestra cochleae and endolymphatic foramen

have a very wide separation (apomorphic state 3) in *Balaena mysticetus* and *Eubalaena* sp.

HISTORICAL USAGE: This character was previously used in phylogenetic analyses ([24], character 97; [49], character 229).

Character 40– Petrosal, relative size of internal openings for vestibulocochlear and facial nerve canals within internal acoustic meatus.
STATES: 0= vestibulocochlear greater than facial nerve canal opening; 1= vestibulocochlear subequal to facial nerve canal opening.

DESCRIPTION: The internal openings for the facial and vestibulocochlear nerve canals are adjacent to one another within the internal acoustic meatus on the endocranial surface of the petrosal. In the plesiomorphic state, the opening for the vestibulocochlear nerve canal (cranial nerve VIII) is larger than the opening for the facial nerve canal (cranial nerve VII). In the apomorphic state, the openings for the vestibulocochlear and facial nerve canals are approximately the same size.

TAXONOMIC DISTRIBUTION: The plesiomorphic condition in which the opening for the vestibulocochlear nerve canal is greater than that for the facial nerve is seen in most extant mysticetes, as well as the two extinct outgroup taxa. On the other hand, the apomorphic condition is seen in several balaenopterids, including *Balaenoptera bonaerensis*, *Balaenoptera borealis*, *Balaenoptera physalus*, and *Balaenoptera musculus*.

HISTORICAL USAGE: This character has not been previously used in phylogenetic analyses.

Character 41– Petrosal, stapedial fossa and facial nerve sulcus.
STATES: 0= separate; 1= confluent.

DESCRIPTION: The stapedial fossa serves as the origin for the stapedius muscle, and it is situated posterior to the fenestra cochleae. The facial nerve sulcus is laterally adjacent to the stapedial fossa. In the plesiomorphic state condition, the stapedial fossa and facial nerve sulcus are separate structures, divided by a thin ridge. However, the structures are confluent in the apomorphic state.

TAXONOMIC DISTRIBUTION: The plesiomorphic state characterizes the petrosal of most species of *Balaenoptera*, *Balaena mysticetus*, and *Caperea marginata*. The derived condition is observed in *Balaenoptera borealis*, *Megaptera novaeangliae*, *Eschrichtius robustus*, and *Eubalaena glacialis*.

HISTORICAL USAGE: This character has not been previously used in phylogenetic analyses.

Character 42– Petrosal, size of stapedial fossa.
STATES: 0= small and compressed; 1= large/ hemispherical.

DESCRIPTION: The stapedial fossa serves as the origin for the stapedius muscle posterior to the fenestra cochleae. The muscle inserts on the stapes and rocks the bone when it contracts. In the plesiomorphic state, the stapedial fossa is small relative to the overall size of the promontorium. In the apomorphic state, the stapedial fossa is large and/or hemispherical when compared to the size of the promontorium.

TAXONOMIC DISTRIBUTION: The plesiomorphic condition is observed in *Balaenoptera acutorostrata*, *Balaenoptera bonaerensis*, and *Balaenoptera physalus*. The apomorphic state of a large fossa characterizes *Balaenoptera borealis*, *Balaenoptera edeni*, *Balaenoptera musculus*, *Eschrichtius robustus*, *Megaptera novaeangliae*, and balaenids.

HISTORICAL USAGE: This character has not been used previously in phylogenetic analyses.

Character 43– Petrosal, fossa for the malleus.
STATES: 0= present; 1= partially defined; 2= indistinct to absent.

DESCRIPTION: In mysticetes, the malleus typically is fused to the tympanic bulla, near the sigmoid process. However, the malleus articulates with the petrosal in some taxa, leaving a trace as a malleolar fossa lateral to the promontorium. The fossa for the malleus indicates articulation between the ossicle and the petrosal, although whether or not the ossicles themselves are functional from an auditory standpoint, as well as the transmission pathway of sound vibrations in mysticetes, is controversial [3, 15, 110]. A distinct fossa for the head of the malleus is the plesiomorphic state ([11], p. 1054), although the fossa is lost to varying degrees in many mysticetes. In apomorphic state 1, the fossa is partially defined along its medial edge, and the fossa is all together absent in apomorphic state 2.

TAXONOMIC DISTRIBUTION: The plesiomorphic state is seen in *Eomysticetus whitmorei*, but none of the extant mysticetes that we examined. In most balaenopteroids, the posterolateral edge of the fossa is well-defined; however, the medial edge is less distinct (apomorphic condition 1). In *Eschrichtius robustus* and balaenoids, the fossa for the malleus is poorly differentiated from other structures in the epitympanic region (apomorphic condition 2).

HISTORICAL USAGE: This character was based on previously described morphology [13] and was previously used in phylogenetic analyses ([2], character 6; [9], character 15; [11], character 9; [23], character 96; [68], character 4).

Character 44– Petrosal, contact edge between stylomastoid fossa and ventral surface of promontorium.
STATES: 0= not well-developed; 1= well-developed as a short keel; 2= well-developed as elongate, broad flange.

DESCRIPTION: This character describes the ventral definition of the stylomastoid fossa. In the ancestral condition, the contact between the stylomastoid fossa and the ventral surface of the promontorium is not well-developed. In apomorphic state 1, the edge is

well-developed as a short keel. This character state also applies to petrosals that display any degree of a sharp edge. In apomorphic state 2, the edge is well developed and extends as an elongate, broad flange. Although more detailed dissections through the ear region of mysticetes are needed before the occupant of the fossa can be identified, the stylomastoid fossa may accommodate part of the peribullary sinus or posterior sinus in life [4, 25].

TAXONOMIC DISTRIBUTION: In the ancestral condition seen in balaenids, *Caperea marginata*, *Eschrichtius robustus*, and *Eomysticetus whitmorei*. We observed apomorphic condition 1 in species of *Balaenoptera*, and apomorphic state 2 in *Megaptera novaeangliae*.

HISTORICAL USAGE: This character was previously used in phylogenetic analyses ([73], character 29).

Character 45– Petrosal, hiatus Fallopii.
STATES: 0= through ventral surface of promontorium; 1= within juncture between promontorium and anterior process; 2= through ventral surface of anterior process.

DESCRIPTION: The hiatus Fallopii, for passage of the greater petrosal branch of the facial nerve, opens in a variable position among mysticetes. In the plesiomorphic state, the hiatus is positioned on the ventral surface of the promontorium, medial to a distinct groove between the anterior process and promontorium. In apomorphic state 1, the hiatus Fallopii opens within the juncture between the promontorium and anterior process. The hiatus Fallopii is situated lateral to the juncture in apomorphic state 2.

TAXONOMIC DISTRIBUTION: The ancestral condition characterizes the balaenids, *Balaenoptera acutorostrata*, *Balaenoptera bonaerensis*, and *Balaenoptera musculus* (in which the hiatus opens on the anterior edge of the promontorium, similar to that in many terrestrial mammals [110]). Apomorphic state 1 is exhibited by *Eschrichtius robustus* and *Megaptera novaeangliae*, and apomorphic state 2 is seen in *Balaenoptera borealis* and *Balaenoptera edeni*.

HISTORICAL USAGE: This character has not been previously used in phylogenetic analyses.

Character 46– Petrosal, ventral flattening of promontorium.
STATES: 0= absent; 1= present.

DESCRIPTION: The promontorium houses the cochlea within the inner ear. In the plesiomorphic state, the promontorium develops as a convex structure on the ventral side of the petrosal. In the apomorphic state, the promontorium is flattened ventrally and does not form the typical domed structure observed in most mammals.

TAXONOMIC DISTRIBUTION: The promontorium is convex and rounded in archaic mysticetes such as *Mammalodon colliveri*, as well as extant taxa, including *Balaena mysticetus*, *Caperea marginata*, *Eubalaena* sp., *Megaptera novaeangliae*, *Balaenoptera musculus*, and *Balaenoptera physalus* (plesiomorphic condition). However, the

promontorium is ventrally flattened (apomorphic state) in remaining members of *Balaenoptera*. Coupled with the transverse, dorsomedial elongation of the promontorium, the flattening gives the pars cochlearis of these taxa a medially flaring appearance.

HISTORICAL USAGE: This character has not been previously used in phylogenetic analyses.

Character 47– Petrosal, endocranial opening of facial nerve canal.
STATES: 0= circular; 1= oval; 2= oval with anterior fissure.

DESCRIPTION: The shape of the internal opening of the facial nerve canal (within the internal acoustic meatus) varies among mysticetes. In the plesiomorphic state, the opening is circular, whereas the opening is oval in apomorphic state 1. In apomorphic state 2, the opening for the facial canal is oval with an anterior fissure.

TAXONOMIC DISTRIBUTION: The plesiomorphic state is expressed by *Mammalodon colliver*i, Balaenidae, *Balaenoptera borealis*, *Balaenoptera bonaerensis*, and *Megaptera novaeangliae*. We observed apomorphic condition 1 in all other balaenopterid species and *Caperea marginata*, and apomorphic condition 2 in *Eschrichtius robustus*. A similar condition to *E. robustus* was observed in a specimen of *Eubalaena glacialis* [11], but specimens of *E. glacialis* studied here (AMNH 169829; LACM 54763) lack the groove that is very distinct in *E. robustus*. The endocranial opening of the facial canal is circular in AMNH 169829, and we score *Eubalaena* sp. thusly (state 0), rather than as polymorphic. Geisler and Luo [11] argued that the anterior fissure of the endocranial opening of the facial canal transmitted the greater superficial petrosal nerve in life. The greater superficial petrosal nerve typically exits via an opening called the hiatus Fallopii on the anterior (e.g., *Balaenoptera musculus*) or ventral (e.g., *Balaenoptera acutorostrata*) surface of the petrosal in other cetaceans and non-cetacean mammals. Geisler and Luo did not identify the hiatus Fallopii for *E. glacialis*, but the structure is present along the lateral border of the promontorium in the *E. glacialis* specimens studied here. Furthermore, both the hiatus Fallopii and the anterior fissure of the internal opening of the facial canal are present in *E. robustus* (LACM 54541 and SDNHM 24316), so the greater superficial petrosal nerve likely did not travel within the anterior fissure. The occupant of the fissure is not known at the present time.

HISTORICAL USAGE: This character was previously used in phylogenetic analyses ([11], character 20; [12], character 30; [22], character 69; [23], character 105; [49], character 238; [59], character 20; [60], character 52; [69], character 56; [70], character 90; [71], character 75; [72], character 226).

Character 48– Petrosal, angle between posterior process and flange of ventrolateral tuberosity.
STATES: 0= obtuse; 1= approximately 90 degrees; 2= acute.

DESCRIPTION: The angle between the posterior process and the flange of the ventrolateral tuberosity varies among extant mysticetes. In the plesiomorphic state, the

angle is well over 90 degrees. In apomorphic state 1, the long axes of the processes form close to a right angle, but the condition in apomorphic state 2 is for the axes to form an acute angle.

TAXONOMIC DISTRIBUTION: The ancestral condition is seen in *Eomysticetus whitmorei* [111] and *Caperea marginata*. Apomorphic state 1 is exhibited by balaenopteroids, and apomorphic state 2 is observed in balaenids. The acute angle in balaenids appears to be associated with the posterior hypertrophication of the flange of the ventrolateral tuberosity.

HISTORICAL USAGE: This character was based on previously described morphology [112-113], and was previously used in phylogenetic analyses [111].

SUPPLEMENTARY REFERENCES (numbers 1 through 91 in text)

92. Tomilin AG (1957) Cetacea. In: Heptner VG, editor. Mammals of the USSR and adjacent countries. Volume 9. Jerusalem: Israel Program for Scientific Translation. pp. 1-717.

93. Muizon C de (1987) The affinities of *Notocetus vanbenedeni*, an early Miocene platanistoid (Cetacea, Mammalia) from Patagonia, southern Argentina. Am Mus Novit 2904: 1-27.

94. Uhen M (2004) Form, function, and anatomy of *Dorudon atrox* (Mammalia: Cetacea): an archaeocete from the middle to late Eocene of Egypt. U Mich Pap Paleontol 34: 1-222.

95. O'Leary MA, Geisler JG (1999) The position of Cetacea within Mammalia: phylogenetic analysis of morphological data from extinct and extant taxa. Syst Biol 48: 455-490.

96. Lancaster WC (1990) The middle ear of Archaeoceti. J Vertebr Paleontol 19:117-127.

97. Bisconti M (2003) Evolutionary history of Balaenidae. Cranium 20: 9-50.

98. Bisconti M (2008) Morphology and phylogenetic relationships of a new eschrichtiid genus (Cetacea: Mysticeti) from the early Pliocene of northern Italy. Zool J Linn Soc 153: 161-186.

99. Evans HE (1993) Miller's anatomy of the dog (third edition). Philadelphia: Saunders. 1113 p.

100. Padget DH (1957) The development of the cranial venous system in man, from the viewpoint of comparative anatomy. Contrib Embryol 36: 81-151.

101. Wible JR, Rougier GW, Novacek MJ, McKenna MC (2001) Earliest eutherian ear region: a petrosal referred to *Prokennalestes* from the Early Cretaceous of Mongolia. Am Mus Novit 3322: 1-44.

102. Fleischer G (1978) Evolutionary principles of the mammalian middle ear. Berlin: Springer-Verlag. 70 p.

103. Geisler JH (2001) New morphological evidence for the phylogeny of Artiodactyla, Cetacea, and Mesonychidae. Am Mus Novit 3344: 1-53.

104. Thewissen JGM, Williamson EM, Roe LJ, Hussain ST (2001) Skeletons of terrestrial cetaceans and the relationship of whales to artiodactyls. Nature 413: 277-281.

105. Court N (1994) The periotic of *Moeritherium* (Mammalia, Proboscidea): homology or homoplasy in the ear region of Tethytheria McKenna, 1975?. Zool J Linn Soc 112: 13-28.

106. Erbe C (2002) Hearing capabilities of baleen whales. Def R&D Can Contr Rep 2002-65: 1-28.

107. Fischer MS (1990) Un trait unique de l'orelle des elephants et des siréniens (Mammalia): un paradoxe phylogénétique. C R Acad Sci Ser II 311: 157-162.

108. Decker DM, Wozencraft WC (1991) Phylogenetic analysis of recent procyonid genera. J Mammal 72: 42-55.

109. Norris JC (1981) Auditory morphology in whales and dolphins. MS Thesis, San Diego State University. 128 p.

110. Cifelli RL (1982) The petrosal structure of *Hyopsodus* with respect to that of some other ungulates, and its phylogenetic implications. J Paleontol 56: 795-805.

111. Churchill M (2007) The systematics and biogeography of the Balaenidae (Cetacea: Mysticeti). MS Thesis, San Diego State University. 178 p.

112. Miller GSJ (1924) A Pollack whale from Florida presented to the National Museum by the Miami Aquarium Association. Proc US Nat Mus 66: 1-15.

113. Fitzgerald EMG (2005) Pliocene marine mammals from the Whalers Bluff Formation of Portland, Victoria, Australia. Mem Mus Vict 62: 67-89.
